# Impact of supervised physical exercise on inflammatory and oxidative stress biomarkers in older adults: a systematic review and meta-analysis of randomized controlled trials

**DOI:** 10.1093/geront/gnag228

**Published:** 2026-09-24

**Authors:** Chenhui Chenhuichen, Yasmin Ezzatvar, Fabiola Zambom-Ferraresi, Carly Welch, Myriam Gorospe, Marta Montes, Marta Gutiérrez-Valencia, Juan José Lasarte, Thomas Jackson, Nicolás Martínez-Velilla

**Affiliations:** Navarre Public University (UPNA), Pamplona, Navarre, Spain; Department of Geriatrics, Navarre Health Service (SNS-O), Navarre University Hospital (HUN), Pamplona, Navarre, Spain; Navarrabiomed, Pamplona, Navarre, Spain; Navarra Institute for Health Research (IdiSNA), Pamplona, Navarre, Spain; Lifestyle factors with impact on Ageing and overall Health (LAH) Research Group, Department of Nursing, University of València, Valencia, Spain; Vicerrectoría de Investigación y Postgrado, Universidad de Los Lagos, Osorno, Chile; Navarrabiomed, Pamplona, Navarre, Spain; Department of Twin Research and Genetic Epidemiology, School of Life Course and Population Sciences, Faculty of Life Sciences and Medicine, King’s College London, London, United Kingdom; Department of Ageing and Health, NHS Foundation Trust, Guy’s and St Thomas Westminster Bridge, London, United Kingdom; Laboratory of Genetics and Genomics, National Institute on Aging, NIH, Baltimore, Maryland, United States; Navarra Institute for Health Research (IdiSNA), Pamplona, Navarre, Spain; RNA and senescence Group, Center for Applied Medical Research, University of Navarra, Pamplona, Navarre, Spain; Cancer Center University of Navarra Clinic (CCUN), Pamplona, Navarre, Spain; Navarra Institute for Health Research (IdiSNA), Pamplona, Navarre, Spain; Innovation and Organization Unit, Navarre Health Service (SNS-O), Pamplona, Navarre, Spain; Navarra Institute for Health Research (IdiSNA), Pamplona, Navarre, Spain; Cancer Center University of Navarra Clinic (CCUN), Pamplona, Navarre, Spain; Immunology and Immunotherapy Program, Center for Applied Medical Research, University of Navarra, Pamplona, Navarre, Spain; Department of Inflammation and Ageing, School of Infection, Inflammation and Immunology, College of Medicine and Health, University of Birmingham, Birmingham, United Kingdom; Department of Healthcare for Older People, Queen Elizabeth Hospital Birmingham, Birmingham, United Kingdom; Navarre Public University (UPNA), Pamplona, Navarre, Spain; Department of Geriatrics, Navarre Health Service (SNS-O), Navarre University Hospital (HUN), Pamplona, Navarre, Spain; Navarrabiomed, Pamplona, Navarre, Spain; Navarra Institute for Health Research (IdiSNA), Pamplona, Navarre, Spain; CIBER of Frailty and Healthy Aging (CIBERFES), Instituto de Salud Carlos III, Madrid, Spain

**Keywords:** Physical exercise, Inflammation, Oxidative stress, Systematic review

## Abstract

**Background and Objectives:**

Physical exercise is widely recognized as an effective non-pharmacological intervention that improves not only functional capacity but also cognition and quality of life in older adults. However, the molecular pathways through which it influences aging-related processes remain incompletely understood. This systematic review and meta-analysis aimed to synthesize current evidence on the effects of structured physical exercise intervention on molecular biomarkers associated with inflammation and oxidative stress in older adults.

**Research Design and Methods:**

Randomized controlled trials that included participants ≥65 years old were identified through searches of PubMed, Scopus, Web of Science, Cumulative Index to Nursing and Allied Health Literature, and the Cochrane Library.

**Results:**

A total of 50 reports (4,379 participants) were included in the qualitative synthesis, and 38 of them in the different meta-analyses performed. Exercise interventions ranged from 8 weeks to 12 months and included aerobic, resistance, and multicomponent training. Pooled analyses indicated modest but significant reductions in C-Reactive Protein (CRP) and high-sensitivity CRP, as well as increases in Interleukin (IL)-10. Effects on other pro-inflammatory cytokines, including IL-6 and TNF-α, were inconsistent across studies. Evidence from a limited number of trials (*n* = 6) also suggested improvements in redox balance.

**Discussion and Implications:**

Overall, structured physical exercise demonstrates a moderate immunomodulatory and antioxidant effect in older adults, although bias was moderate to high. PROSPERO registry: CRD420251006248.

## Graphical abstract

**Graphical Abstract:**
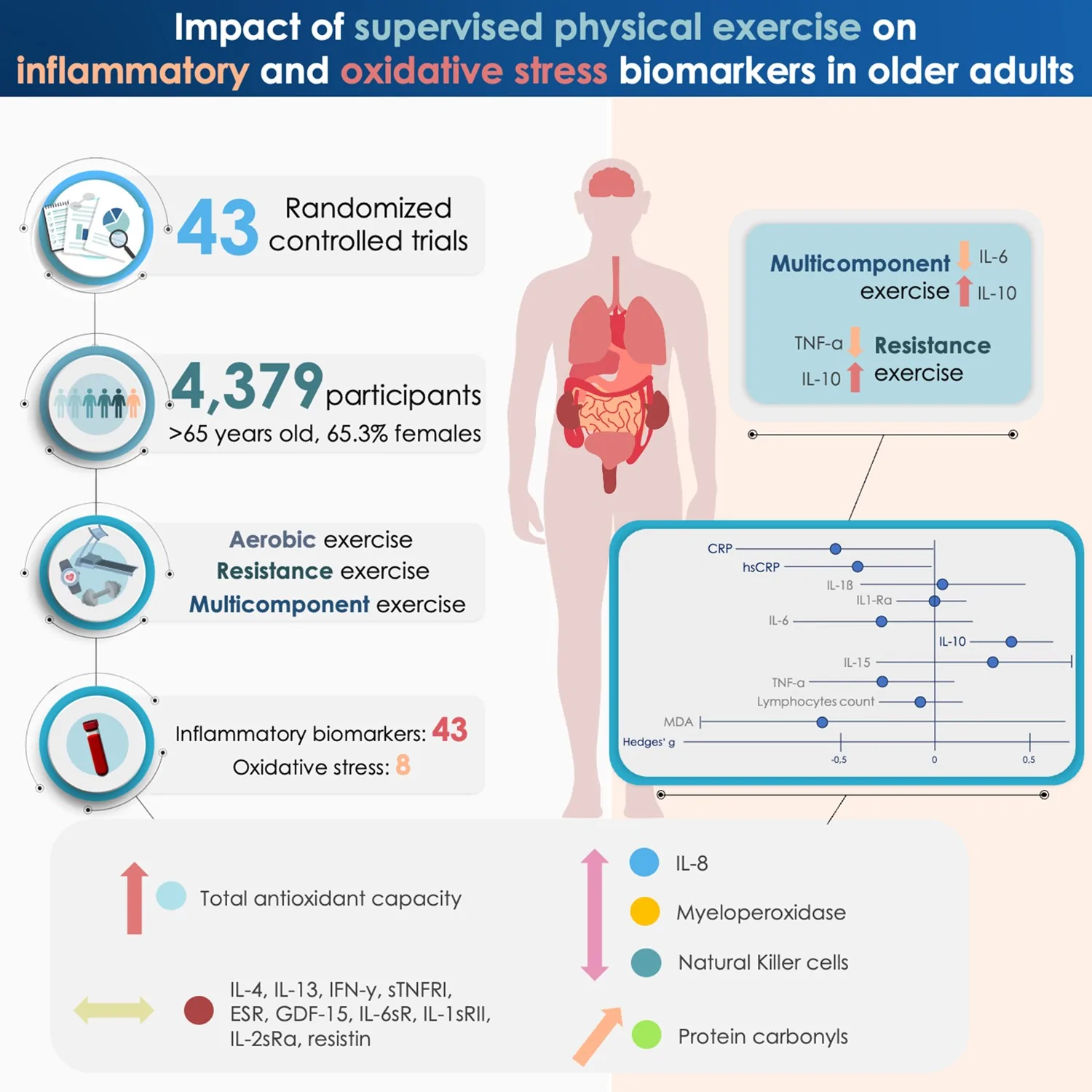

## Introduction

The global population of older adults is expanding at an extraordinary rate worldwide (Foreman et al., 2018), mainly due to advances in medicine and public health. However, increased longevity does not necessarily translate into sustained health or functional ability. Extending health span, not just lifespan, requires a deep understanding of the fundamental mechanisms underlying biological aging (Bautmans et al., 2022).

Among strategies to preserve function and promote healthy aging, structured physical exercise has emerged as one of the most effective non-pharmacological interventions, improving functional capacity (Wei et al., 2025), cognitive function (Biazus-Sehn et al., 2020), and quality of life of older persons with consequent gains in independence (Zhu et al., 2025). Moreover, it is increasingly recognized as a key strategy to counteract multiple declines associated with aging, such as deregulated nutrient sensing, mitochondrial dysfunction, cellular senescence, and chronic inflammation (Kong et al., 2025; Rebelo-Marques et al., 2018).

Despite accumulating evidence supporting the beneficial effects of physical exercise on health and functional capacity in older adults, the specific underlying molecular mechanisms driving these outcomes remain incompletely elucidated (Lavin et al., 2022). Heterogeneity in study designs, intervention modalities, and biomarker selection has contributed to inconsistent findings, ultimately hindering the development of optimized and personalized exercise prescription for specific populations. A better understanding of how exercise influences molecular pathways is therefore essential to align clinical recommendations with biological mechanisms.

One of the underlying mechanisms linking aging to increased disease vulnerability is inflammaging, a state of chronic, low-grade systemic inflammation that develops with aging, even in the absence of infection or obvious disease. This phenotype results from the interaction of complex factors such as immunosenescence, environmental stressors, and genetic predisposition (Aranda et al., 2025). Immunosenescence, the term used to describe age-related changes in the immune system, is characterized by features like thymic involution, imbalances in naïve/memory cell ratios, metabolic dysfunction, and genetic alterations (Liu et al., 2023). Inflammaging and immunosenescence appear deeply interconnected, and some authors argue that certain features of immunosenescence might represent an adaptive and necessary response to limit damage in the older organism, rather than purely detrimental decline (Fulop et al., 2017). In parallel, oxidative stress, also related to mortality risk and worsening clinical outcomes (Semba et al., 2007; Vazquez-Agra et al., 2022), contributes to both processes by increasing reactive oxygen and nitrogen species and damaging cellular components, ultimately fostering a state described as oxy-inflammaging (Fulop et al., 2017).

This systematic review aims to synthesize the current evidence regarding the impact of structured physical exercise intervention on key molecular markers, pathways, and cellular processes associated with inflammation and oxidative stress in older adults (aged 65 years and older).

## Methods

### Protocol

This systematic review was conducted in accordance with the Preferred Reporting Items for Systematic Reviews and Meta-Analyses guidelines (Page et al., 2021) (Table S1). It was also preregistered in the PROSPERO database under the identifier CRD420251006248, and no modifications to the registered protocols were made during the review process. The search was conducted in February 2025 across the following databases: PubMed, Cochrane Library, Scopus, Cumulative Index to Nursing and Allied Health Literature, and Web of Science. Retrieved records were imported into Covidence (Covidence systematic review software, Veritas Health Innovation, Melbourne, Australia), where duplicates were automatically removed. Two authors (C.C. and F.Z.F.) independently assessed the records based on the predefined inclusion and exclusion criteria, screening titles, and abstracts in a first stage, and full text of references considered for inclusion in the second stage. Disagreements were resolved through discussion with a third author (N.M.V.). The search terms used in each database are detailed in Supplementary File (Tables S2–S6).

### Inclusion and exclusion criteria

Studies were included in the review if the following criteria were met: (a) study type: randomized controlled trials; (b) participants: older adults aged ≥65; (c) intervention: any type of structured physical exercise; (d) comparison: usual care, health education or sham exercise (e.g., stretch, balance); (e) outcomes: any biomarker related to the aging process measured in biological samples. Only studies written in English or Spanish were included. No restrictions on publication date or specific health conditions were applied.

### Data extraction

Two authors (C.C. and F.Z.F.) independently extracted relevant data from all the included studies, including: author names, year of publication, country, number of participants, sex/gender, mean age and standard deviation (SD), inclusion and exclusion criteria, intervention description (duration, type, intensity, frequency), description of the comparison, and results of all the relevant variables to be evaluated in the present review. For those articles without sufficient information, authors were contacted to request the missing data.

### Risk of bias

Two authors (C.C. and F.Z.F.) independently evaluated the risk of bias of the biological outcome measures across all included studies using the Cochrane Risk of Bias tool 2.0 (Sterne et al., 2019). This tool evaluates potential bias arising from the randomization process, deviations from intended intervention, missing outcome data, measurement of the outcome, and selection of the reported results. Any discrepancies were resolved through discussion with a third reviewer (N.M.V.). Given the biological nature of the results, a single risk of bias assessment was performed for all evaluated blood outcomes, as they were deemed to share the same risk across all domains; thus, the RoB2 was reported jointly.

### Data synthesis and statistical analysis

We performed meta-analyses for outcomes reported in three or more studies, using random-effects models with Hedges’ g as the effect size measure. We prioritized the extraction of mean change scores and their corresponding SDs directly from the original publications. When these change metrics were not directly reported, they were either imputed from the 95% confidence intervals (CI) of the change, leveraging the known sample size, or calculated from the raw pre- and post-intervention means and SD (assuming an intercorrelation coefficient of 0.5 between the baseline and final measures). For multi-arm trials with a shared control group, we split the sample size of the control group by the number of active treatment arms to avoid double-counting participants. If outcomes were only shown in figures, WebPlotDigitizer 5.0 software was used to extract the corresponding data points. Between-study variance was estimated using the Restricted Maximum Likelihood (REML) method, and statistical heterogeneity was assessed using the Q statistic and I^2^. I² values were interpreted as follows: <30% low, 50% moderate, and above 75% high heterogeneity (Higgins & Thompson, 2002). Subgroup analyses of more than three studies were conducted based on type of exercise to explore potential sources of heterogeneity, and meta-regression analyses for more than 10 studies using REML were conducted to separately evaluate whether results were different by duration of intervention (weeks), frequency (sessions per week), mean age, BMI, and baseline values. Additionally, standardized residuals (Viechtbauer & Cheung, 2010) and leave-one-out analyses were used as complementary approaches to identify potential outliers and influential studies, respectively. Publication bias was assessed using funnel plots, Egger’s test for small-study effects, and a nonparametric trim-and-fill method to estimate the potential impact of missing studies on the pooled effect size. All analyses were conducted using Stata 17.0 (StataCorp, College Station, TX, USA).

Data reported in fewer than three studies, or that could not be synthesized as described above, were not included in the meta-analyses and were summarized narratively.

## Results

### Study selection

The database search identified 14,144 records, and 35 additional studies were found through manual searching of reference lists. After automatic and manual removal of duplicates, 9,910 records were screened by title and abstract. A total of 693 full-text articles were then assessed for eligibility and, finally, 94 articles were included in the qualitative synthesis analyses meeting the inclusion criteria. For synthesis purposes, 50 reports, which synthesize 43 studies, are included in the present review, having been selected based on the measurement of inflammatory parameters and oxidative stress markers. Thirty-nine studies were included in the various meta-analyses performed. The study flowchart is shown in Figure 1.

**Figure 1:**
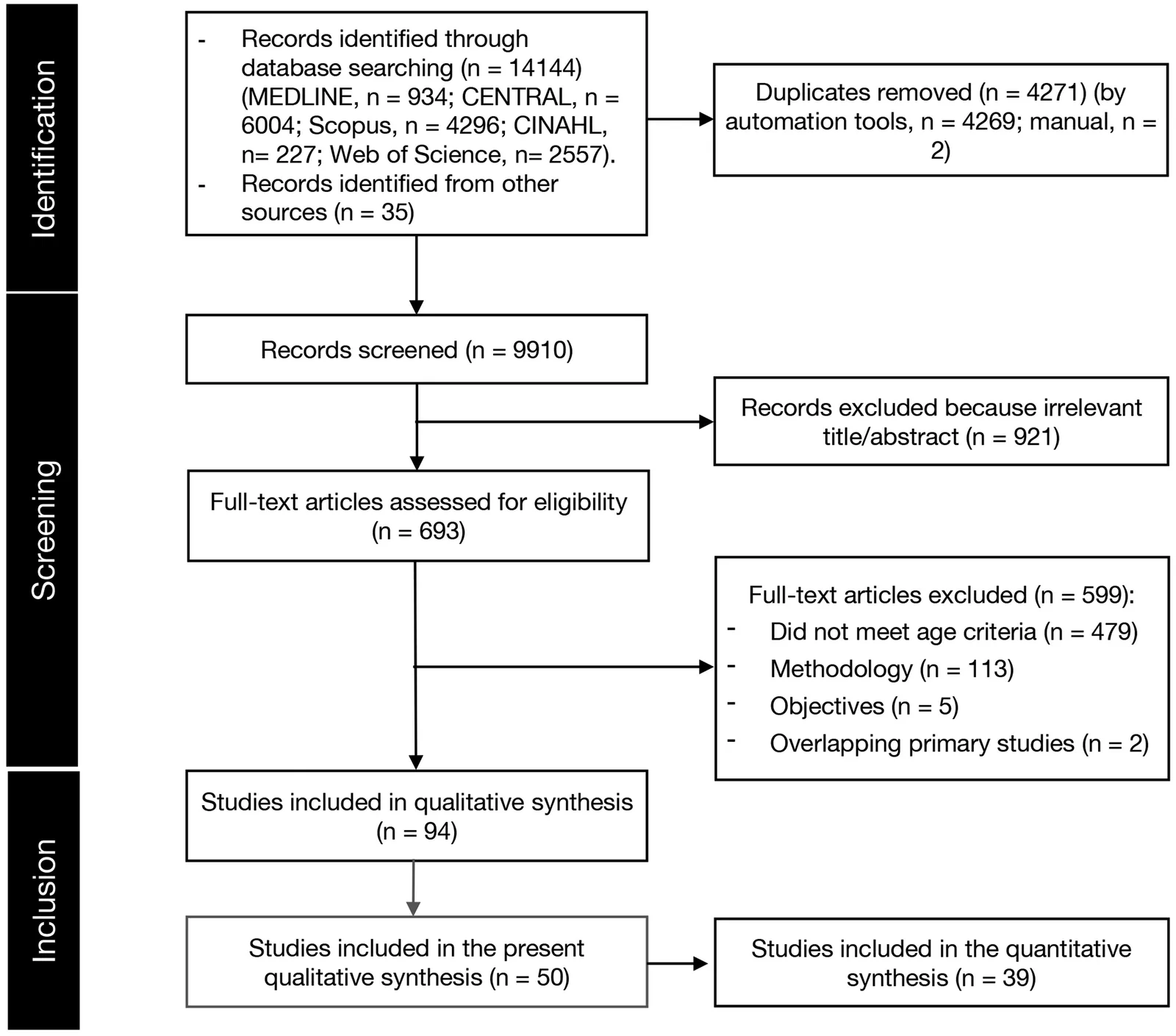
Flowchart of the study selection process.

### Study characteristics

Table 1 summarizes characteristics of participants, interventions, and results. A total of 4,379 participants were included in this review. The mean age of participants ranged from 66 to 92 years. Among the 24 and 16 studies reporting gender and sex distribution, respectively, 65.3% (1,668) of participants were women and 62.2% (949) were female. Five large studies were presented across 12 different reports. Overall, 43 studies evaluated the impact of physical exercise on inflammatory biomarkers, and eight assessed the effects on oxidative stress. All interventions involved supervised exercise, and three studies extended the initial supervised phase with a home-based program (Beavers et al., 2010; Custodero et al., 2023; Sadjapong et al., 2020). Intervention duration ranged from 8 weeks to 12 months, and training frequency varied between two and five sessions per week, with most studies implementing three sessions weekly. The reviewed studies primarily evaluated multicomponent training (22), followed by resistance training (16), including whole-body exercises or use of elastic bands or kettlebells, and aerobic protocols (5), mostly evaluating treadmills. These exercise interventions were typically compared against control groups that maintained their usual lifestyles or received basic health education, although one of them included flexibility exercises (Cao Dinh et al., 2019; Liberman et al., 2022; Salimans et al., 2024) and three included light stretching exercises (Beavers et al., 2010; Heo & Jee, 2024; Ruiz et al., 2014). Most studies excluded participants with major comorbidities; however, three included participants with sarcopenia (Bouchonville et al., 2014; Heo & Jee, 2024; Jung et al., 2022), one included participants living in care homes with pre-frailty or frailty and cognitive impairment (Furtado et al., 2020), six included participants with pre-frailty and/or frailty (Biesek et al., 2021; Caldo-Silva et al., 2021, 2023; Custodero et al., 2023; Sadjapong et al., 2020; Tan et al., 2023; Tarazona-Santabalbina et al., 2016), two included participants living in nursing-homes (Kapasi et al., 2003; Ruiz et al., 2014), two included participants with cognitive impairment (Abd El-Kader & Al-Jiffri, 2017; Phoemsapthawee et al., 2016), one included participants undergoing hemodialysis (de Luca Corrêa et al., 2022), and another one participants with rheumatoid arthritis (Kucharski et al., 2019).

**Table 1:** Summary of participants characteristics, interventions, and results.

Authors, year, country	*N* participants (control; intervention) (gender/sex)	Mean age ± standard deviation	Exclusion (E)/Inclusion (I) criteria	Intervention	Outcomes	Main findings (intervention effects)
**Inflammation**						
(Farinha, Santos, et al., 2022), Portugal	102 (20; continuous AT 25; interval AT 28; AT + RT 29) (78 W)	CG: 73.6 ± 5.3 IG: 71.4 ± 4.8; 72.6 ± 5.2; 71.9 ± 5.7	E: clinically diagnosed pathologies putting their and others’ health at risk while doing physical exercise in an aquatic environment; MMSE < 9 or clinically diagnosed with a mental illness; an attendance < 50% to sessions; failure to complete all proposed assessment tests	Aquatic exercises (continuous AT up to 70% HRmax; interval AT up to 80% HRmax; AT + RT up to 70% Maximum Heart Rate (HRmax), 3 sets, 16 repetitions, 6–7 Borg Scale), 28 weeks, 2 s/w	Monocyte Chemoattractant Protein-1 (MCP-1/CCL-2), macrophage inflammatory protein-1 alfa [macrophage inflammatory protein-1 alpha 1 (MIP-1α)/CCL3]	Continous AT: ↓ MCP-1, MIP-1α AT + RT: ↓ MCP-1
(Farinha, Ferreira, et al., 2022), Portugal					Interleukin (IL)-1Ra, IL-1ß, IL-10, tumor necrosis factor-alpha (TNF-α) Lymphocytes, leucocytes, monocytes and granulocytes	All intervention groups: ↓ TNF-α Interval AT: ↑IL-10, ↓ TNF-α /IL-10 AT+RT: ↓ IL-1ß/IL-1Ra Control group: ↓ IL-1Ra Interval AT and AT+RT : ↑ leucocytes and granulocytes Between-group : ↑ monocytes
Aerobic exercise						
(Muscari et al., 2010), Italy	109 (56; 53) (58 F)	CG: 69.6 ± 2.8 IG: 68.8 ± 2.5	E: cardiovascular diseases, MMSE score <24; 32 < body mass index (BMI) < 18; systolic blood pressure ≥ 180 or <110 mmHg; diastolic blood pressure ≥ 110 mmHg; resting heart rate <60/min; malignancy; moderate or severe respiratory insufficiency; severe arthrosis, recent fractures, palsy or relevant neuromotor deficits; hemoglobin <11 g/dl; use of beta-blockers, digitalis or other heart rate limiting drugs; any cardiac disease or of aortic aneurism >3.5 cm	Cycle ergometer, treadmill and free-body activity (70% HRmax), 12 months, 3 s/w	CRP	↓ CRP. Less pronounced decrease in MMSE.
(Abd El-Kader & Al-Jiffri 2016), Saudi Arabia	40 (20; 20) (29 F)	CG: 69.1 ± 6.1 IG: 68.9 ± 5.8	I: Alzheimer’s Disease E: smokers, pulmonary, cardiac, renal, hepatic, metabolic and neurological diseases	Treadmill (up to 70% HRmax), 2 months, 3 s/w	IL-6, TNF-α	↓ IL-6, TNFα (pre-post and between-groups). Improvement in psychological well-being and health-related quality of life
Resistance training						
(Flynn et al., 1999), USA	29 (14; 15) (All W)	CG: 72.9 ± 4.9 IG: 72.6 ± 3.5	E: dementia, arthritis, being bedridden within 3mo of the study, central or peripheral nervous system disorders, stroke, use of antidepressant medications, acute or chronic infection, major affective disorder, human immunodeficiency virus infection or autoimmune disorders, metabolic disorders, oral steroid use, cigarette or smokeless tobacco use, regular aerobic training or resistance training within previous 3 months, surgery within the previous 3 months, and caffeine consumption in excess of four cups of coffee per day	Up to 80% 1 Repetition Maximum (RM), 10 weeks, 3 s/w	Lymphocyte proliferation, natural killer cell activity, mononuclear cell populations	↑ NK and ↓ CD4+/CD8+ immediately postexercise compared with pre-exercise Improvement strength
(Phillips et al., 2010), USA	35 (7; 28) (All W)	CG: 73.1 ± 6.0 IG: 71.1 ± 6.2	NI	Whole-body exercises (three sets, 12 repetitions, up to 8 RM), 10 weeks, 3 s/w	IL-1ß, IL-6, TNFα (plasma and LPS-stimulated production) Leukocytes	Lower increase of IL-6, TNFα, Lipopolysaccharide (LPS)-IL1ß, LPS–IL-6, LPS- TNFα immediately after exercise ↑ Leukocyte count immediately after and 2h post-exercise Improvement strength
(So et al., 2013), Korea	45 (22; 13) (27 F)	CG: 68.4 ± 5.8 IG: 71.6 ± 5.5	I: not regular exercise, no orthopedic problems, regular attendance at the senior welfare center	Elastic band exercise program, 12 months, 3 s/w	CRP, IL-1ß, IL-6, TNFα	No changes. Improvement in body composition and fitness factors (chair stands, arm-curl, 2-min step, chair sit-and-reach and back scratch)
(Rodriguez-Miguelez et al., 2014), Spain	26 (10; 16) (19 F)	CG: 70.0 ± 0.9 IG: 69.1 ± 1.1	E: any medication known to affect the inflammatory response 1 month before or during the study; hormonal treatment	Cycle ergometer + leg press, biceps curl and pec deck (up to 80% 1 RM), 8 weeks, 2 s/w	PBMCs total RNA PBMCs: Hsp60, Hsp70, Toll-Like Receptor (TLR)2, TLR4, TRIF, IKKi/IKKε, forMyD88, p65, IRF3, phospho-IRF3, IRF7, phospho-IRF7, extracellular signal-regulated kinases 1 and 2 [Extracellular Signal-Regulated KinaseERK1/2], phospho-ERK1/2, p38, phospho-p38 and β-actin, TNF-α, IL-10 Plasma CRP and IL-6	PBMCs: modulation of inflammatory pathways. Plasma: ↓ CRP, IL-6 Improvement in strength
(Mejías-Peña et al., 2017), Spain	26 (10; 16) (19 F)	CG: 69.2 ± 1 IG: 70.2 ± 0.8			Autophagy: LC3II/LC3I, p62/SQSTM1, Atg12, Atg16, phospho-ULK-1 [ser757], beclin-1, lysosomal LAMP-2) Inflammation: NLRP3, pro-caspase-1, caspase-1 Apoptosis: Bcl-2, Bcl-xL, Bad, caspase-3	Pre-post and between-group comparisons: ↓ p62/SQSTM1, phospho-ULK-1 (ser757], lysosomal LAMP-2. NLRP3, caspase-1/procaspase-1, Bcl-2, Bcl-xL, Bad, caspase-3, ↑ Atg12, Atg16, beclin-1 Improvement voluntary isometric contraction and strength.
Strandberg et al., 2015), Sweden	55 (18; 17; [20 training + diet]) (All W)	CG: 68 ± 1 IG: 68 ± 2	E: self-reported inability to walk; cardiovascular, pulmonary, metabolic, rheumatologic, and psychiatric disease; musculoskeletal problems; use of medication; a food allergy; unexplained weight loss	Squat, leg extension, leg press, seated row, and pull down (three sets of 12–15 repetitions, up to 85% of 1 RM), 24 weeks, 2 s/w	CRP, IL-6, arachindonic acid (AA)	Improvement in muscle strength
(Hsieh et al., 2018), Taiwan	30 (15; 15) (19 W)	CG: 71.8 ± 4.5 IG: 70.6 ± 4.2	E: HbA1c (glycosylated hemoglobin) levels 10% or more, cancer, cognitive dysfunction, or any clinical diagnosis of cardiopulmonary, musculoskeletal, and neurological diseases that might influence exercise performance	Chest press, shoulder press, biceps curl, hip abduction, standing hip flexion, leg press, standing calf raise, and abdominal crunch (three sets, 8–12 repetitions, up to 75% 1 RM or 14-16 Borg scale), 12 weeks, 3 s/w	hs-CRP	↓ hs-CRP. Improvement in muscle function and physical performance
(Chen et al., 2018), Taiwan	33 (16; 17) (All W)	CG: 66.7 ± 5.3 IG: 68.3 ± 2.8	I: sarcopenia. E: immediate disease(s), signs of infection, vaccinated within the past 4 weeks, engaging in excessive alcohol or drug consumption, currently receiving cancer treatment, receiving chronic disease medication on a long-term basis, and currently receiving medication that affects the immune system	Kettlebell weight training (3 sets, 8-12 repetitions, up to 70% of 1-RM), 8 weeks, 2 s/w. Detraining period of 4 weeks	hs-CRP, IL-6, TNFα,	↓ hs-CRP (8-w and 12-w). Improvement in body composition, strength and pulmonary function
(Ihalainen et al., 2019), Finland	94 (20; 1 s/w 24; 2 s/w 24; 3 s/w 26) (50 W)	CG: 69.4 ± 2.2 IG: 69.8 ± 2.9; 68.8 ± 2.9; 69.5 ± 2.2	E: regular aerobic exercise, any previous strength training experience, BMI >37, serious cardiovascular disease or lower limb injuries/disease, use of walking aids, use of medication that affect the neuromuscular, endocrine systems or affect the variables within the study, previous testosterone-altering treatment, smoking	Whole-body strength training (up to 80% 1 RM), 6 months	hs-CRP, IL-6	No changes. Improvement in body composition (3 s/w)
(Urzi et al., 2019), Slovenia	20 (9; 11) (All W)	CG: 88.9 ± 5.3 IG: 84.4 ± 7.7	I: free of physical impairment, not pacemaker, terminal illness, severe dementia or knee or hip arthroplasty	Elastic resistance band-based training at moderate intensity (Borg Rate of Perceived Exertion scale level 12–14), 12 weeks, 3 s/w	CRP, IL-8, IL-15, resistin	No changes. Improvement in physical function [Short Physical Performance Battery SPPB, gait speed, chair rise]
(Cao Dinh et al., 2019), Belgium	57 (20; strength endurance 16; intensive strength 21) (All W)	NI	E: immunosuppressive or anti-inflammatory medication, unable to understand or execute the exercise instructions due to cognitive impairment (MMSE < 24), performing regular physical exercise at higher intensities currently or within the past 6 months. uncontrolled conditions, acute inflammation (CRP > 10 mg/L)	Strength endurance (two sets, 30 repetitions, 40% 1 RM), intensive strength (three sets, 10 repetitions, 80% 1 RM); 6 weeks, 3 s/w CG: flexibility training	Circulating senescence-prone T-cells	Endurance training in CMV seropositive group: ↓ senescent phenotypes (CD8 + CD28–CD57+, CD8–CD28–CD57+, CD8–CD28 + CD57+), ↑ Memory (CD8 + CD28 − CD57−), naïve (CD8-CD28 + CD57-)
(Liberman et al., 2022), Belgium	14 (5; Endurance 5; RT 4) (All W)	CG: 64.2 ± 4.6 IG: 69.4 ± 12.5; 62.6 ± 5.4		Strength endurance (two sets, 30 repetitions, 40% 1 RM), intensive strength (three sets, 10 repetitions, 80% 1 RM); 3 weeks, 3 s/w CG: flexibility training	PBMC expression of inflammation-related genes	Change of 98 genes, 33 pathways. Changes on CLIP-related genes.
(Salimans et al., 2024), Belgium					LPS-stimulated PBMC gene expression	Change of 151 genes, 29 pathways.
(Biesek et al., 2021), Brazil	90 (18; 18; [18 protein supplementation; 18 exergames + protein; 18 exergames + isoenergic supplementation]) (All W)	CG: 70.4 ± 3.9 IG: 71.2 ± 4.2	I: pre-frail (Fraily phenotype), moderate kidney functioning, compensated type II diabetes if presented. E: acute or terminal illness, metabolic instability or decompensated cardiovascular disease, cognitive deficits, neurological disorders and/or traumatic-orthopedic conditions, type I diabetes, medications that might affect muscle metabolism oor postural balance, cardiac, respiratory or hepatic deficiency and/or decompensated arterial hypertension	Exergames (Nintendo Wii Fit Plus®) : neuromotor exercises and resistance exercises	IL-6	No changes
(Timon et al., 2021), Spain	61 (20; 21; [20 RT in hypoxia]) (32/54 W)	CG: 70.5 ± 4 IG: 70.3 ± 3.3	I: not receiving medication that affects the immune system (steroidal and non-steroidal anti-inflammatory, antidepressants), cancer o arthritis treatment	Whole-body exercises (band, kettlebells) (3 sets, 10-15 repetitions), 24 weeks, 3 s/w	CRP, IL-8, IL-6, IL-10	↓ CRP, IL-8, IL-6/IL-10 Between-group comparison: ↓ CRP, IL-8
(de Luca Corrêa et al., 2022), Brazil	25 (12; 13) (6 F)	68.3 ± 1.1	I: undergoing chronic hemodialysis in the previous six months; no complications from unstable metabolic medical diseases or cardiovascular complications; no orthopedic problems that prevented execution of assessments and training; and blood pressure <180/100 mmHg. E: severe decompensated metabolic disorders; habitual exercise patterns; stroke in the previous six months; dialysis adequacy (Kt/V) < 1.2; history of access to arteriovenous fistula in the previous three months; and/or presence of autoimmune disease(s)	Whole-body exercises (three sets, 8–12 repetitions), 24 weeks, 3 s/w	IL-6, IL-10, TNF-α Nitrite/nitrate plasma concentration (NO^-^)	↓ IL-6 (between-group comparison), TNF-α (pre-post and between-group comparison) Group × time interaction: NO^-^
(de Sá Souza et al., 2022), Brazil	28 (14; 14) (18 F)	CG: 74.6 ± 7.1 IG: 77.4 ± 6.3	E: history of current smoking, frequent alcohol consumption, use psychoactive drugs, sedentary, hypertension, diabetes or hypercholesterolemia not controlled by drugs, heart failure, participation in a physical training program in the last year	Whole-body exercises (up to three sets, 8 repetitions, 75% 1 RM), 12 weeks, 3 s/w	IL-1Ra, IL-6, IL-10, TNFα	Between-group comparisons: Men: ↓ TNFα. ↑ IL-1Ra. ↑ IL-10
(Heo & Jee, 2024), Korea	88 (22; Low-intensity strength 22; Moderate-intensity strenght 22; High-intensity strenght 22) (39 F)	CG: 67.3 ± 3.5 IG: 67.8 ± 4.9; 67.5 ± 4.7; 68.0 ± 5.1	I: sarcopenia	Whole body exercises (3 sets; low intensity [LI]: 12-11-10 RM; moderate [MI] 10-9-8 RM; high [HI] 8-7-6 RM), 12 weeks, 3 s/w	IL-6, IL-10, TNFα. Leukocytes. Distribution and quantity of T cells, NK cells	↓ IL-6 (MI, HI), TNFα (HI) ↑ IL-10 (MI) In % : ↑ NK cells, CD3 T cells, CD4 T cells, CD8 T Cells, CD19 B cells (↓ all in CG) Improvement body composition
Multicomponent exercise						
(Kapasi et al., 2003), USA	190 (96; 94) (159 F)	CG: 88 ± 7 IG: 87 ± 8	I: living in nursing homes E: in postacute skilled care units or terminally ill; incontinent of urine, with a catheter, unable to follow a 1-step instruction	Endurance (standing, walking and wheelchair endurance), upper body strength training, 8 months, 5 s/w	Lymphocyte enumeration, characterization and proliferation Plasma neopterin and sTNF-RII	↑ sTNF-RII. Improvement exercise tolerance.
(Beavers et al., 2010), USA	368 (186; 182) (251 F)	CG: 77 ± 4.4 IG: 76.4 ± 4.1	I: SPPB <10, sedentary lifestyle, ability to complete a 400-meter walk test within 15 minutes without sitting and without using an assistive device E: self-reported inability to walk one mile; significant cognitive impairment (MMSE Score < 21); severe hearing or visual impairment; and severe cardiac, pulmonary, neurological, orthopedic, renal, or psychiatric disease	Aerobic, strength, balance and flexibility exercises, 12 months (initially center-based, later home-based), 2–3 s/w Control group: health education meetings	CRP, IL-6, IL6sR, IL-1sRII, IL-8, IL-15, IL-1sRII, sTNFRI, sTNFRII, IL-1Ra, IL-2sRα, TNF-α	More ↓ than the control: IL-6, IL-8, Soluble Tumor Necrosis Factor Receptor I (sTNFRI) Linear regression models (adjusted): ↓ IL-8, ↓ IL-15 in participants with baseline IL-15 above the median
(Martins et al., 2010), Portugal	63	76 ± 8	I: sedentary. E: impaired glucose tolerance and diabetes, physical or psychological diseases that may preclude ability to perform training exercises and testing, medications know to influence physical performance or interpretation of the findings	Aerobic (up to 85% HRmax) and strength-based training with elastic bands, 16 weeks, 3 s/w	hs-CRP	↓ hs-CRP. Improvement in body composition
(Kwon et al., 2012), Korea	30 (15; 15) (All W)	CG: 73.6 ± 2.2 IG: 74.0 ± 2.9	I: sedentary, essential hypertension stage 1-2 E: secondary hypertension, end organ damage, left ventricular hypertrophy, retinal changes, microalbuminuria, ≥3 cardiovascular risk factors, ≥1 antihypertensive drug	Flexibility, muscle strength training, weight bearing exercise (60-70% max. HR), yoga, 24 weeks, 4s/w	IL-6, TNF-α	↓ IL-6 and TNF-α Improvement on body composition
(Bouchonville et al., 2014), USA	107 (27; 26; [26 diet; 28 diet + exercise]) (34 F)	CG: 69 ± 4 IG: 70 ± 4	I: BMI ≥ 30 kg/m^2^, stability of body weight within 2 kilograms over the preceding year, sedentary lifestyle, on a stable medications for at least 6 months prior to enrollment, mild-to-moderate frailty E: severe cardiopulmonary disease, musculoskeletal/neuromuscular impairments, significant cognitive impairment, a history of malignancy, current smoking, history of diabetes or fasting glucose of ≥126 mg/dl	Flexibility, aerobic exercise (up to 85% HRmax), resistance training (up to 1–2 sets, 8–12 repetitions, 85% 1 RM), 12 months, 3 s/w CG: general information regarding a healthy diet at monthly visits	High-Sensitivity C-Reactive Protein (hs-CRP), soluble tumor necrosis factor receptor 1 (sTNF R1)	No changes in the training group alone. Improvement in body composition
(Ko et al., 2014), Korea	54 (15; 1 day 15; 2–3 days 11; 5 days 13) (All W)	CG: 70.5 ± 5.9 IG: 72.1 ± 4.7; 73.4 ± 0.9; 76.1 ± 7.0	I: independent in the Barthel index and Performance-Oriented Mobility Assessment (POMA) index E: history of cancer, or being current or former smokers; history of chronic liver disease or abnormal liver function; history of chronic renal disease, or abnormal kidney function	Cycling, treadmill walking; and weight bearing (up to 60% 1 RM, up to 15 repetitions, two sets), 12 weeks	Lymphocytes	No changes. Improvement in weight
(Ruiz et al., 2015), Spain	40 (20; 20) (32 W)	CG: 92.1 ± 2.3 IG: 92.3 ± 2.3	I: nursing home, able to ambulate (with or without the assistance), able to communicate, not participating in any other trial E: acute or terminal illness, myocardial infarction in the past 3 months, unstable cardiovascular disease or other medical conditions, upper or lower extremity fracture in the past 3 months, severe dementia, neuromuscular disease, using drugs affecting neuromuscular function	Cycle-ergometer + strength training of major lower limb muscles (2-3 sets of 8-10 repetitions, up to 70% of 1 RM), 8 weeks, 3 s/w. Detraining period of 4 weeks	TNF-α	No changes after the intervention or the detraining period. Improvement in muscle leg strength
(Jin et al., 2015), Korea	16 (8; 8) (All W)	CG: 76.1 ± 2.0 IG: 74.3 ± 0.6	I: impaired fasting glucose (≥ 100mg/dl), any medical diseases or exercise habit	Flexion/extension, abduction/adduction, knee extension, leg press, 12 weeks, 2s/w + 3s/w at home	IL-6	↑ IL-6. Improvement SPPB and grip strength
(Lim et al., 2015), Korea	20 (10; 10) (All W)	CG: 71.3 ± 1.7 IG: 70.7 ± 2.2	E: lipid- and/or glucose-lowering medication, cardiovascular disease, percentage of body fat < 30%	Aerobic exercises (i.e. walking and arm raise), resistance training (i.e. arms fold and spread), traditional Korean dance (up to 60-70% max. HR), 12 weeks, 3s/w	CRP	No changes
(Kim et al., 2016), Japan	139 (34; 35; [36 Ex + Nut; 34 Nut]) (All W)	CG: 81.1 ± 5.1 IG: 81.4 ± 4.3	I: severely impaired mobility; MMSE < 24; missing baseline data; unstable cardiac conditions or other musculoskeletal conditions	Stationary bicycle + chair exercises, resistance band exercise and hydraulic machine, 3 months, 2 s/w CG: general health education class (once every 2 weeks)	hs-CRP, IL-6	No changes. Improvement in body composition and muscle strength
(Kim et al., 2018), South Korea	47 (15; RT 16; MT 16) (32/35 F)	73.2 ± 4.9	I: no significant illness within the last 6 months, those who had not exercised regularly in 3 months before the participation	RT: six pieces of outdoor exercise equipment. MT: RT + sky-walk and cross-country 12 weeks, 3 s/w	IL-6	No changes. Improvement in physical function and muscular strength
(Kucharski et al., 2019), Sweden	74 (38; 36) (56 W)	CG: 70.1 ± 3.1 IG: 69.1 ± 2.6	I: rheumatoid arthritis (≥ 2 years, disease activity score 28 < 5.1). E: unstable ischemic heart disease or arrhythmia, joint surgery within the 6 months prior to inclusion, ongoing exercise, inability to understand or speak Swedish, inability to participate in physical testing	Aerobic and resistance exercises, 20 weeks, 3 s/w	CRP, erythrocyte sedimentation rate (ESR)	No changes. Improvement In fatigue, activity and depression
(Furtado et al., 2020), Portugal	60 (19; MC 21; RT 20)	CG: 80.9 ± 10.0 IG: 80.1 ± 8.2; 81.0 ± 4.8	E: not completing the dynamic balance test in 30 seconds; an involvement in other exercise programs; not having controlled and updated drug therapy; any type of health condition that could prevent testing of autonomy; medication that could significantly impair attention and disturb psychomotor activity; morbid obesity; mental, hearing, and vision impairment; use of hormone therapy	MC: chair exercises (2-4 sets, 8-16 repetitions, up to 75% HRmax); RT: chair muscle strength exercises (2-3 sets, 10-15 repetitions, up to 80% HRmax), 28 weeks, 3 s/w	CRP, IFN-γ, IL-1β, IL-6, IL-10, TNF-α	Interaction of time by group with ↑ IL-10 Moderate ↓ TNF-α/IL-10 (AT) Improvement in physical fitness
(Sadjapong et al., 2020), Thailand	64 (32; 32) (39 F)	CG: 88.8 ± 7.2 GI: 78.9 ± 1.3	I: Frail (Fried’s frailty phenotype) E: progressive degenerative diseases, MMST10 ≤ 23, severe audiovisual impairment, severe disability	Chair aerobic training (up to 65% HRmax), resistance training with theraband (up to 3 sets, 12 repetitions, 90% 1 RM), balance training, 12 weeks (supervised) + 12 weeks (home-based), 3 s/w	CRP, IL-6	↓ CRP, IL-6 (both interaction group x time). Improvement in physical performance, frailty and quality of life
(Caldo-Silva et al., 2021), Portugal	35 (13; 7; [8 training + Branched-chain amino acids (BCAAs); 7 BCAAs])	CG: 83.1 ± 5.4 IG: 86.7 ± 4	I: physically frail and pre-frail; clinically stable; being able to perform the Time Up and Go test in ≤ 50 s; not participating in other structured regular physical exercise; not presenting any type of health condition or use medication that might prevent the functional self-sufficiency test performance or attention impairment; not presenting mental disorders or hearing/visual impairment that could prevent the evaluations and activities proposed; BMI ≤40	Elastic-band exercises, walkability program, step exercises, coordination, and balance, 16 weeks (8 weeks wash-out + retraining 16 weeks), 2 s/w	IL-10, TNF-α MPO	No changes in the evaluated groups.
(Caldo-Silva et al., 2023), Portugal					Lymphocytes, monocytes, granulocytes	No changes.
(Jung et al., 2022), Korea	30 (15; 15) (All W)	CG: 74.6 ± 5.8 IG: 75.4 ± 4.5	I: sarcopenia comorbid with obesity E: exercises over the last 6 months, chronic diseases; retinal laser treatment; history of acute myocardial infarction; joint replacement or fracture of the lower limb six months prior, severe cognitive disturbance	Circuit training (10 movements, up to 80% HR reserve), 12 weeks, 3 s/w	hs-CRP, IL-6	↓ IL-6, Improvement in body composition
(Custodero et al., 2023), USA	1635 (817; 818) (861 W)	CG: 79.1 ± 5.3 IG: 78.6 ± 5.2	I: sedentary lifestyle, SPPB ≤ 9, ability to walk 400 m in less than 15 min without sitting, leaning or the assistance of another person E: nursing homes, unable to communicate, medical conditions (cognitive impairment, arthritis awaiting joint replacement, severe cardiovascular diseases, respiratory disease, regular corticosteroids or oxygen, active cancer, end-stage renal disease in dialysis, psychiatric disease, neurodegenerative disorders, terminal illnesses, other physical complaints.	Walking, strength (primarily lower extremity), balance and flexibility exercises (initially center-based 2 s/w, later home-based 3–4 s/w), 12 months	IL-6	No changes. Inverse correlation between difference in 400 m gait speed and difference in IL-6
(Scano et al., 2023), Italy	120 (60; 60)	NI	E: BMI > 35, the presence of diseases that preclude moderate physical activity and participation in a physical activity program	Aerobic and anaerobic exercises (up to 60% HR reserve), 12 weeks, 3 s/w CG: cultural and recreational activities around the city, supported by a health educator	CRP, ESR	No changes
(Tan et al., 2023), Singapore	324 (187; 80; [MT + cognitive trainig 57]) (138 F)	CG : 71.7 ± 5.0 IG : 73.4 ± 5.2	I: pre-frail based on FRAIL questionnaire. E: pacemaker or a defibrillator, cardiac, liver, gastro-intestinal, or end stage lung disease, cancer undergoing active treatment, gout, underlying psychiatric conditions	Aerobic, resistance, dual task and balance training, 6 months, 2 s/w (6 months wash-out period). Control group: general health education advice	IL-6, IL-10, TNF-α, GDF-15	↓ TNF-α at 3 months. Improvement in gait speed, body composition, GDS.
**Oxidative stress**						
Aerobic exercise						
(Fatouros et al., 2004), Greece	19 (8; 11) (All M)	CG: 71.5 ± 6.5 IG: 72.8 ± 4.8	I: inactive before the study; free of musculoskeletal problems and potentially orthopedic/neuromuscular limitations; resting blood pressure below 160/100 mmHg; no signs of cardiovascular/respiratory complications; no tobacco use within the 6 months before the study; had normal dietary habits, did not consume aspirin or alcoholic beverages at least 1 week before exercise testing; not consuming antioxidant compounds	Walking/jogging on a treadmill (50%–80% HRmax), 16 weeks, 3 s/w	Malondialdehyde (MDA) and 3-nitrotyrosine (3-NT) levels. Total antioxidant capacity (TAC), glutathione peroxidase (GPX)	↓ MDA and 3-NT ↑ TAC and GPX Improvement in body composition
(Alghadir et al., 2016), Saudi Arabia	100 (50; 50) (35 F)	CG: 69.7 ± 5.91 IG: 67.3 ± 2.8	E: physical disability, endocrine, immune, and psychiatric Illness, eating disorders and taking glucocorticoids	Treadmills, bicycles and StairMasters (up to 70% HRmax), 24 weeks, 3 s/w	hs-CRP, TAC, MDA, 8-hydroxyguanine (8-OHdG)	Pre-post comparisons: ↓ hs-CRP Higher ↓ MDA, 8-OHdG Higher ↑ TAC Improvement cognitive abilities
(Phoemsapthawee et al., 2016), Thailand	24 (12; 12) (All W)	65-87	I: MMSE ≤ 23. E: significant cardiovascular and/or cerebrovascular disease, uncontrolled hypertension and diabetes, musculoskeletal impairment, other medical conditions with significant psychiatric or neurologic consequence	Arm swing exercise, 12 weeks, 5 s/w (cross-over: wash-out period 12 weeks, training 12 weeks)	8-iso-PGF2α	Pre-post and between-group comparisons: ↓8-iso-PGF2α
Resistance training						
(Beltran Valls et al., 2014), Italy	23 (10; 13) (11 F)	CG: 72 ± 1 IG: 72 ± 1	E: cardiovascular, metabolic, and pulmonary disease; orthopedic injury or joint disease; neurological or immunologic disease or involved in resistance training into the past 12 months	Explosive-type resistance training (up to 3–4 sets of 12 repetitions), 12 weeks, 2 s/w	IL-4, IL-6, IL-13, IL-15, TNFα, macrophage inflammatory protein-1-beta (MIP-1b). Myeloperoxidase (MPO). PMBCs: Hsp27, thioredoxin system (TrxR1, Trx1, Trx2, TrxR2), ß-actin, Hsp70, 4-hydroxynonenal (HNE)	↓ MPO, interaction time x group. ↓ Hsp70 and Hsp27 proteins. ↓ TrxR1. Increase body power, strength, and functional motor performance
(Ceci et al., 2014), Italy	16 (8; 8)	CG: 74 ± 2 IG: 71 ± 2			Glutathione (GSH), protein carbonyls, MDA. DNA damage and apoptosis	Higher GSH/GSSG (glutathione disulphide) ratio immediately after the exercise. Less pronounced increase in MDA levels 1h after the training. Lower value of protein carbonyls 24-after the exercise.
(Dimauro et al., 2016), Italy	20 (10; 10) (10 F)	CG: 72 ± 1 IG: 72 ± 1			Relative telomere length (TL), Shelterin protein TRF2 and Ku80, DNA methylation PBMCs: MnSOD, SIRT1, SIRT2, total protein acetylation, Bcl-2, Bax, Caspase 3	↑ TL (positive correlation: functional power, strength test; negative correlation: 6-min walking speed, 6-m walking loaded, loaded stairs climbing) ↓MnSOD expression ↓ Global DNA methylation level (pre-post and between-group comparisons)
Multicomponent exercise						
(Son et al., 2017), Korea	20 (10; 10) (All W)	CG: 74.7 ± 2 IG: 76 ± 5	I: stage 1 hypertension, menopause, sedentary E: BMI ≥ 25 kg/m^2^, smokers, psychiatric conditions, medications or homone therapy in the year before	Resistance band exercises and walking (up to 70% HR reserve), 12 weeks, 5 s/w	Plasma nitrite/nitrate	↑ nitrite/nitrate (↓ CG). Improvement cardiovascular risk factors
(Tarazona-Santabalbina et al., 2016), Spain	100 (49; 51) (54 F)	CG: 80.3 ± 3.7 IG: 79.7 ± 3.6	I: sedentary, frail according to frailty phenotype, walking speed <0.8m/s E: life expectancy less than 6 months, MMSE <24, severe disability (<15 points on the Barthel Scale or an E score or higher in the Katz scale), ejection fraction left ventricle 20% or less, hospital admission in the past 3 months, oncologic patient with active treatment, major non-ambulatory surgery in the past 6 months, family member centenarian in the previous 2 generations, patient with a coronary event in the past 12 months	Endurance (up to 65% HRmax), strength (up to 75% 1 RM), coordination, balance, and flexibility exercises. Both groups received nutritional recommendations and vitamin D supplementation if needed	Protein carbonylation	Lower ↑ plasma protein carbonyls. Improvement physical performance, frailty, cognitive, emotional and social parameters

*Note*. Results report pre-post changes unless stated differently. Discontinued lines represent results from the same studies. AT = aerobic training; CG = control group; CRP = C-Reactive Protein; F = female; GDS = Global Deterioration Scale; hs-CRP = high-sensitivity CRP; IFN = interferon; IG = intervention group; IL = interleukin; LPS = lipopolysaccharide; M = male; MMSE = Mini-Mental State Examination; NI = no information; PBMC = peripheral blood mononuclear cells; RT = resistance training; s/w = sessions per week; SPPB = Short Physical Performance Baterry; sTNF-RII = soluble Tumor Necrosis Factor Recepto II; TNF = tumor necrosis factor; W = women. Control group did not receive any treatment unless stated differently.

### Assessment of inflammation status

#### C-reactive protein and high-sensitivity C-reactive protein

A total of 12 studies reported C-reactive protein (CRP) (Beavers et al., 2010; Furtado et al., 2020; Kucharski et al., 2019; Lim et al., 2015; Muscari et al., 2010; Rodriguez-Miguelez et al., 2014; Sadjapong et al., 2020; Scano et al., 2023; So et al., 2013; Strandberg et al., 2015; Timon et al., 2021; Urzi et al., 2019) while eight reported and high-sensitivity C-reactive protein (hsCRP) (Bouchonville et al., 2014; Chen et al., 2018; Hsieh et al., 2018; Ihalainen et al., 2019; Jung et al., 2022; Kim et al., 2016; Martins et al., 2010).

Meta-analysis of 11 studies (12 comparisons) reporting CRP and seven studies (nine comparisons) reporting hsCRP resulted in a pooled effect size of Hedges’ g = −0.54 [95% confidence interval (CI): −1.04 to −0.05], with high heterogeneity (I^2^ = 91.6%) for CRP, and Hedges’ g = −0.41 (95% CI: −0.80 to −0.02), with moderate heterogeneity (I^2^ = 70.7%) for hsCRP (Figures 2 and 3, respectively). Subgroup analyses only revealed significant effects of multicomponent exercise on CRP (Hedges’ g = −0.27, 95% CI: −0.50 to −0.04) with low heterogeneity (I^2^ = 17.3%) (Figure 4), while no effects were found on high-sensitivity C-Reactive Protein (hs-CRP) (Figure S1). However, the test for subgroup differences revealed no significant interaction between these exercise types in CRP [Q_b_(2) = 2.39, *p* = 0.30] or hs-CRP [Q_b_(1) = 0.52, *p* = 0.47]. Meta-regression analysis did not show any significant association between changes in CRP levels and the selected variables.

**Figure 2:**
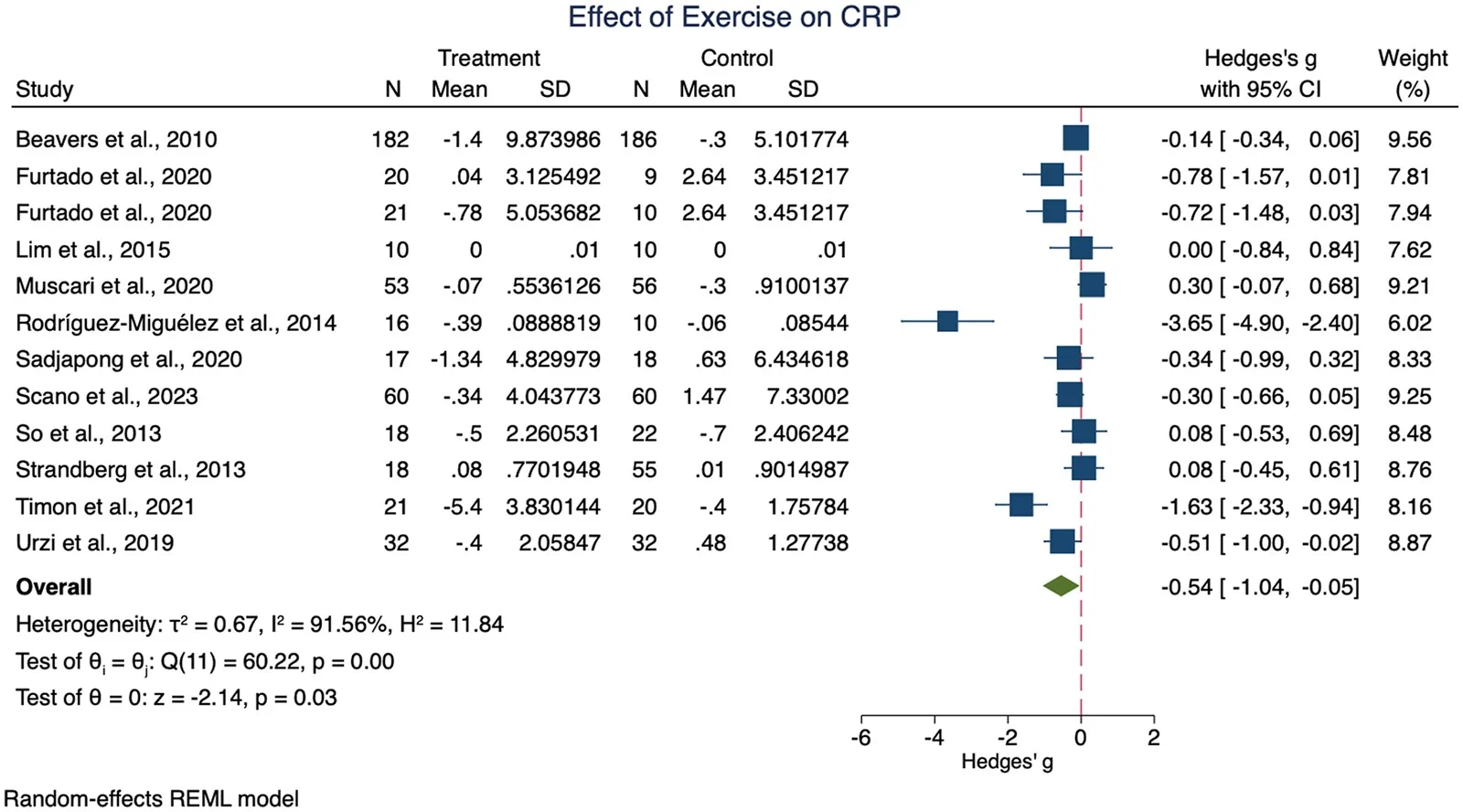
Meta-analysis of studies reporting C-reactive protein. *Note.* CRP = C-Reactive Protein; REML = Restricted Maximum Likelihood.

**Figure 3:**
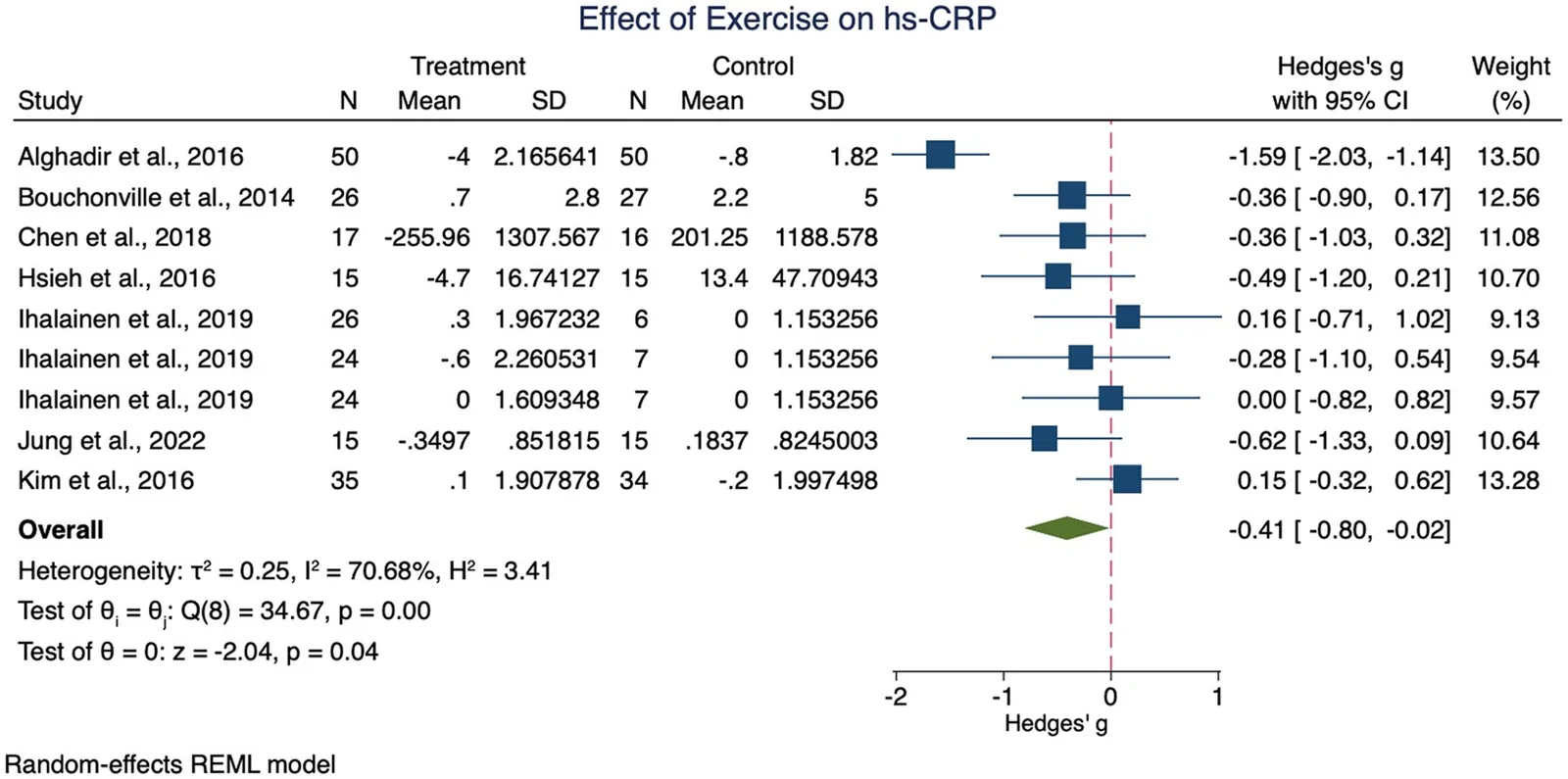
Meta-analysis of studies reporting high-sensitivity C-reactive protein. *Note.* CRP = C-Reactive Protein; REML = Restricted Maximum Likelihood.

**Figure 4:**
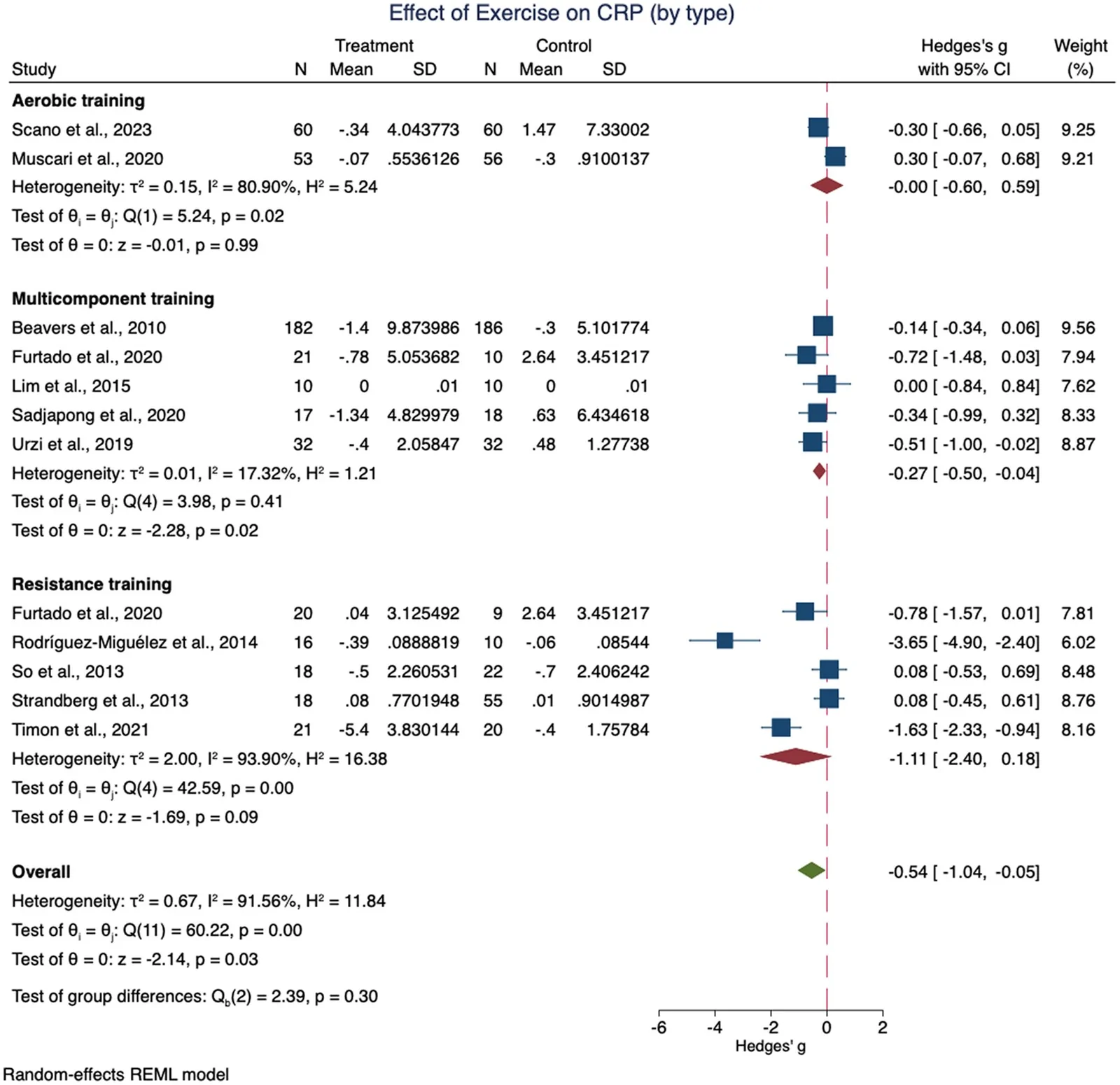
Subgroup analyses of C-reactive protein according to exercise type. *Note.* CRP = C-Reactive Protein; REML = Restricted Maximum Likelihood.

#### Interleukin-1ß

None of the four studies evaluating interleukin-1ß (IL-1ß) found significant changes (Farinha, Ferreira, et al., 2022; Furtado et al., 2020; Phillips et al., 2010; So et al., 2013); only one multicomponent exercise reduced the IL-1ß/IL-1Ra ratio (Farinha, Santos, et al., 2022). Meta-analysis of all studies (seven comparisons) did not draw significant effects (Hedges’ g = 0.06, 95% CI: −0.38 to 0.49) (Figure S2).

#### Interleukin-1Ra

Three studies evaluated interleukin-1Ra (IL-1Ra): aquatic aerobic training provoked a significant decrease in the control group with a decrease in IL-1ß/IL-1Ra ratio in the multicomponent intervention and no changes in the aerobic intervention (Farinha, Santos, et al., 2022); while a resistance training intervention only reported a significant difference between-groups after the intervention period, with a higher increase in the intervention group (de Sá Souza et al., 2022), and a multicomponent intervention did not change the levels (Beavers et al., 2010). Meta-analysis of all studies (five comparisons) did not show any significant effect (Hedges’ g = 0.00, 95% CI: −0.18 to 0.17) (Figure S3).

#### Interleukin-6

Twenty-two studies reported interleukin-6 (IL-6) (Abd El-Kader and Al-Jiffri 2016; Beavers et al., 2010; Beltran Valls et al., 2014; Biesek et al., 2021; Chen et al., 2018; Custodero et al., 2023; de Luca Corrêa et al., 2022; de Sá Souza et al., 2022; Furtado et al., 2020; Heo & Jee, 2024; Ihalainen et al., 2019; Jin et al., 2015; Jung et al., 2022; Kim et al., 2016; Kwon et al., 2012; Phillips et al., 2010; Rodriguez-Miguelez et al., 2014; Sadjapong et al., 2020; So et al., 2013; Strandberg et al., 2015; Tan et al., 2023; Timon et al., 2021).

One study evaluating IL-6 levels immediately after a bout of exercise in women (Phillips et al., 2010) reported that IL-6 increased acutely, but this increase was attenuated after the intervention period. Moreover, one study found a reduced IL-6/IL-10 ratio (Timon et al., 2021) and another saw an inverse correlation between differences in 400 m gait speed and differences in IL-6 (Custodero et al., 2023).

Meta-analysis of 20 studies (26 comparisons) resulted in a pooled effect size of Hedges’ g = −0.27 (95% CI: −0.75 to 0.21), with high heterogeneity (I^2^ = 92.7%) (Figure 5). Subgroup analyses only revealed significant effects of multicomponent exercise on IL-6 (Hedges’ g = −0.18, 95% CI: −0.34 to −0.02) with low heterogeneity (I^2^ = 0%) (Figure 6). The test for subgroup differences revealed no significant interaction between these exercise types [Q_b_(2) = 3.54, *p* = 0.17]. Meta-regression analysis only showed a significant association between changes in IL-6 levels and mean baseline values (β = 0.08, 95% CI: 0.03–0.13, *p* = 0.003).

**Figure 5:**
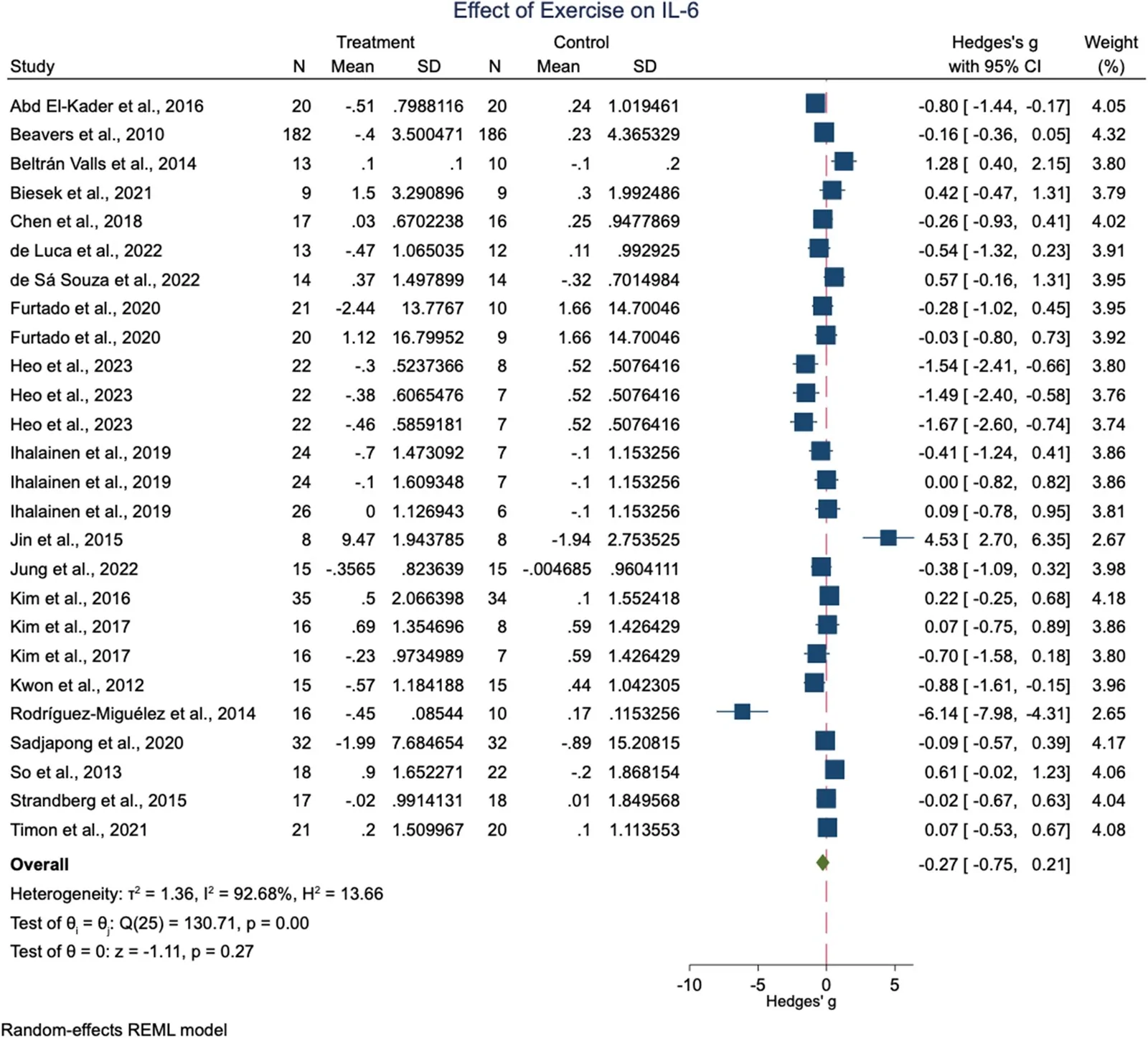
Meta-analysis of studies reporting interleukin-6. *Note.* IL-6 = Interleukin-6; REML = Restricted Maximum Likelihood.

**Figure 6:**
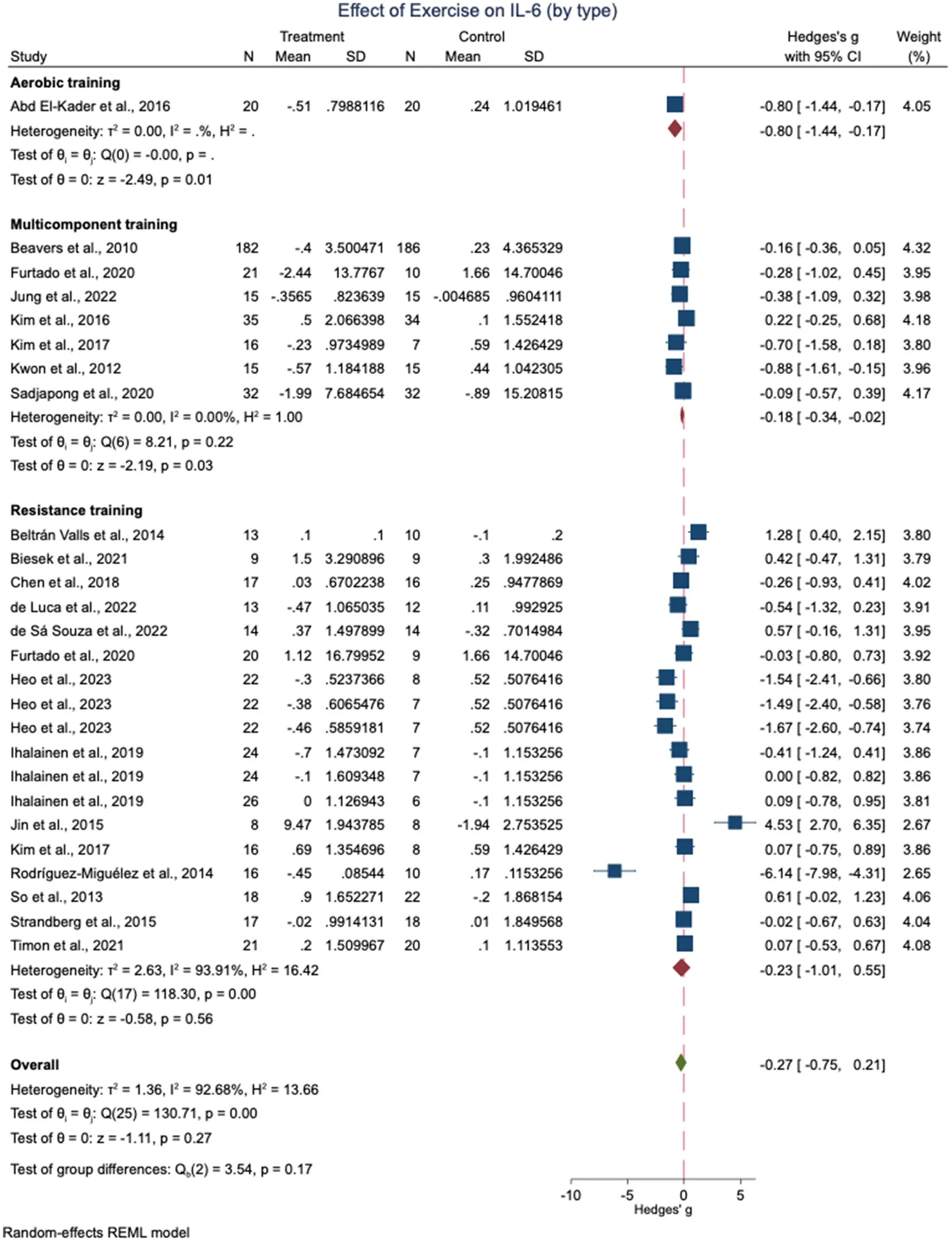
Subgroup analyses of interleukin-6 according to exercise type. *Note.* IL-6 = Interleukin-6; REML = Restricted Maximum Likelihood.

#### Interleukin-8

Two studies evaluated interleukin-8 (IL-8). The resistance training intervention did not show significant effects (Urzi et al., 2019), while the multicomponent intervention reported significantly lower levels after adjusting the linear regression model (Beavers et al., 2010).

#### Interleukin-10

Eight studies reported interleukin-10 (IL-10) (Caldo-Silva et al., 2021; de Luca Corrêa et al., 2022; de Sá Souza et al., 2022; Farinha, Ferreira, et al., 2022; Furtado et al., 2020; Heo & Jee, 2024; Tan et al., 2023; Timon et al., 2021). Two of them showed a decrease in the tumor necrosis factor-alpha (TNF-α)/interleukin (IL)-10 ratio (Farinha, Ferreira, et al., 2022; Furtado et al., 2020), and one showed a decrease in the IL-6/IL-10 ratio (Timon et al., 2021) Meta-analysis of seven studies (12 comparisons) resulted in a pooled effect size of Hedges’ g = 0.40 (95% CI: 0.18–0.63), with low heterogeneity (I^2^ = 0%)(Figure 7 ). Subgroup analyses revealed significant effects of multicomponent (Hedges’ g = 0.50, 95% CI 0.02 to 0.98) and resistance training (Hedges’ g = 0.43, 95% CI: 0.14–0.71), with low heterogeneity (I^2^ = 0%) and without significant interaction between these exercise types [Q_b_(2) = 0.91, *p* = 0.63] (Figure 8).

**Figure 7:**
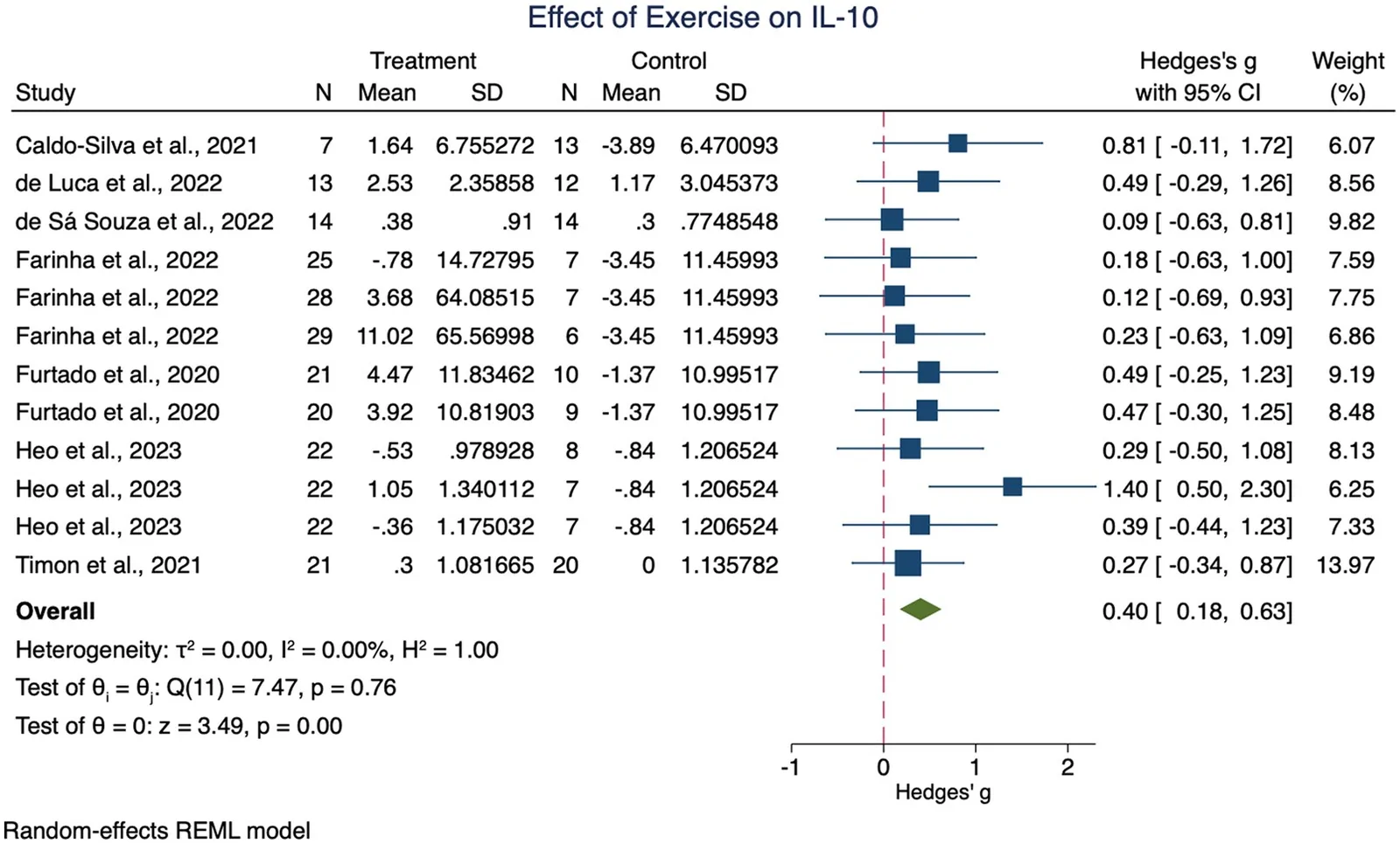
Meta-analysis of studies reporting interleukin-10. *Note.* IL-10 = Interleukin-10; REML = Restricted Maximum Likelihood.

#### Interleukin-15

Three studies evaluated interleukin-15 (IL-15) (Beavers et al., 2010; Beltran Valls et al., 2014; Urzi et al., 2019). One of them (Beavers et al., 2010) reported a decrease in those participants with levels of IL-15 above the median. Meta-analysis of all studies did not show any significant effect (Hedges’ g = 0.29, 95% CI: −0.34 to 0.93), with high heterogeneity (I^2^ = 81.4%) (Figure S4).

#### Tumor necrosis factor–alpha

TNF*-*α was reported in 12 studies (Abd El-Kader & Al-Jiffri, 2016; Beavers et al., 2010; Chen et al., 2018; de Luca Corrêa et al., 2022; de Sá Souza et al., 2022; Farinha, Ferreira, et al., 2022; Furtado et al., 2020; Heo & Jee, 2024; Phillips et al., 2010; Ruiz et al., 2015; So et al., 2013; Tan et al., 2023). Similar to IL-6, a study assessing acute responses to resistance training found a blunted post-exercise increase in TNF-α after the intervention period (Phillips et al., 2010). When comparing training intensities, one study detected significant decreases only after a high-intensity resistance protocol (Heo & Jee, 2024). Finally, two aerobic training interventions showed a decrease in TNF-α/IL-10 ratio (Farinha, Ferreira, et al., 2022; Furtado et al., 2020).

Meta-analysis of 12 studies (17 comparisons) showed no significant overall effect (Hedges’ g = −0.28, 95% CI: −0.67 to 0.10), with high heterogeneity (I^2^ = 83%) (Figure 9). Subgroup analyses revealed a significant effect of resistance training (Hedges’ g = −0.67, 95% CI: −1.27 to −0.08), also with high heterogeneity (I^2^ = 78.7%), without significant interaction between the exercise types [Q_b_(2) = 4.02, *p* = 0.13] (Figure 10). Meta-regressions showed significant associations with age (β = 0.06, 95% CI: 0.01–0.11, *p* = 0.03) and frequency of the sessions (β = −0.81; 95% CI: −1.56 to −0.06; *p* = 0.04).

#### Other parameters

No changes in interferon-gamma (IFN-γ) after aerobic training (Furtado et al., 2020); in IFN-γ (Furtado et al., 2020), resistin (Urzi et al., 2019), IL-4 and IL-13 (Beltran Valls et al., 2014) after resistance training; or in soluble tumor necrosis factor receptor I (sTNFRI) (Beavers et al., 2010; Bouchonville et al., 2014), erythrocyte sedimentation rate (ESR) (Kucharski et al., 2019; Scano et al., 2023), growth differentiation factor 15 (GDF-15) (Tan et al., 2023) or IL-6sR, IL-1sRII, soluble tumor necrosis factor receptor II (sTNFRII), and soluble interleukin-2 receptor alpha (IL-2sRα) (Beavers et al., 2010) after multicomponent interventions were observed. A multicomponent intervention reported no changes in sTNFRII (Beavers et al., 2010), while another saw an increase (Kapasi et al., 2003). Both continuous aerobic training and multicomponent training reduced monocyte chemoattractant protein-1(MCP-1), but only the latter also reduced macrophage inflammatory protein-1 alpha 1 (MIP-1α) (Farinha, Santos, et al., 2022), while a resistance training intervention did not report changes in macrophage inflammatory protein-1 alpha 1 (MIP-1ß) (Beltran Valls et al., 2014). One study found a reduction in arachidonic acid after resistance training (Strandberg et al., 2015).

### Assessment of immune status

#### White blood cell counts and characterization

Interval aerobic training and resistance training showed an increase in leukocyte count and granulocytes, with a between-group difference also in monocytes (Farinha, Ferreira, et al., 2022), and another resistance training intervention reported significant increases in both leukocytes and monocytes immediately after the exercise and training period and a lower increase in lipopolysaccharide (LPS)-stimulated production of IL-1ß, IL-6, and TNF-α in plasma (per monocyte) (Phillips et al., 2010). Two multicomponent interventions did not report any changes in leukocyte count (Caldo-Silva et al., 2023; Ko et al., 2014). Meta-analysis of four studies (eight comparisons) that evaluated lymphocyte counts did not show any significant effect (Hedges’ g = −0.07, 95% CI: −0.29 to 0.15), with low heterogeneity (I^2^ = 0%), and neither did the subgroup analyses (Figures S5 and S6).

While no changes were observed overall after the resistance training intervention, an increase in NK cells and a decrease in CD4+/CD8+ were observed immediately after the exercise (Flynn et al., 1999). On the contrary, another study evaluating resistance training at different intensities found increases in absolute counts of leukocytes, NK cells, CD3, CD4, and CD8 T cells, and CD19 B cells, while the control group experienced decreases (Heo & Jee, 2024). A multicomponent intervention did not report any changes in total lymphocytes, T cells, B cells, or NK cells (Kapasi et al., 2003).

A trial evaluating the impact of resistance training showed that, only in seropositive cytomegalovirus, endurance training induced a reduction in senescent T cells and an increase in memory and naïve cells (Cao Dinh et al., 2019).

#### Analysis of peripheral blood mononuclear cells

A study that evaluated peripheral blood mononuclear cells (PBMCs) after endurance and resistance training reported changes in the expression of 98 genes expressing proteins (61 pro-inflammatory, 28 anti-inflammatory, and nine not fully elucidated) implicated in 33 pathways, varying depending on the type of exercise, with intensive strength training (IST) downregulating pro-inflammatory signaling pathways (Liberman et al., 2022). In the same participants, gene expression was evaluated in LPS-stimulated PBMCs, showing a differential modulation. Training changed 151 genes encoding proteins (90 pro-inflammatory, 38 anti-inflammatory, 23 not fully elucidated) involved in 29 pathways. IST blunted more inflammatory pathways, and strength endurance training activated more, while the control group showed the most inflammatory response to LPS (Salimans et al., 2024).

After a resistance training intervention, the levels of TLR2, TLR4, and HSP60 in PBMCs, and components of both MyD88-dependent and -independent pathways decreased, while IL10 mRNA, the IL-10/TNFα protein ratio, and the levels of HSP70 increased (Rodriguez-Miguelez et al., 2014). In the same cohort (Mejías-Peña et al., 2017), autophagy increased (as determined by a decrease in p62/SQSTM1 and phospho-ULK-1, and an increase in Atg12, Atg16, beclin-1, and lysosomal LAMP-2), while the inflammasome NLRP3 was decreased (NLRP3 and caspase-1/procaspase-1) and apoptosis-related proteins (Bcl-2, Bcl-xL, Bad, and caspase-3) were reduced.

A different study evaluating the effect of resistance training reported a decrease in baseline levels of HSP70, HSP27, and TRXR1 in PBMCs (Beltran Valls et al., 2014). Further analyses showed that, despite decreased baseline levels, HSP70 and HSP27 increased significantly after the graded maximal exercise test in the trained group (Ceci et al., 2014). In the same cohort, telomere length was maintained in the training group (without changes in TRF2 and Ku80), and the levels of MnSOD and global DNA methylation declined, with unchanged levels of Bcl2, Bax, and caspase-3 (Dimauro et al., 2016). The same study also found a positive correlation between telomere length and functional power and strength test and negative correlations with 6-min walking speed and 6-min walking speed loaded.

### Assessment of oxidative stress

Six studies (in eight reports) evaluated oxidative stress (Alghadir et al., 2016; Beltran Valls et al., 2014; Ceci et al., 2014; Dimauro et al., 2016; Fatouros et al., 2004; Phoemsapthawee et al., 2016; Son et al., 2017; Tarazona-Santabalbina et al., 2016), reporting improvements in all of them.

Meta-analysis of the three studies that evaluated malondialdehyde (MDA) (Alghadir et al., 2016; Ceci et al., 2014; Fatouros et al., 2004) did not show any significant effect (Hedges’ g = −0.59, 95% CI: −2.08 to 0.69) (Figure S7).

A resistance training intervention did not show any significant changes in concentrations of nitrite/nitrate (de Luca Corrêa et al., 2022), while a multicomponent exercise program elicited an increase in the intervention group (with a decrease in the control group) (Son et al., 2017). In participants older than 70 years old, both a resistance program (Ceci et al., 2014) and a multicomponent program in people with frailty (Tarazona-Santabalbina et al., 2016) led to lower increases of protein carbonyls in the intervention groups. Two aerobic interventions reported increases in total antioxidant capacity (Alghadir et al., 2016; Fatouros et al., 2004). Those studies also reported a decrease in 3-nitrotyrosine levels and an increase in glutathione peroxidase (Fatouros et al., 2004), as well as a greater decrease in 8-hydroxyguanine (Alghadir et al., 2016). Another aerobic intervention also reported a decrease in 8-iso-PGF2α (Phoemsapthawee et al., 2016), while a resistance training program showed decreases in myeloperoxidase (MPO) (Beltran Valls et al., 2014) and a higher reduced glutathione (GSH)/oxidized glutathione (GSSG) ratio immediately after the exercise (Ceci et al., 2014). On the contrary, a multicomponent intervention did not report any significant changes in MPO (Caldo-Silva et al., 2021).

### Risk of bias in studies

The risk of bias summary plot, shown in Figure 11, reveals considerable variability across domains. Overall, 53% of studies (24) were classified as “High risk” and 40% (18) as “Some concerns”, with only 7% (3) rated as “Low risk.” The greatest concerns were observed in “Bias due to deviations from intended interventions” and “Bias due to missing outcome data.” Some studies failed to use appropriate intention-to-treat analyses to estimate the effect of assignment to intervention. Additionally, several studies failed to report results for a portion of the randomized participants due to missing blood samples. Since the reasons for these missing data were often not disclosed, it remains unclear whether the exclusion of these participants influenced the estimated treatment effects. In contrast, “Bias in measurement of the outcome” and “Bias in selection of the reported result” showed the highest proportions of “Low risk.” The full Rob-2 risk of bias traffic light is shown in the Supplementary File (Figure S8).

**Figure 8:**
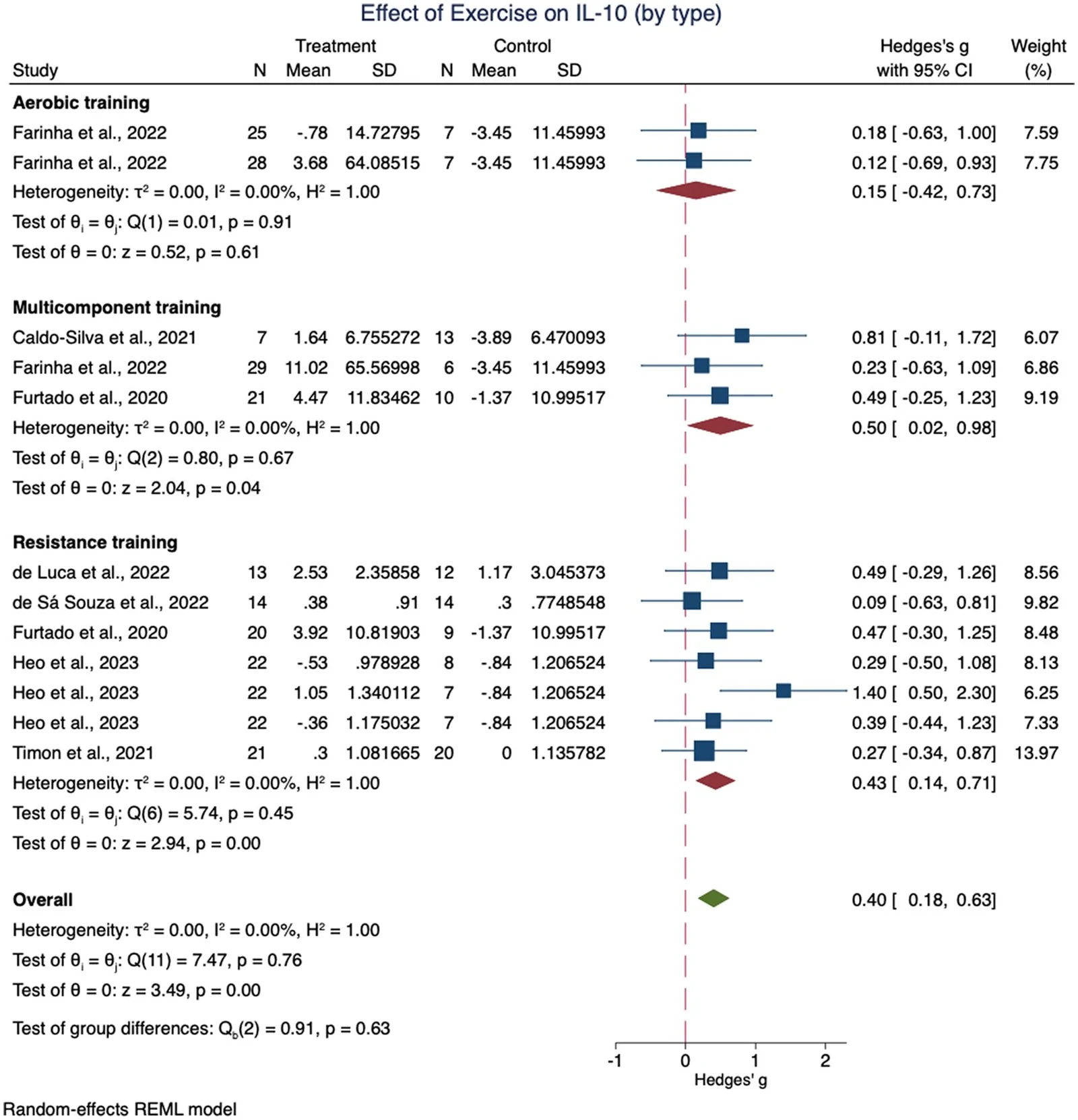
Subgroup analyses of interleukin-10 according to exercise type. *Note.* IL-10 = Interleukin-10; REML = Restricted Maximum Likelihood.

Publication bias was suggested by funnel plot asymmetry across all meta-analyses (Figures S9–S18), and by the Egger test for CRP and IL-5, although trim-and-fill analyses did not impute missing studies for CRP and two studies for IL-15, without changing the nonsignificant effect.

**Figure 9:**
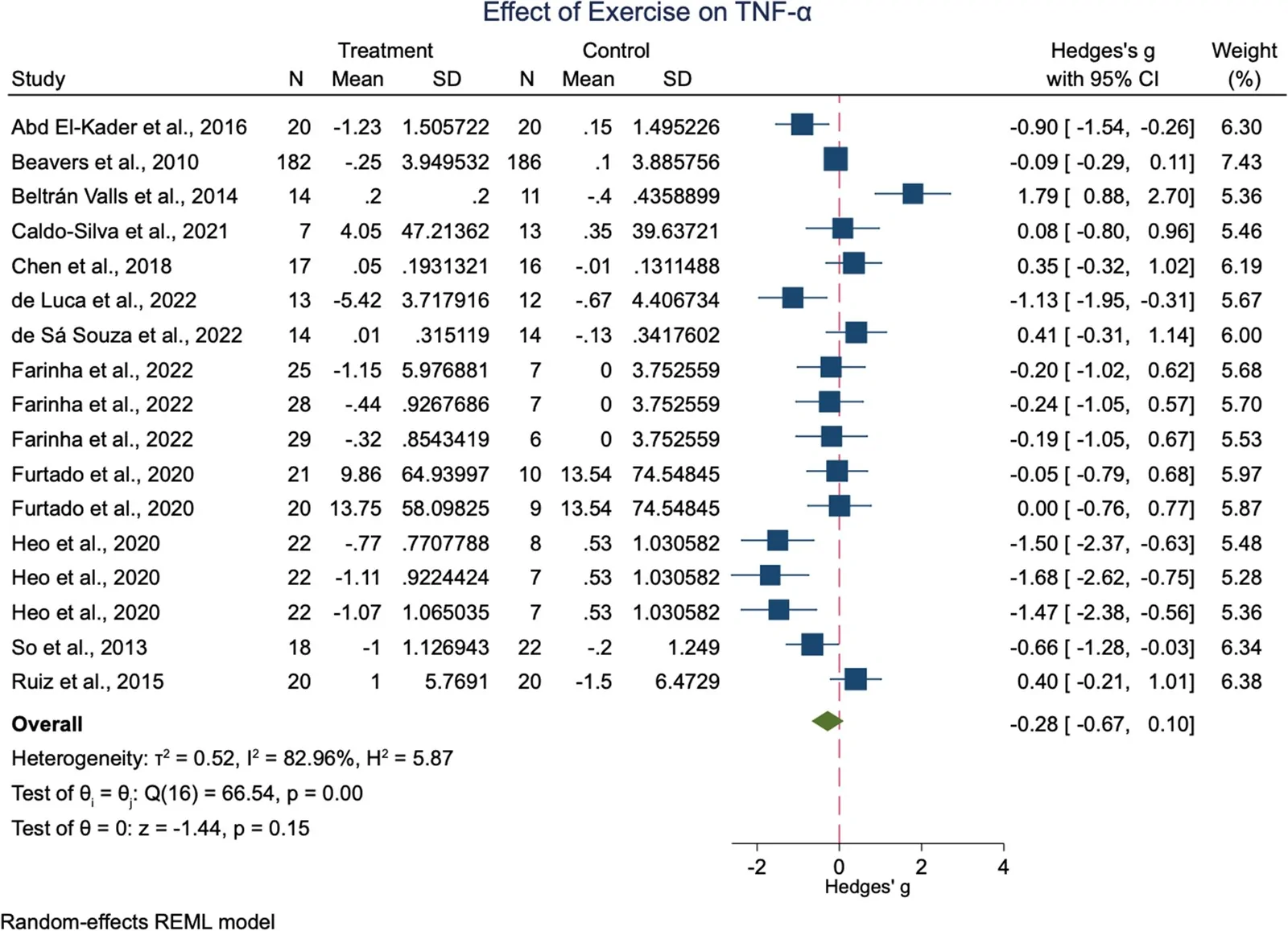
Meta-analysis of studies reporting tumor necrosis-α. *Note.* REML = Restricted Maximum Likelihood; TNF-α = Tumor Necrosis Factor alpha.

**Figure 10:**
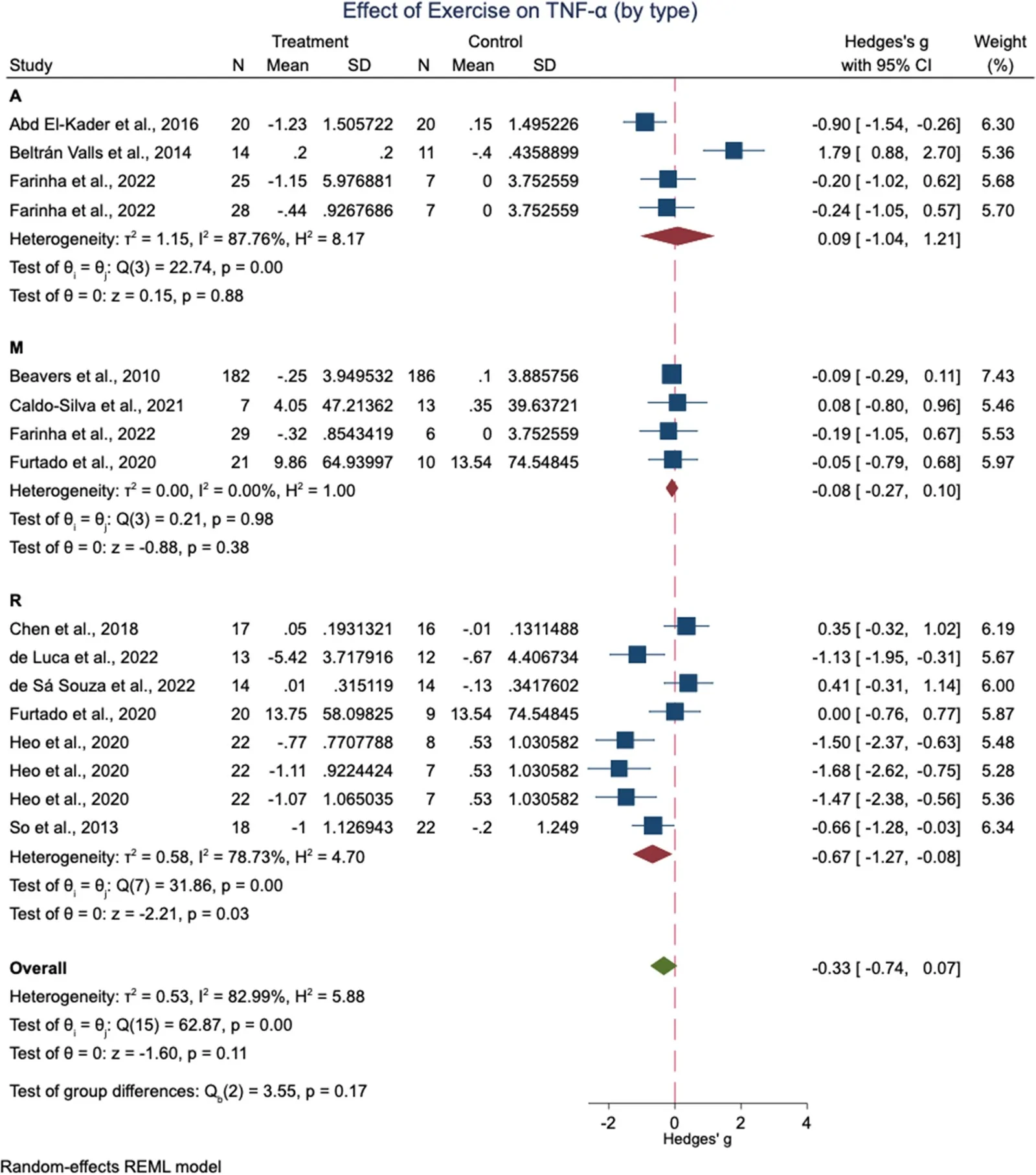
Subgroup analyses of tumor necrosis-α according to exercise type. *Note.* REML = Restricted Maximum Likelihood; TNF-α = Tumor Necrosis Factor alpha.

**Figure 11:**
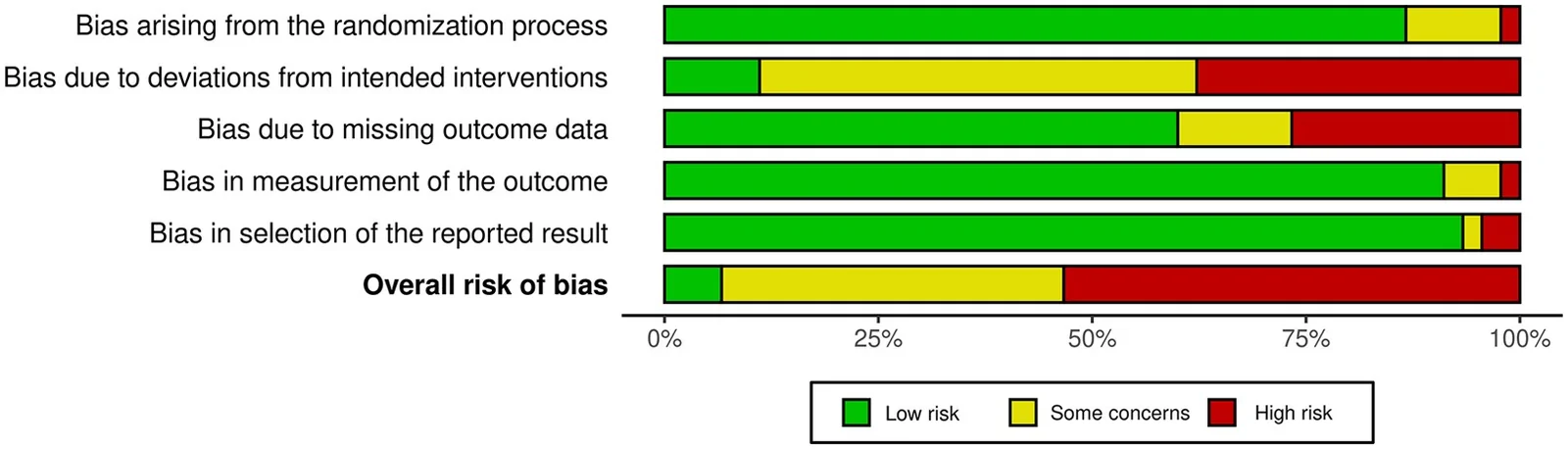
Risk of bias summary plot.

Sensitivity analysis varied across outcomes. While the pooled estimates for CRP, hsCRP, IL-15, TNF-*α* and MDA were sensitive to the exclusion of specific reports, extreme outliers with standardized residuals were not identified for either of the parameters. For IL-6, two studies were detected to have high standardized residuals (|>3|) (Jin et al., 2015; Rodriguez-Miguelez et al., 2014), after removal of which heterogeneity decreased from 92.7% to 72%, with the overall effect not being changed. (Figures S19–S28).

## Discussion

The objective of this systematic review and meta-analysis was to evaluate the current evidence supporting the impact of structured physical exercise on markers of inflammation and oxidative stress in older adults. Collectively, the evidence suggests modest but statistically significant benefits of exercise on some markers (most notably hs-CRP and IL-10), while effects on other classic pro-inflammatory cytokines such as IL-6 or TNF-α were inconsistent. Subgroup analyses revealed the potential impact of type of exercise on the modulation of inflammation, although none of them showed a statistically significant result in tests of group differences. Meta-regression analyses indicated that baseline biomarker concentrations, age, or session frequency moderated some of these effects, highlighting the complexity of exercise immunomodulation in older adults.

CRP and hs-CRP serve as well-established surrogate markers of systemic inflammation (Sun et al., 2025), and predictors of negative outcomes in older adults (Ridker, 2001). In this context, the reductions observed in the present review are clinically meaningful. In contrast, the effects on IL-6 and TNF-α were heterogeneous, manifesting the complexity of these mediators in the context of muscular activity; that is, the diversity of results related to IL-6 may stem from its transient post-exercise increase and its dual role as a signal for local metabolic processes and tissue repair (Forcina et al., 2022). Lastly, the findings related to IL-10, a key anti-inflammatory cytokine (Iyer & Cheng, 2012), showed consistent increases across studies, while findings for IL-1Ra were more limited; however, the overall direction of effect suggests a trend toward enhanced anti-inflammatory regulation. Previous evidence has shown smaller to nonsignificant changes in inflammatory markers in healthy adults free of chronic conditions (Khalafi et al., 2023), with greater benefits seen in younger individuals with chronic conditions (Sheldon et al., 2026). Other studies have evaluated the impact of different pharmacological and nutraceutical interventions on these inflammatory markers, reporting statistically significant reductions of CRP and hs-CRP following curcumin (Gorabi et al., 2022), resveratrol (Gorabi et al., 2021), omega-3 fatty acids, lipid-lowering therapies (Xie et al., 2024), and metformin (Karbalaee-Hasani et al., 2021), or reduction in other inflammatory markers after targeted biologics (Imbesi et al., 2025; Mensah et al., 2026; Zhao et al., 2026). However, direct comparisons of these effects remain challenging, as most of these interventions have been tested in specific clinical populations characterized by distinct inflammatory profiles. Moreover, exercise has proven to exert broader systemic benefits and simultaneously address multiple drivers of inflammation, including both metabolic drivers (adipose tissue) and muscular drivers (lean muscle mass) (Villareal et al., 2011), and rather than suppressing specific markers, the literature suggests its effects as a systemic *immunomodulator* (Sellami et al., 2018). These findings are also consistent with the analyses reported on PBMCs, which may reflect a reduced chronic systemic inflammation without compromising the body’s defense mechanisms, and a possible temporal shift in gene expression induced by physical exercise interventions.

Across the studies that assessed oxidative stress, the majority reported improvements in redox homeostasis, both through reduction in damage markers and increases in certain antioxidant defenses. Despite high heterogeneity, physical exercise seemed to effectively enhance redox balance. Evidence from one study suggests a hormetic adaptation, where exercise lowers oxidative stress despite decreases in some antioxidant enzymes. This is supported by the several mechanisms that have been proposed through which physical exercise influences redox homeostasis, with effects that vary according to exercise intensity and modality, highlighting the complexity and systemic nature of these adaptations (Kruk et al., 2019). These adaptations, in turn, also influence the secretion of exercise-induced signaling molecules (exerkines), which act systemically to modulate the inflammatory tone, thereby potentially reshaping the individual’s inflammatory status and orchestrating the cross-talk among other organs (Caporossi et al., 2026).

Evidence is still limited regarding the effects of physical exercise on the immune cell populations, autophagy, apoptosis, telomere length, and the rest of the evaluated biomarkers. Nevertheless, available data indicate that exercise may promote autophagy, preserve telomere length, strengthen PBMC function, and attenuate the release of cytokines that are part of the senescence-associated secretory phenotype, underscoring potential mechanisms through which exercise directly modulates key hallmarks of aging (López-Otín et al., 2023).

These findings highlight the need to move beyond relying on isolated biomarkers, as exercise could have a complex and dynamic effect on the immune system. In this context, future trials should consider evaluating integrative markers, such as anti- to pro-inflammatory ratios. Furthermore, our results also underscore the importance of accounting for baseline inflammatory status when evaluating exercise-induced molecular changes.

Taken together, physical exercise has a positive impact on systemic inflammation, cytokine balance, PBMC signaling, oxidative stress, and cellular resilience, highlighting its multilevel immunomodulatory role, even in participants with chronic conditions such as frailty, chronic kidney disease, or cognitive impairment. Moreover, while not the focus of this review, most studies found notable benefits in the participants’ physical, cognitive, mood, and quality-of-life measures following the interventions, underscoring the broad positive effects of physical exercise on the outcomes of individual patients.

### Strengths, limitations, and future directions

The present systematic review and meta-analysis provide a powerful integration of evidence by evaluating a broad set of inflammation and oxidative stress biomarkers together. This holistic approach avoids reliance on a single, potentially misleading systemic marker and instead aims to capture the true multilevel impact of physical exercise on the aging system. Furthermore, subgroup analyses provided crucial evidence supporting the benefit of all types of exercise on different biomarkers, especially multicomponent and resistance exercise, and reinforcing the use of these protocols in older adults.

The primary limitation of this analysis is the high degree of heterogeneity across studies. This limitation likely reflects the lack of standardization in research protocols, molecular assays, and patient populations. In some cases, it restricts the certainty of the pooled effect sizes, introducing uncertainty and suggesting that the results should be interpreted with caution. Moreover, a high overall risk of bias was identified in a substantial proportion of studies, especially related to deviations from intended interventions, potentially compromising the internal validity and limiting the reliability of the results. Finally, given the rapidly expanding body of evidence in this field, relevant studies published after the search date may not have been captured.

Future studies should prioritize the simultaneous measurement of ratio markers, standardize molecular assays, and use cellular analysis to identify key regulatory pathways and molecular targets of intervention. From a practical perspective, our findings suggest that not all exercise prescriptions are equally effective, and that benefits are likely influenced not only by the type of exercise but also by how the intervention is delivered (i.e., frequency, duration, and overall training exposure). A critical gap exists in understanding molecular dose-response relationships, pointing to the need for stratified study designs to determine the optimal intensity, frequency, and volume of resistance and aerobic training required to achieve maximal anti-inflammatory and antioxidant adaptations in older adults with specific comorbidities.

## Conclusions

The present systematic review provides quantitative evidence that physical exercise effectively modulates the molecular drivers of inflammation and oxidative stress through its effects on toll-like receptor (TLR) signaling, the inflammasome, various cytokines, and the cellular antioxidant reserve. Multicomponent and resistance training emerge as the most effective modalities. Overall, these findings reinforce the concept that structured exercise is a key non-pharmacological intervention capable of mitigating inflammaging.

## Supplementary Material

gnag228_Supplementary_Data

## Data Availability

The datasets generated and analyzed during the current study, as well as the analytic methods and materials, are available from the corresponding author upon reasonable request. The studies reported in this manuscript were not pre-registered.
