# Supplementary material for: Impact of supervised physical exercise on inflammatory and oxidative stress biomarkers in older adults: a systematic review and meta-analysis of randomized controlled trials
Source: Gerontologist. 2026 Sep 24;66(11):gnag228. doi: 10.1093/geront/gnag228 (PMC13730600; doi:10.1093/geront/gnag228)
Supplement: Supplementary file 1 [file gnag228_supplementary_data.pdf]

Impact of supervised physical exercise on inflammatory and oxidative stress biomarkers in older adults: a systematic review and meta-analysis of randomized controlled trials

AUTHORS

Chenhui Chenhuichen, Yasmin Ezzatvar, Fabiola Zambom-Ferraresi, Carly Welch, Myriam Gorospe, Marta Montes Resano, Marta Gutiérrez-Valencia, Juan José Lasarte Sagastibelza, Thomas Jackson, Nicolás Martínez-Velilla

Supplementary Electronic Material

Table S1. PRISMA Checklist

Section and Topic	Item #	Checklist item	Location where item is reported
**TITLE**			
Title	1	Identify the report as a systematic review.	Title
**ABSTRACT**			
Abstract	2	See the PRISMA 2020 for Abstracts checklist.	Abstract
**INTRODUCTION**			
Rationale	3	Describe the rationale for the review in the context of existing knowledge.	Introduction
Objectives	4	Provide an explicit statement of the objective(s) or question(s) the review addresses.	Page 2
**METHODS**			
Eligibility criteria	5	Specify the inclusion and exclusion criteria for the review and how studies were grouped for the syntheses.	2.2
Information sources	6	Specify all databases, registers, websites, organisations, reference lists and other sources searched or consulted to identify studies. Specify the date when each source was last searched or consulted.	2.1.
Search strategy	7	Present the full search strategies for all databases, registers and websites, including any filters and limits used.	Tables S2-S6
Selection process	8	Specify the methods used to decide whether a study met the inclusion criteria of the review, including how many reviewers screened each record and each report retrieved, whether they worked independently, and if applicable, details of automation tools used in the process.	2.2
Data collection process	9	Specify the methods used to collect data from reports, including how many reviewers collected data from each report, whether they worked independently, any processes for obtaining or confirming data from study investigators, and if applicable, details of automation tools used in the process.	2.3
Data items	10a	List and define all outcomes for which data were sought. Specify whether all results that were compatible with each outcome domain in each study were sought (e.g. for all measures, time points, analyses), and if not, the methods used to decide which results to collect.	2.3
	10b	List and define all other variables for which data were sought (e.g. participant and intervention characteristics, funding sources). Describe any assumptions made about any missing or unclear information.	2.3
Study risk of bias assessment	11	Specify the methods used to assess risk of bias in the included studies, including details of the tool(s) used, how many reviewers assessed each study and whether they worked independently, and if applicable, details of automation tools used in the process.	2.4
Effect measures	12	Specify for each outcome the effect measure(s) (e.g. risk ratio, mean difference) used in the synthesis or presentation of	2.5

Section and Topic	Item #	Checklist item	Location where item is reported
		results.	
Synthesis methods	13a	Describe the processes used to decide which studies were eligible for each synthesis (e.g. tabulating the study intervention characteristics and comparing against the planned groups for each synthesis (item #5)).	2.5
	13b	Describe any methods required to prepare the data for presentation or synthesis, such as handling of missing summary statistics, or data conversions.	2.5
	13c	Describe any methods used to tabulate or visually display results of individual studies and syntheses.	2.5
	13d	Describe any methods used to synthesize results and provide a rationale for the choice(s). If meta-analysis was performed, describe the model(s), method(s) to identify the presence and extent of statistical heterogeneity, and software package(s) used.	2.5
	13e	Describe any methods used to explore possible causes of heterogeneity among study results (e.g. subgroup analysis, meta-regression).	2.5
	13f	Describe any sensitivity analyses conducted to assess robustness of the synthesized results.	2.5
Reporting bias assessment	14	Describe any methods used to assess risk of bias due to missing results in a synthesis (arising from reporting biases).	2.5
Certainty assessment	15	Describe any methods used to assess certainty (or confidence) in the body of evidence for an outcome.	
**RESULTS**			
Study selection	16a	Describe the results of the search and selection process, from the number of records identified in the search to the number of studies included in the review, ideally using a flow diagram.	3.1
	16b	Cite studies that might appear to meet the inclusion criteria, but which were excluded, and explain why they were excluded.	3.1
Study characteristics	17	Cite each included study and present its characteristics.	Table 1
Risk of bias in studies	18	Present assessments of risk of bias for each included study.	Figure S8
Results of individual studies	19	For all outcomes, present, for each study: (a) summary statistics for each group (where appropriate) and (b) an effect estimate and its precision (e.g. confidence/credible interval), ideally using structured tables or plots.	Figures 2-10, Figures S1-S7
Results of syntheses	20a	For each synthesis, briefly summarise the characteristics and risk of bias among contributing studies.	3.3. and 3.4
	20b	Present results of all statistical syntheses conducted. If meta-analysis was done, present for each the summary estimate and its precision (e.g. confidence/credible interval) and measures of statistical heterogeneity. If comparing groups, describe the direction of the effect.	Figures 2-10, Figures S1-S7

Section and Topic	Item #	Checklist item	Location where item is reported
	20c	Present results of all investigations of possible causes of heterogeneity among study results.	3.3. and 3.4
	20d	Present results of all sensitivity analyses conducted to assess the robustness of the synthesized results.	Figures S19-29
Reporting biases	21	Present assessments of risk of bias due to missing results (arising from reporting biases) for each synthesis assessed.	Figure S8
Certainty of evidence	22	Present assessments of certainty (or confidence) in the body of evidence for each outcome assessed.	
**DISCUSSION**			
Discussion	23a	Provide a general interpretation of the results in the context of other evidence.	Discussion
	23b	Discuss any limitations of the evidence included in the review.	Discussion
	23c	Discuss any limitations of the review processes used.	Discussion
	23d	Discuss implications of the results for practice, policy, and future research.	Discussion
**OTHER INFORMATION**			
Registration and protocol	24a	Provide registration information for the review, including register name and registration number, or state that the review was not registered.	2.1
	24b	Indicate where the review protocol can be accessed, or state that a protocol was not prepared.	2.1
	24c	Describe and explain any amendments to information provided at registration or in the protocol.	2.1
Support	25	Describe sources of financial or non-financial support for the review, and the role of the funders or sponsors in the review.	✓
Competing interests	26	Declare any competing interests of review authors.	✓
Availability of data, code and other materials	27	Report which of the following are publicly available and where they can be found: template data collection forms; data extracted from included studies; data used for all analyses; analytic code; any other materials used in the review.	✓

From: Page MJ, McKenzie JE, Bossuyt PM, Boutron I, Hoffmann TC, Mulrow CD, et al. The PRISMA 2020 statement: an updated guideline for reporting systematic reviews. BMJ 2021;372:n71. doi: 10.1136/bmj.n71. This work is licensed under CC BY 4.0. To view a copy of this license, visit https://creativecommons.org/licenses/by/4.0/

Table S2. Search Strategy used in PubMed

ID	Search Hits
#1	(“aged”[Mesh] OR “older adults” OR “elderly” OR “senior” OR aging”[Mesh] OR “geriatric”)
#2	(“exercise”[Mesh] OR “physical activity” OR “training” OR “exercise therapy” OR “fitness” OR “rehabilitation”)
#3	(“molecular analysis” OR “biomarkers”[Mesh] OR “gene expression”[Mesh] OR “proteomis”[Mesh] OR “metabolomics”[Mesh] OR “metabolite profiling”)
#1 AND #2 AND #3<br>Filters: English, Spanish; randomized controlled trial[publication type]	

Table S3. Search Strategy used in Cochrane Library

ID	Search Hits
#1	("Aged" OR "Older Adults" OR "Elderly" OR "Senior" OR "Aging" OR "Geriatric") in Title Abstract Keyword
#2	("Exercise" OR "Physical Activity" OR "Training" OR "Exercise Therapy" OR "Fitness" OR "Rehabilitation") in Title Abstract Keyword
#3	("Molecular Analysis" OR "Biomarkers" OR "Gene Expression" OR "Proteomics" OR "Metabolomics" OR "Metabolite Profiling") in Title Abstract Keyword
#1 AND #2 AND #3<br>Filters: English, Spanish; Trials	

Table S4. Search Strategy used in Scopus

ID	Search Hits
#1	TITLE-ABS-KEY ("Aged" OR "Older Adults" OR "Elderly" OR "Senior" OR "Aging" OR "Geriatric")
#2	TITLE-ABS-KEY ("Exercise" OR "Physical Activity" OR "Training" OR "Exercise Therapy" OR "Fitness" OR "Rehabilitation")
#3	TITLE-ABS-KEY ("Molecular Analysis" OR "Biomarkers" OR "Gene Expression" OR "Proteomics" OR "Metabolomics" OR "Metabolite Profiling")
#4	TITLE-ABS-KEY ("Randomized Controlled Trial" OR "RCT" OR "Controlled Trial" OR "Clinical Trial")
#1 AND #2 AND #3 AND #4<br>Filters: Article, Review; English, Spanish; Source: Journal; Final stage	

Table S5. Search Strategy used in CINAHL

ID	Search Hits
#1	XB (“Aged” OR "Older Adults" OR “Elderly” OR “Senior” OR “Aging” OR “Geriatric”)
#2	XB (“Exercise” OR "Physical Activity" OR “Training” OR "Exercise Therapy" OR “Fitness” OR “Rehabilitation”)

#3 XB ("Molecular Analysis" OR “Biomarkers” OR "Gene Expression" OR “Proteomics” OR “Metabolomics” OR "Metabolite Profiling")

#4 XB (RCT OR "Randomized Controlled Trial" OR "Controlled Trial" OR "Clinical Trial")

#1 AND #2 AND #3 AND #4

Filters: English, Spanish

Table S6. Search Strategy used in Web of Science

ID	Search Hits
#1	TS=(“Aged” OR "Older Adults" OR “Elderly” OR “Senior” OR “Aging” OR “Geriatric”)
#2	TS=(“Exercise” OR "Physical Activity" OR “Training” OR "Exercise Therapy" OR “Fitness” OR “Rehabilitation”)
#3	TS=("Molecular Analysis" OR “Biomarkers” OR "Gene Expression" OR “Proteomics” OR “Metabolomics” OR "Metabolite Profiling")
#4	TS=(RCT OR "Randomized Controlled Trial" OR "Controlled Trial" OR "Clinical Trial")
	#1 AND #2 AND #3 AND #4
	Filters: English, Spanish

Figure S1. Subgroup analyses of hs-CRP according to exercise type

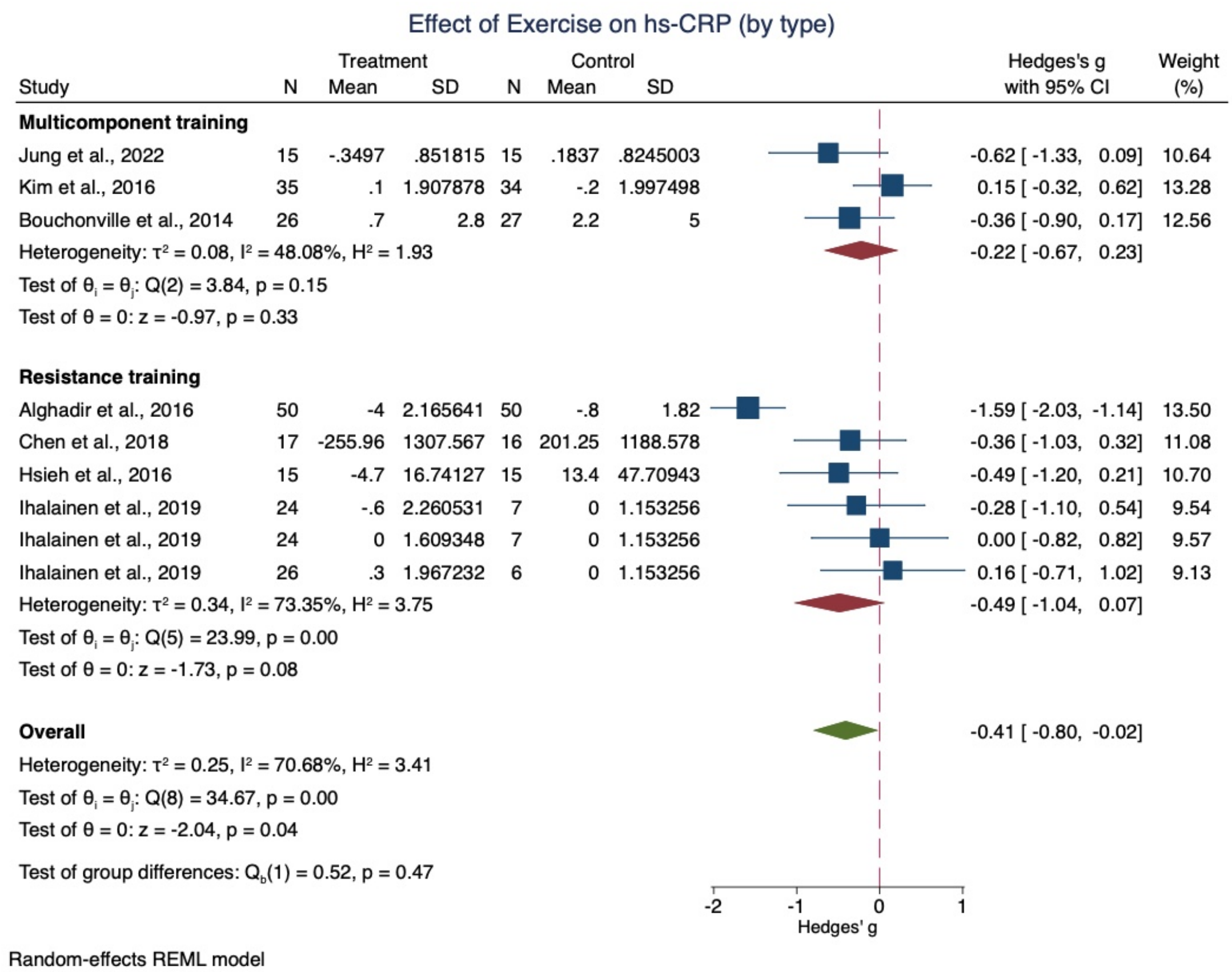

Figure S2. Meta-analyses of studies reporting IL-1ß

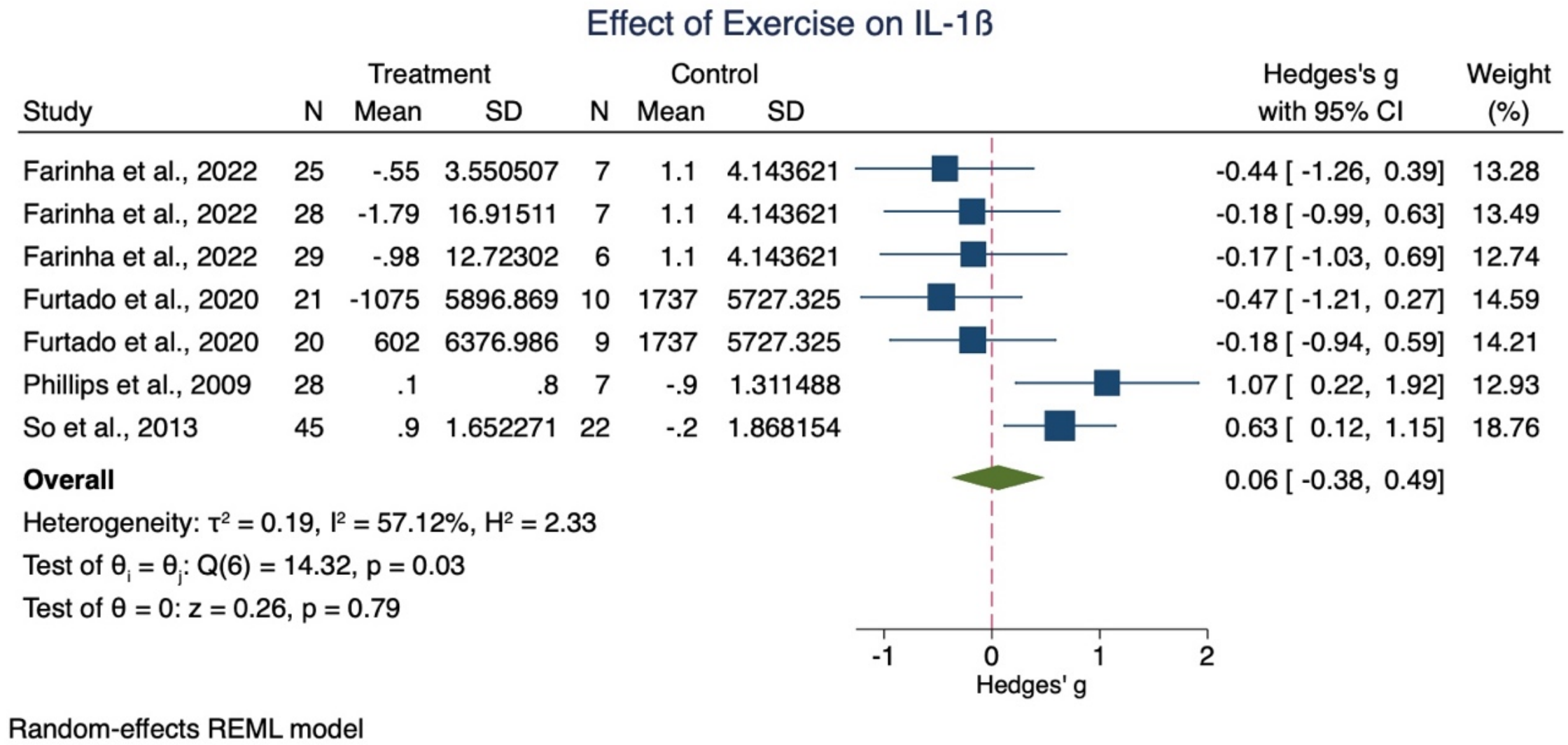

Figure S3. Meta-analyses of studies reporting IL-Ra.

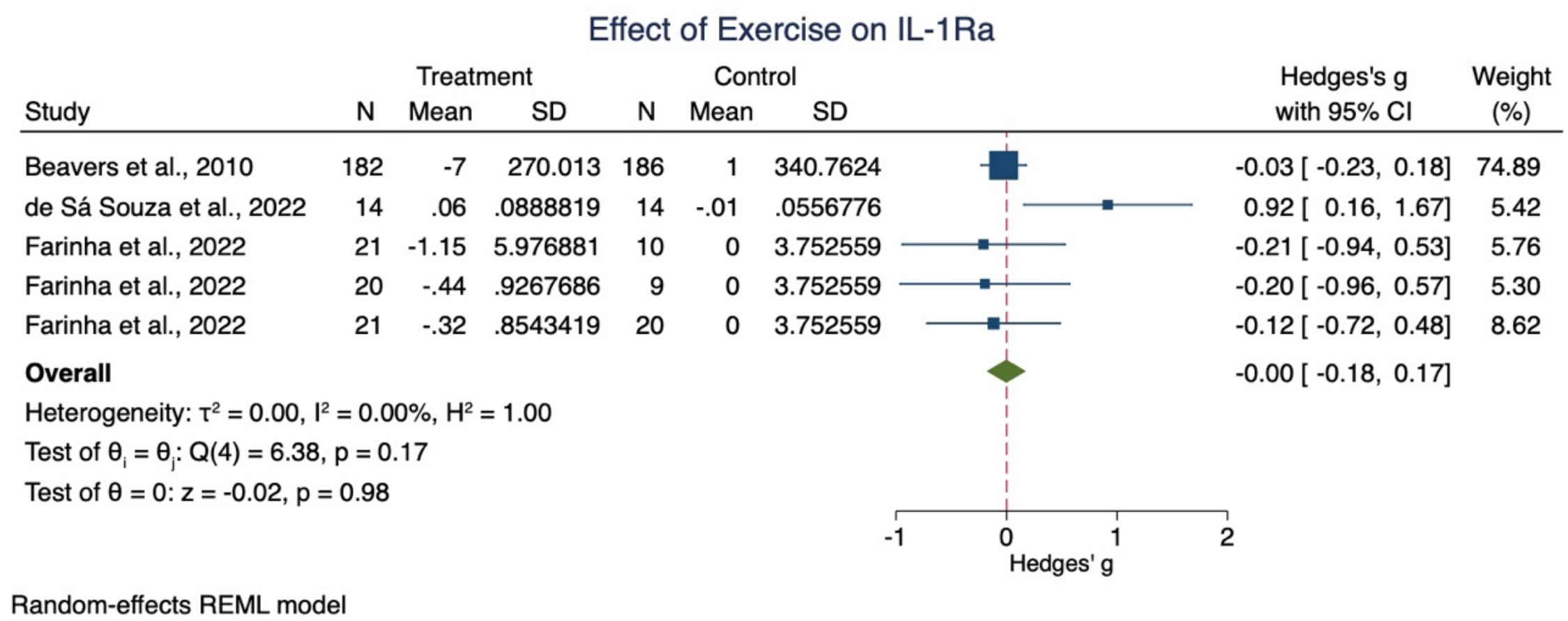

Figure S4. Meta-analyses of studies reporting IL-15.

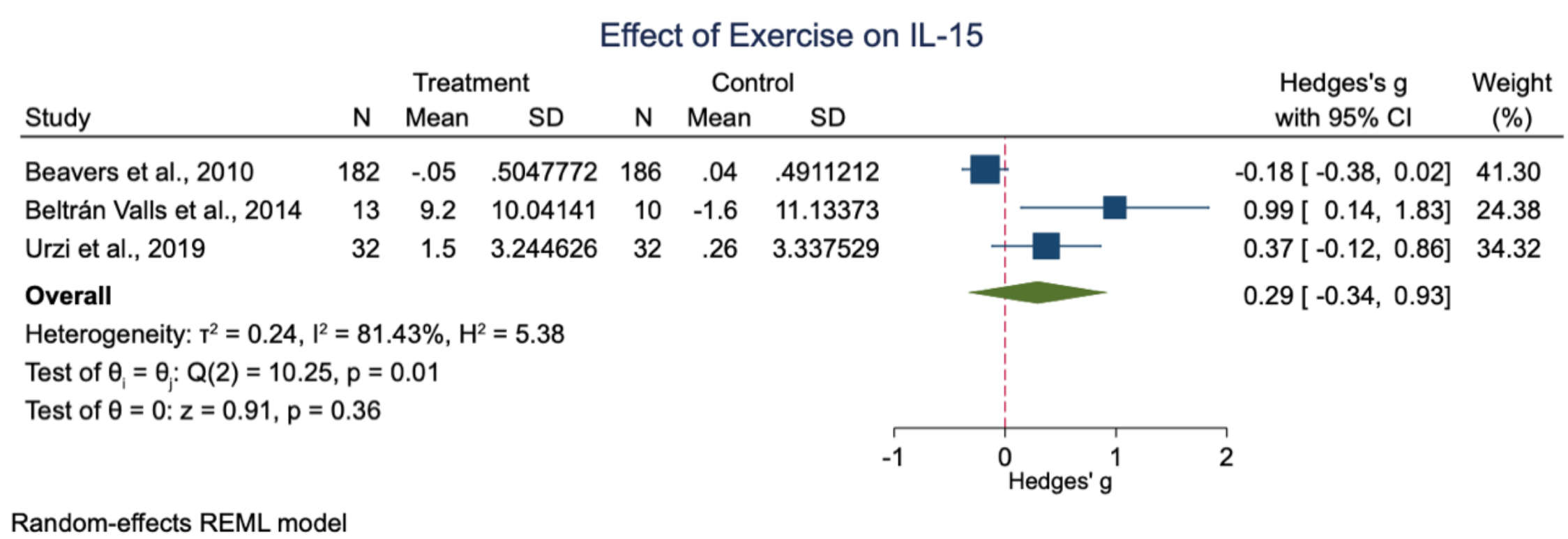

Figure S5. Meta-analyses of studies reporting lymphocytes count.

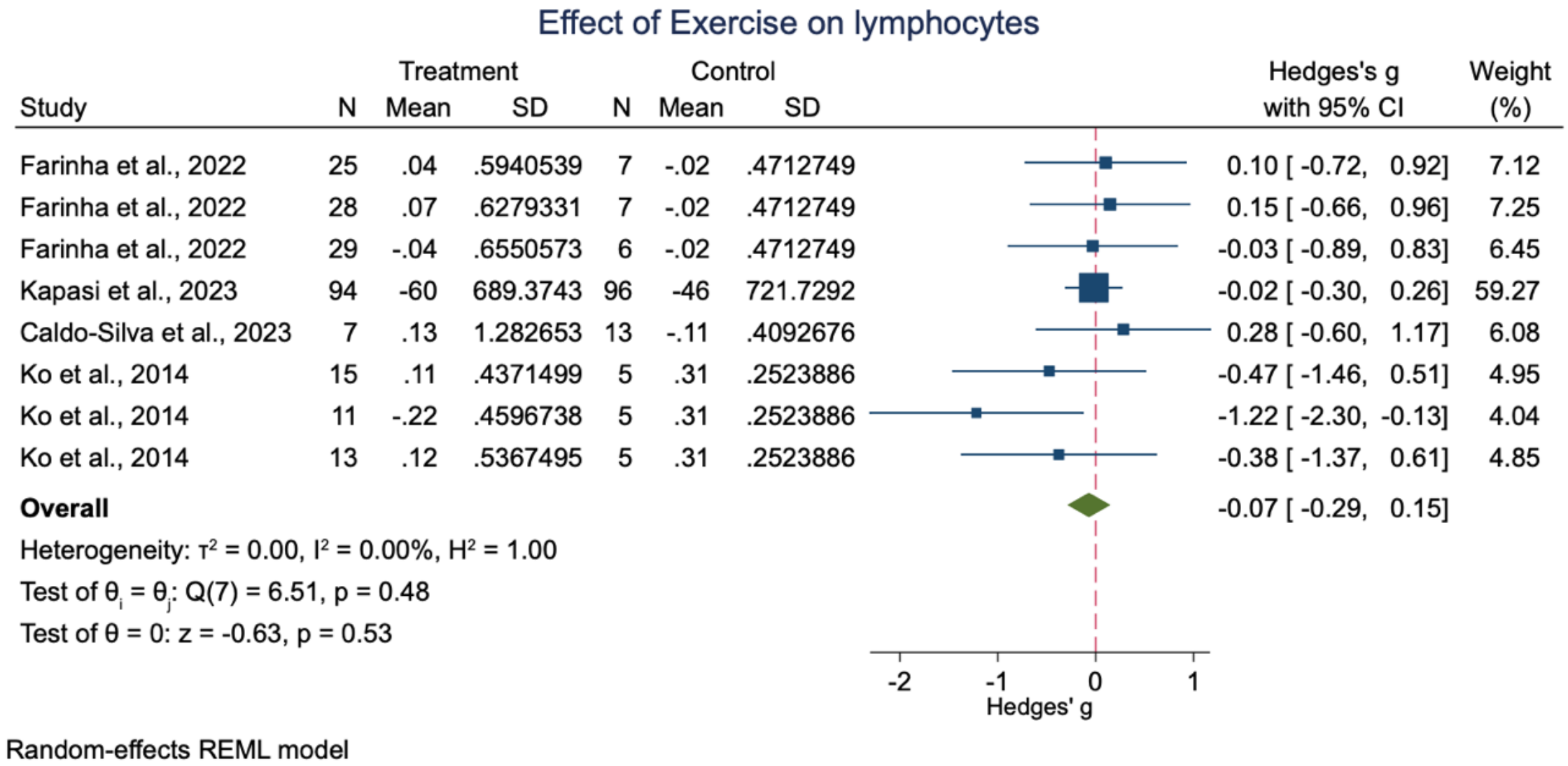

Figure S6. Subgroup analyses of lymphocytes count according to exercise type

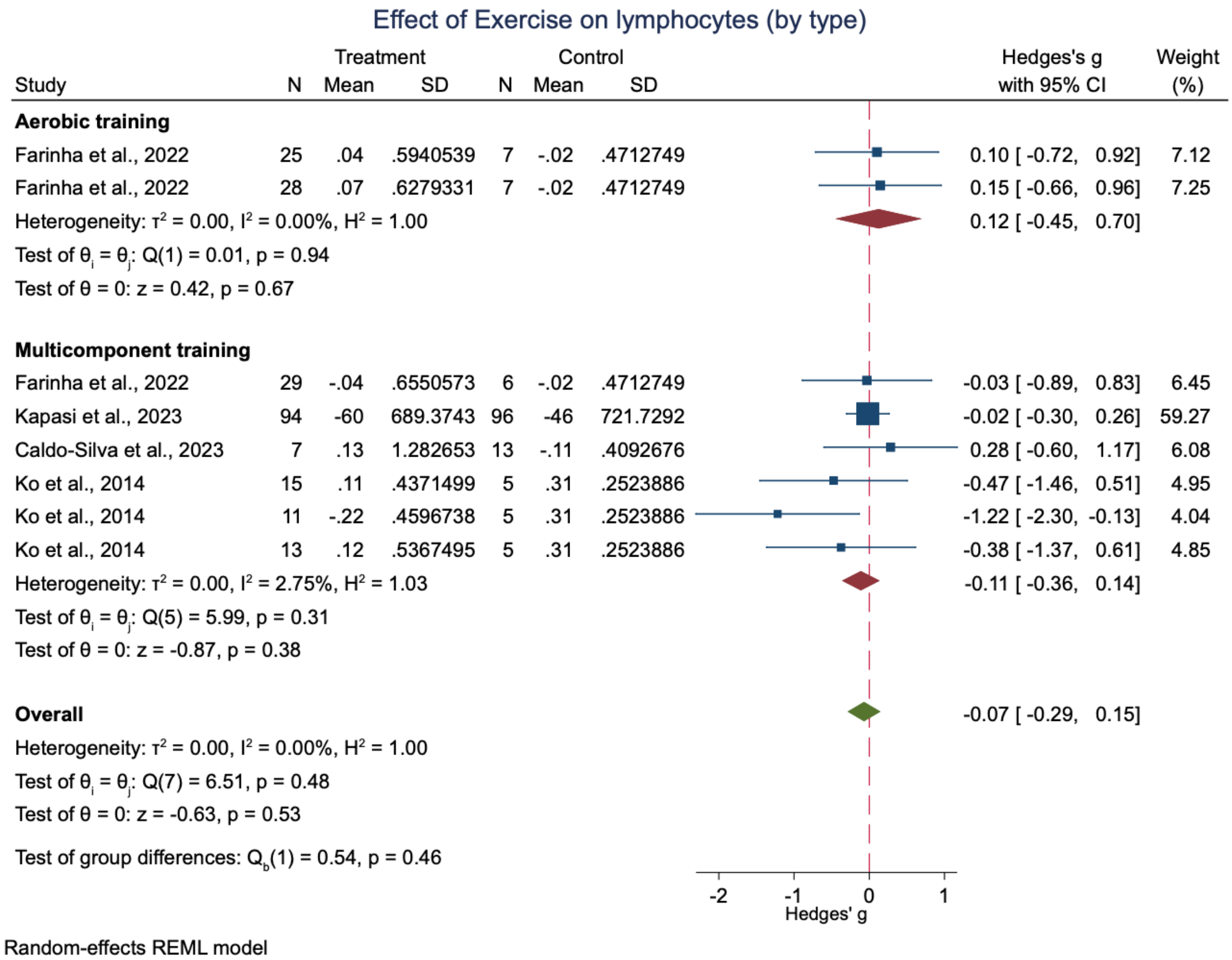

Figure S7. Meta-analyses of studies reporting MDA levels.

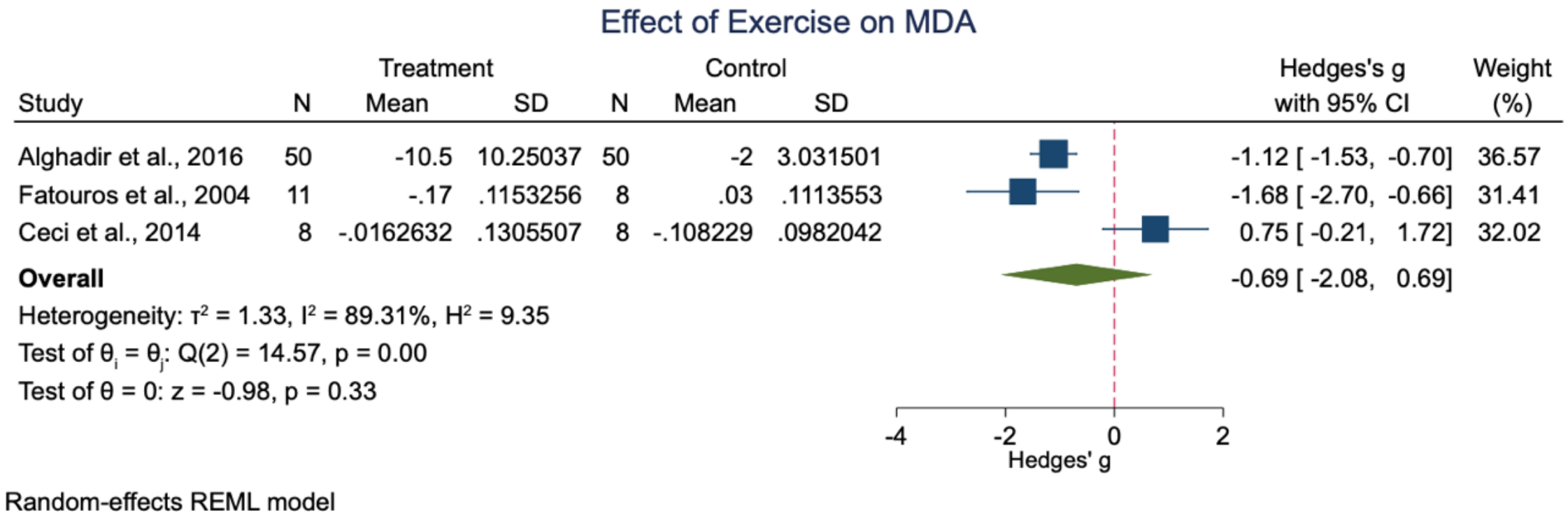

Figure S8. Rob-2 Risk of bias traffic light

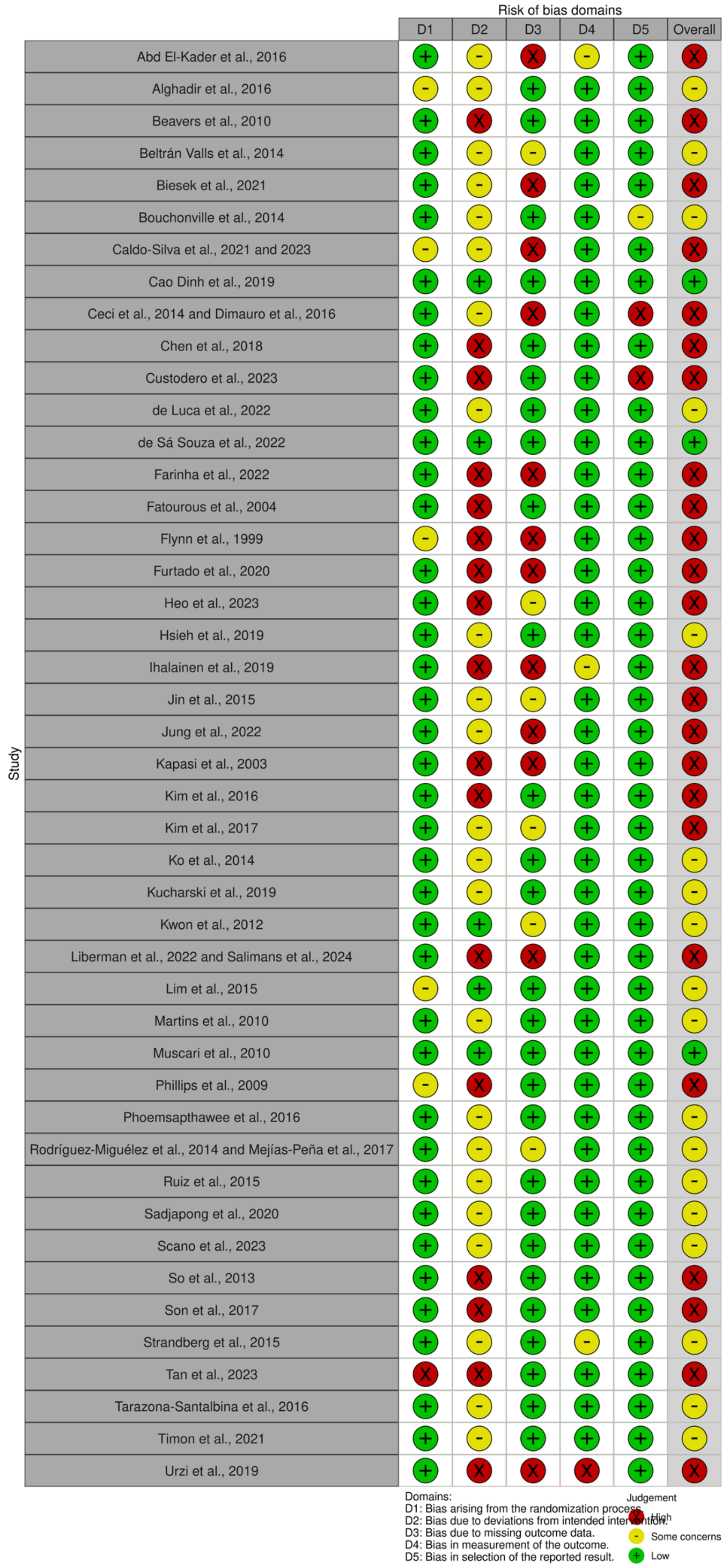

Figures S9-S18. Funnel plots

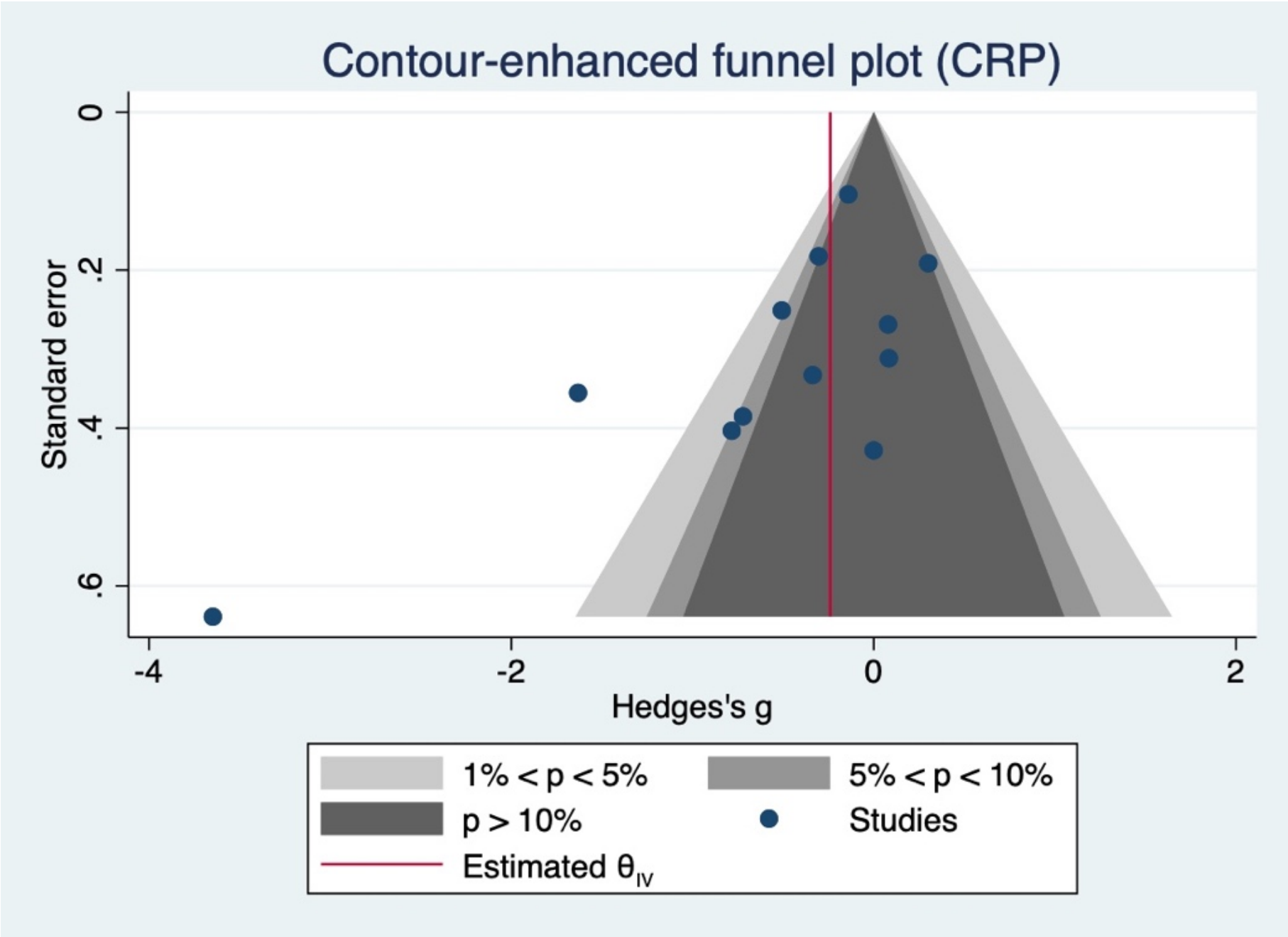

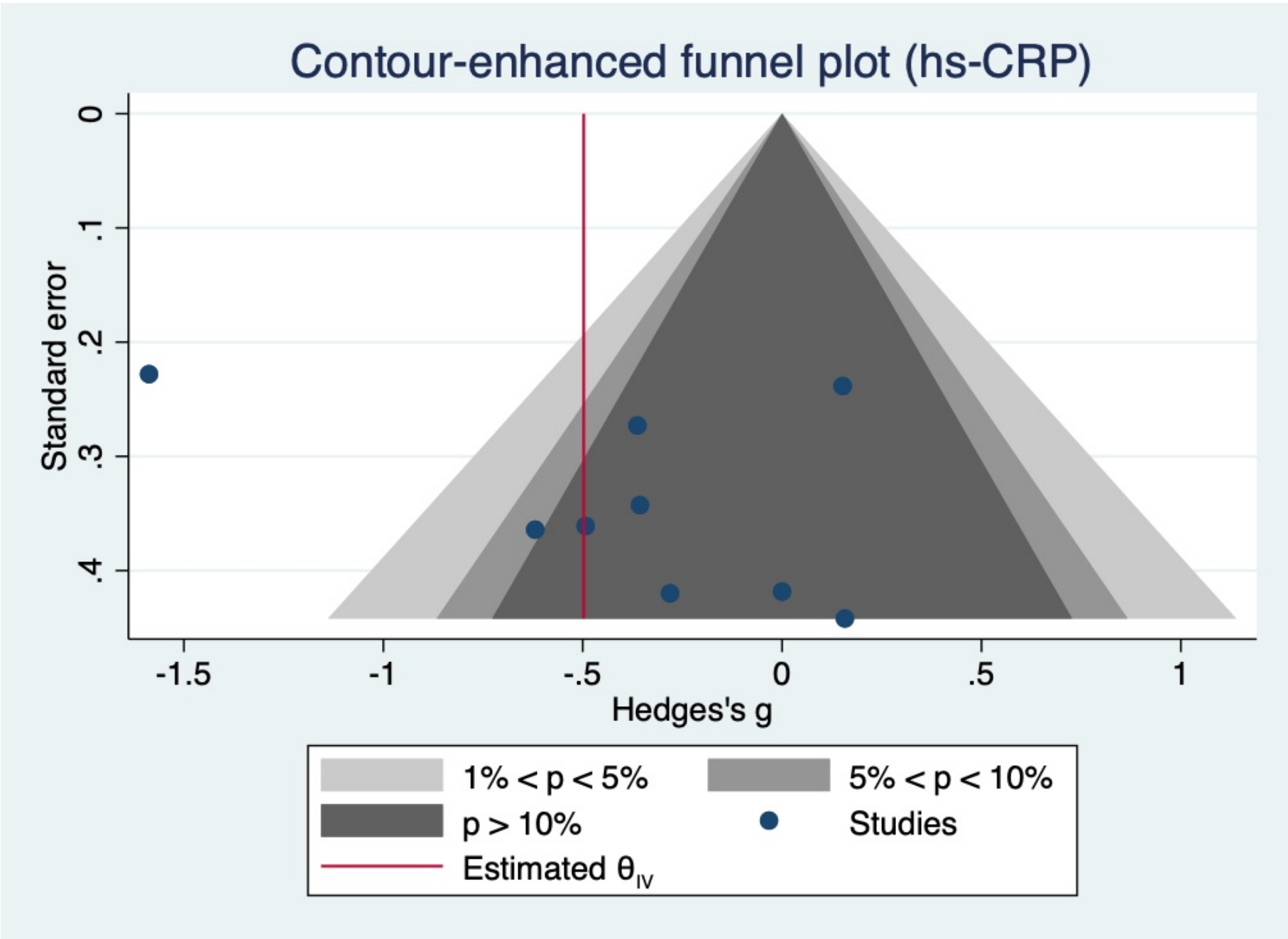

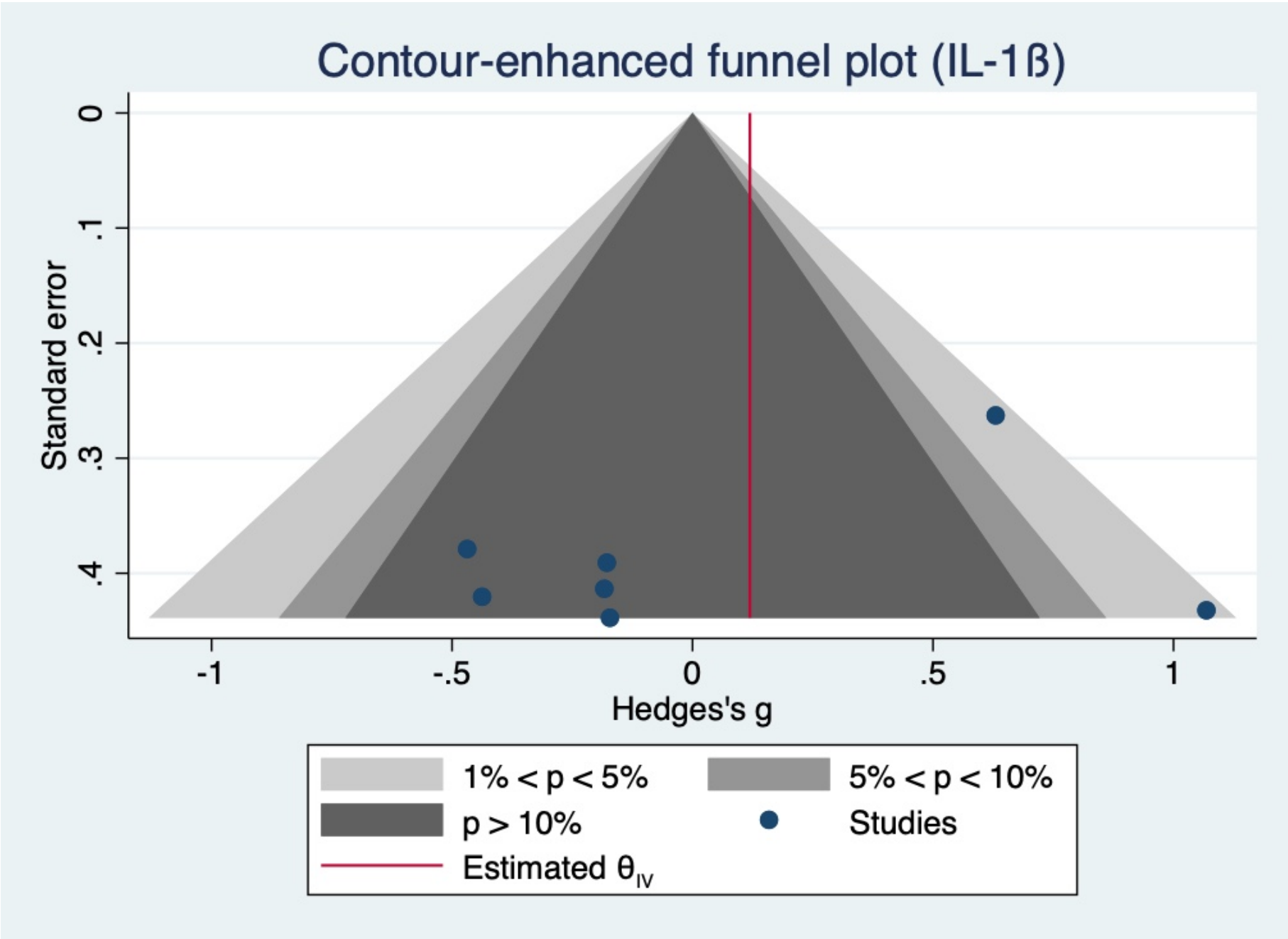
Contour-enhanced funnel plot (IL-1ß)
Standard error
0
.1
.2
.3
.4
-1
-.5
0
.5
1
Hedges's g
1% < p < 5%
5% < p < 10%
p > 10%
Studies
Estimated θIV

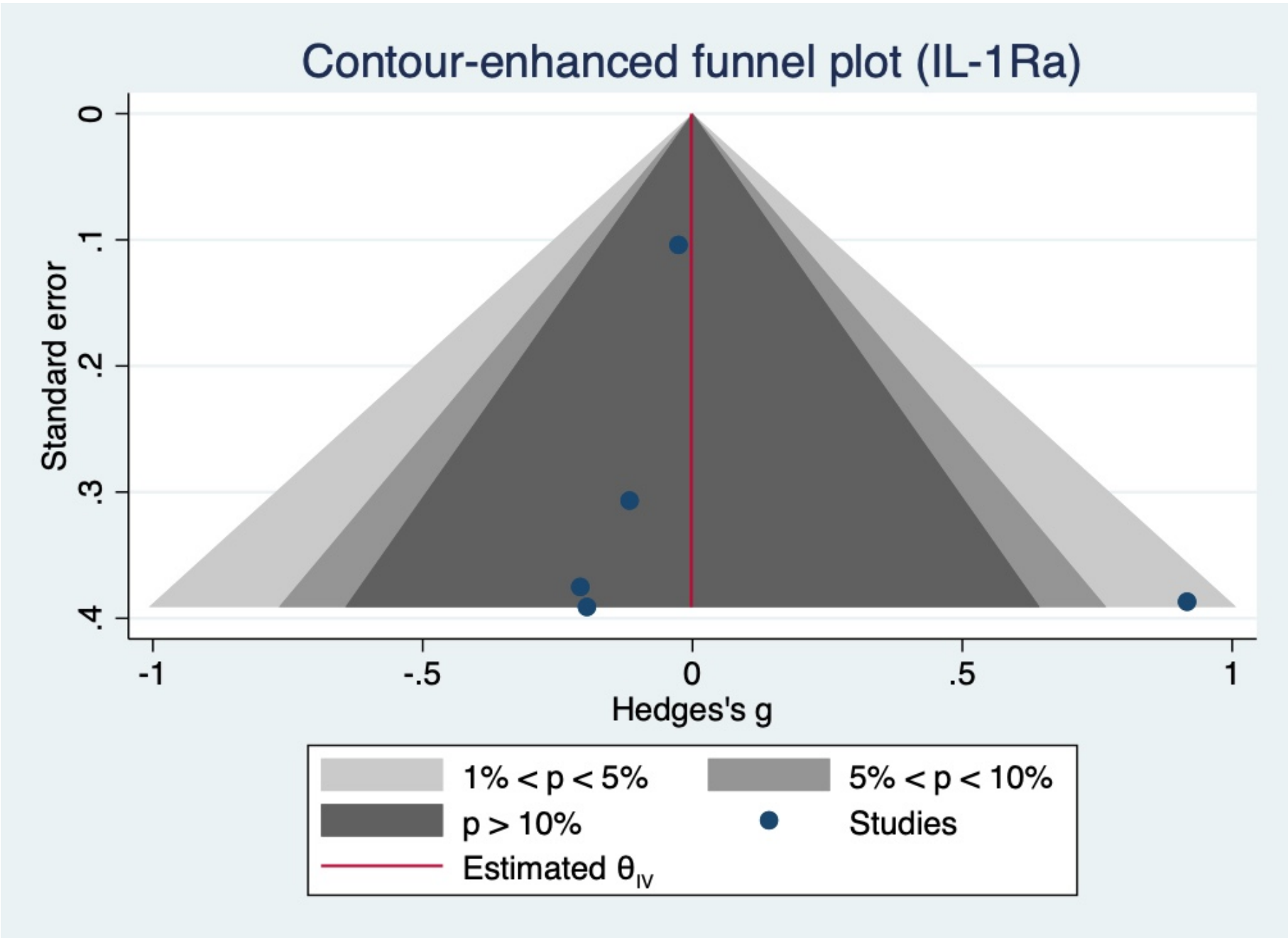
Contour-enhanced funnel plot (IL-1Ra)
Standard error
0
.1
.2
.3
.4
-1
-.5
0
.5
1
Hedges's g
1% < p < 5%
5% < p < 10%
p > 10%
Studies
Estimated θIV

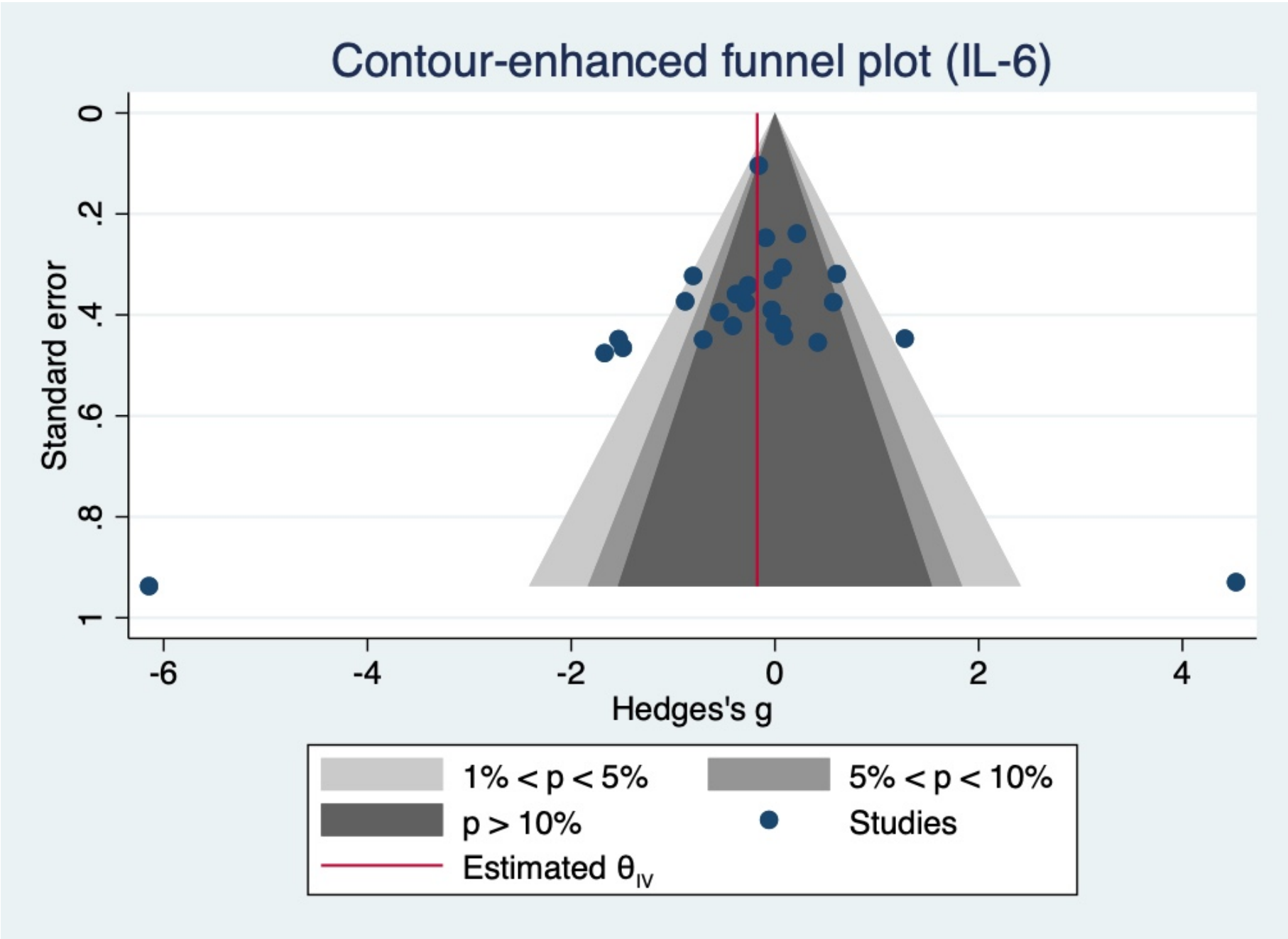
Contour-enhanced funnel plot (IL-6)
Standard error
0
.2
.4
.6
.8
1
-6
-4
-2
0
2
4
Hedges's g
1% < p < 5%
5% < p < 10%
p > 10%
Studies
Estimated θIV

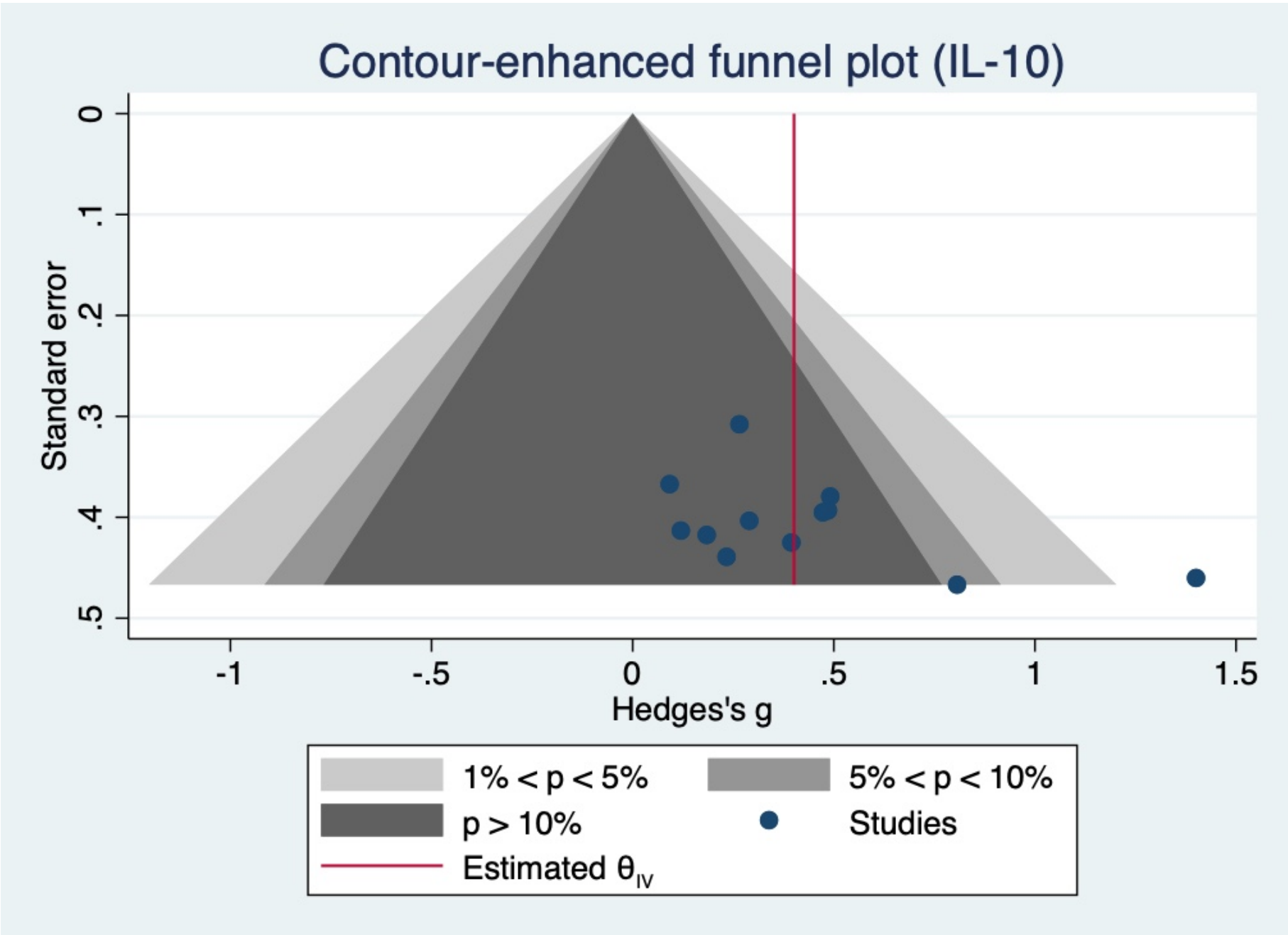
Contour-enhanced funnel plot (IL-10)
Standard error
0
.1
.2
.3
.4
.5
-1
-.5
0
.5
1
1.5
Hedges's g
1% < p < 5%
5% < p < 10%
p > 10%
Studies
Estimated θIV

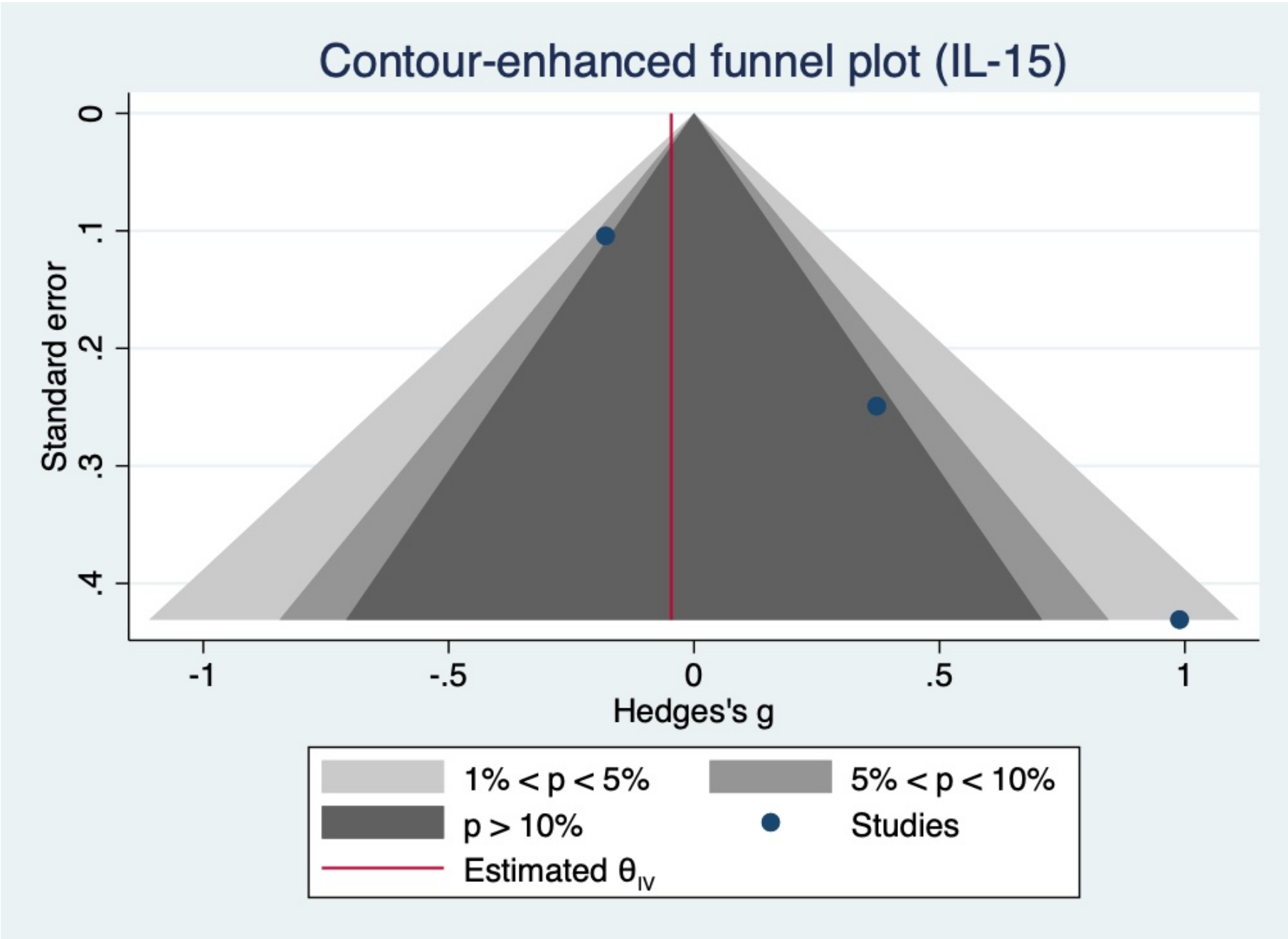
Contour-enhanced funnel plot (IL-15)
Standard error
0
.1
.2
.3
.4
-1
-.5
0
.5
1
Hedges's g
1% < p < 5%
5% < p < 10%
p > 10%
Studies
Estimated θIV

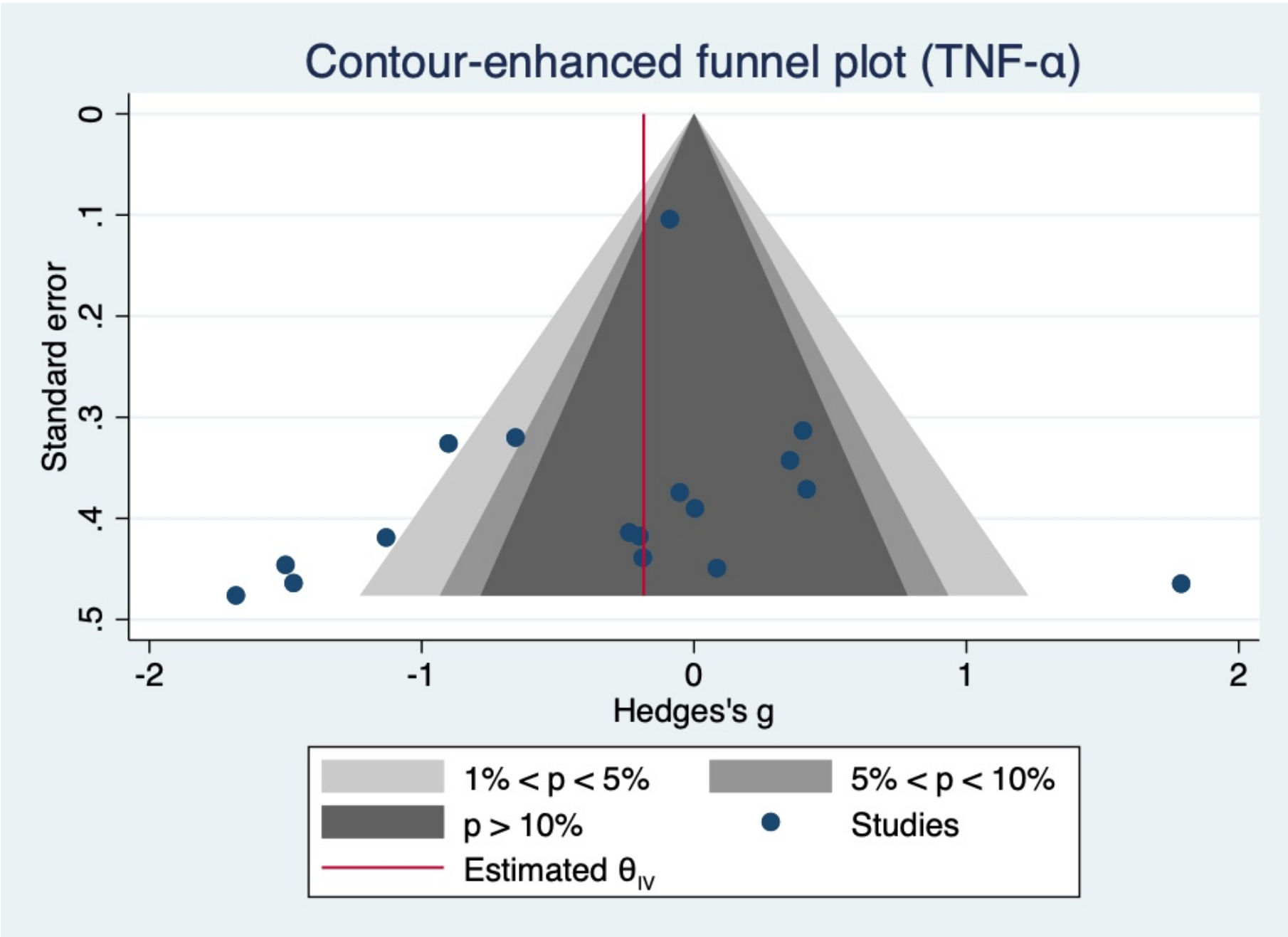
Contour-enhanced funnel plot (TNF-α)
Standard error
0
.1
.2
.3
.4
.5
-2
-1
0
1
2
Hedges's g
1% < p < 5%
5% < p < 10%
p > 10%
Studies
Estimated θIV

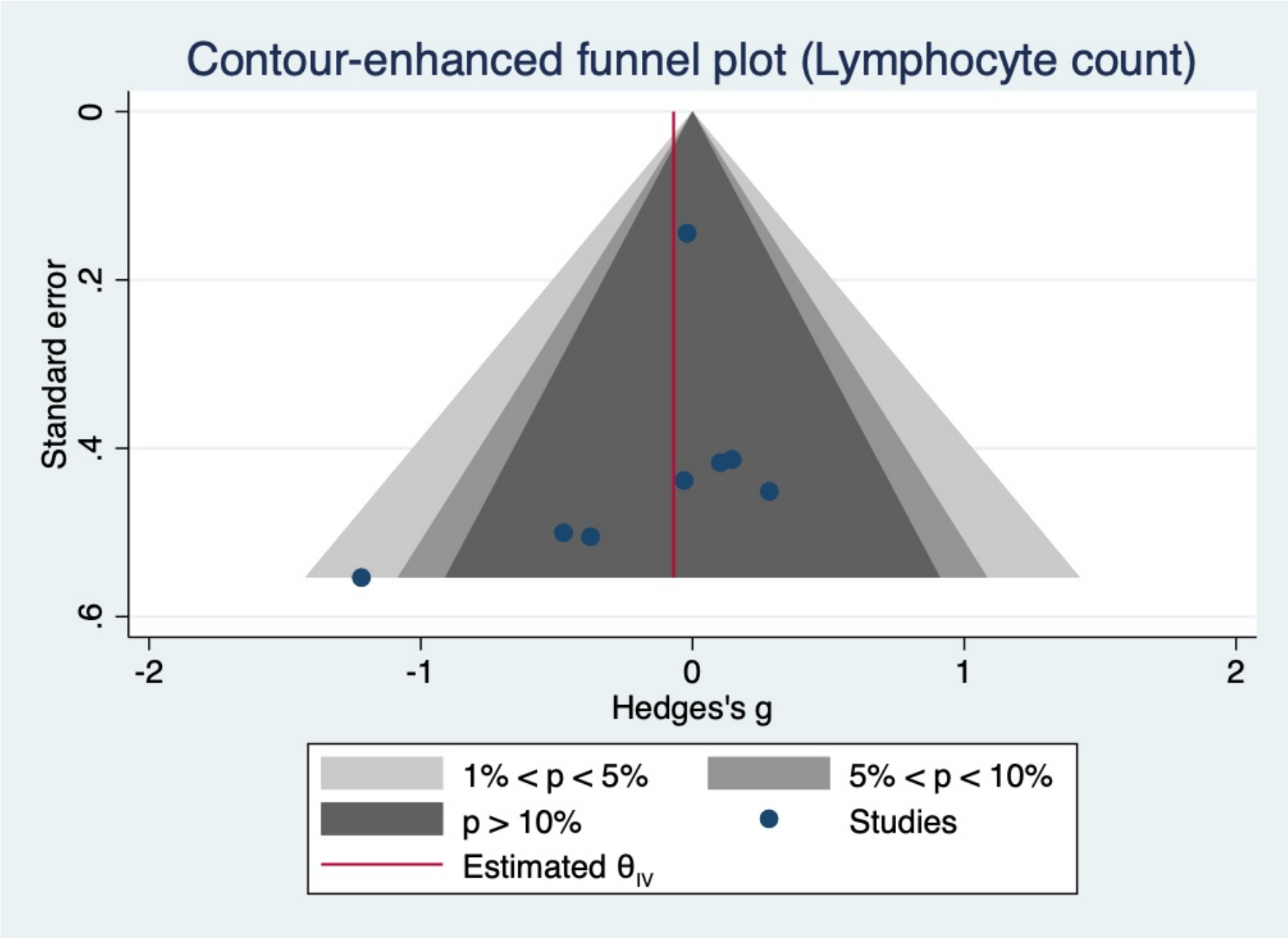

Contour-enhanced funnel plot (Lymphocyte count)
Standard error
0
.2
.4
.6
-2
-1
0
1
2
Hedges's g
1% < p < 5%
5% < p < 10%
p > 10%
Studies
Estimated θIV

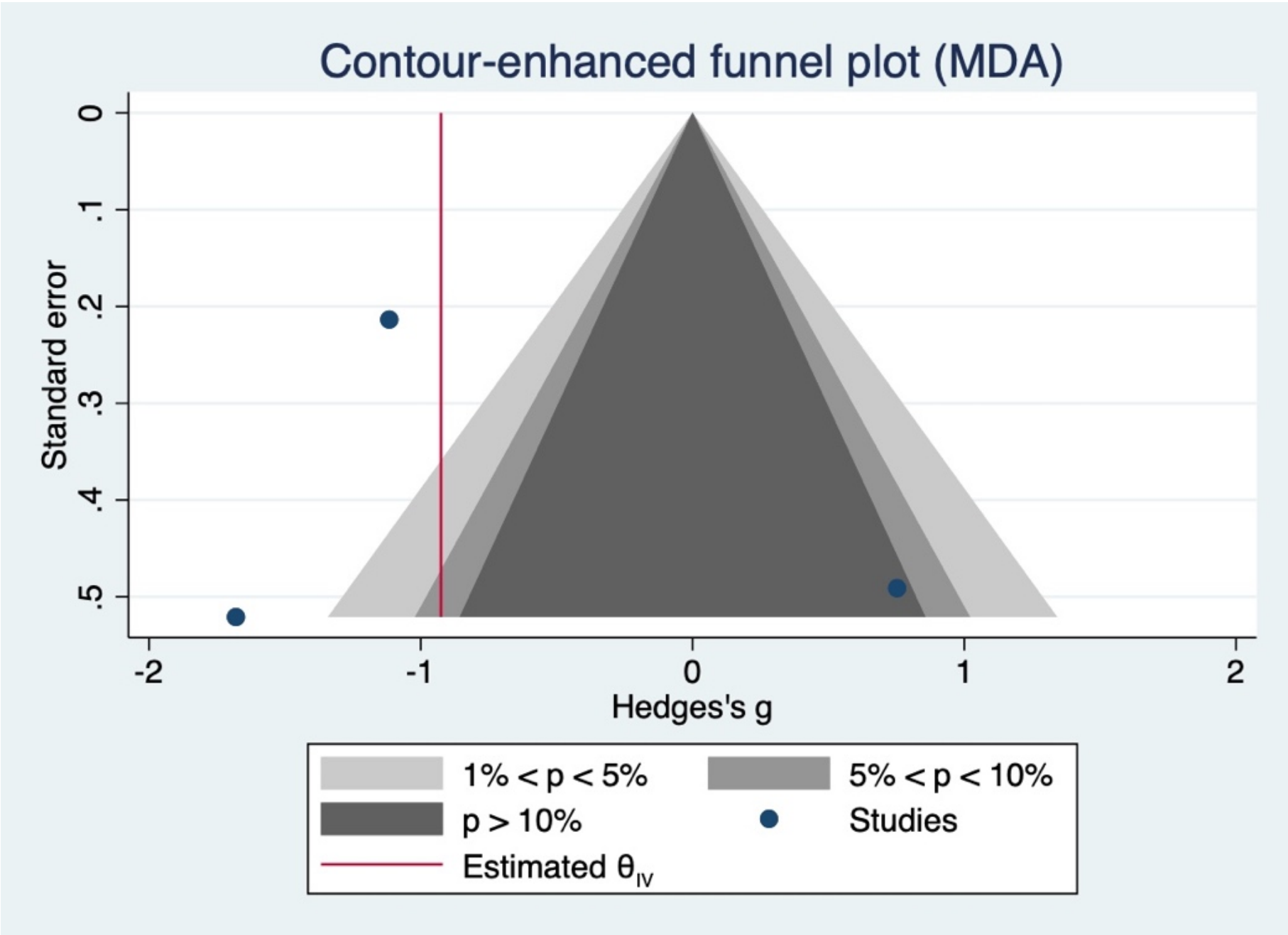

Contour-enhanced funnel plot (MDA)
Standard error
0
.1
.2
.3
.4
.5
-2
-1
0
1
2
Hedges's g
1% < p < 5%
5% < p < 10%
p > 10%
Studies
Estimated θIV

Figures S19-S28. Leave-one-out sensitivity analyses

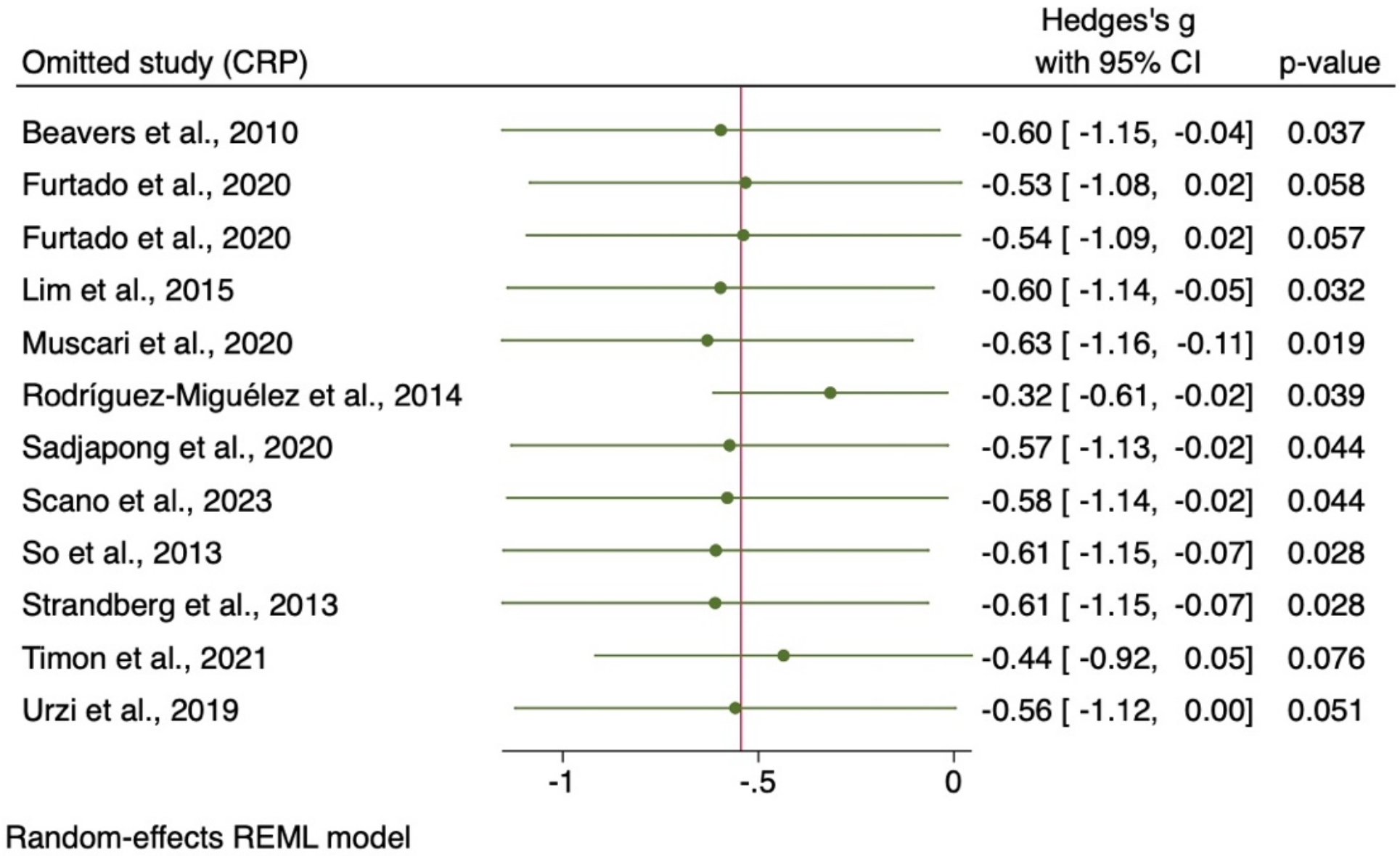

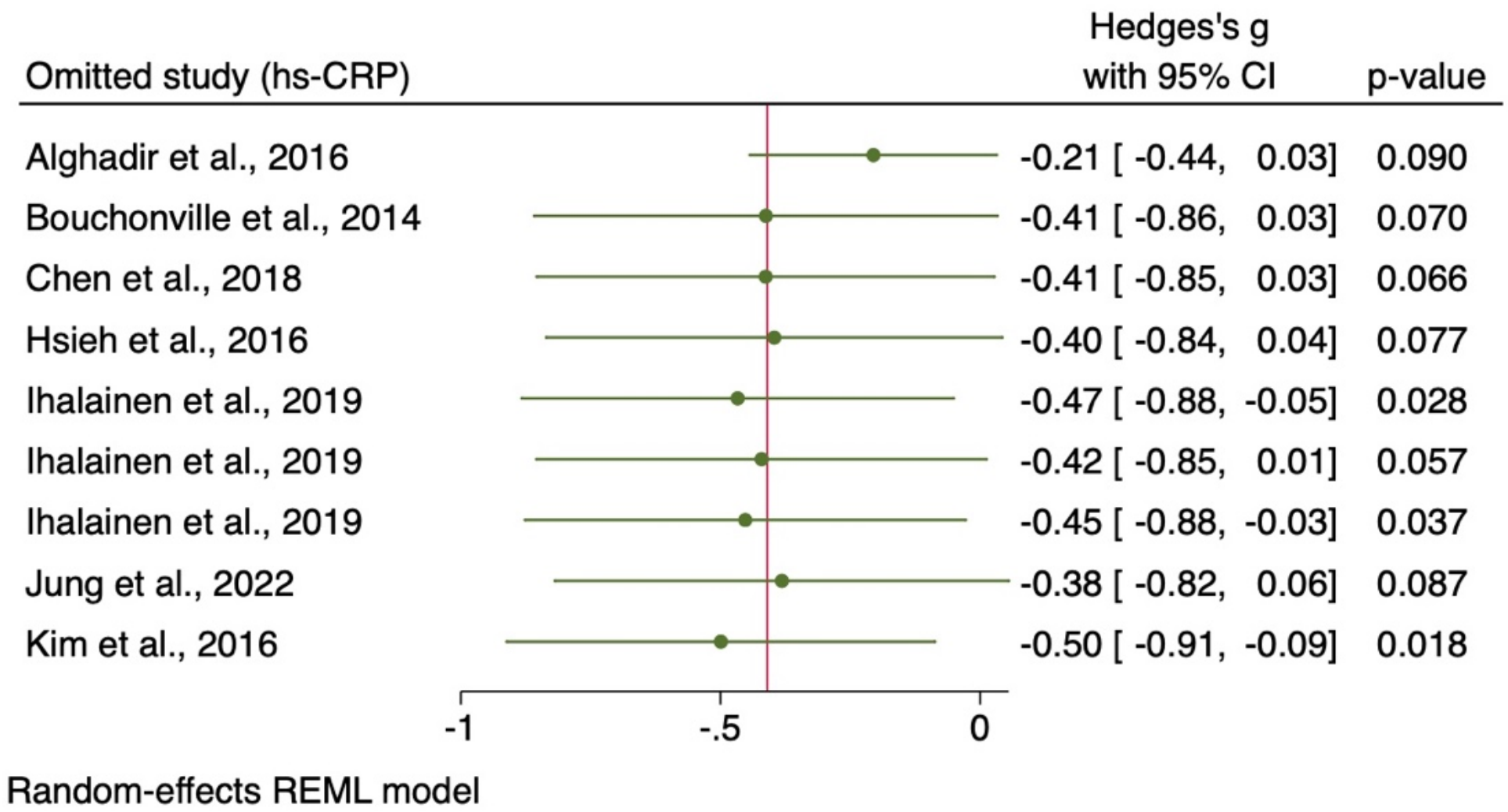

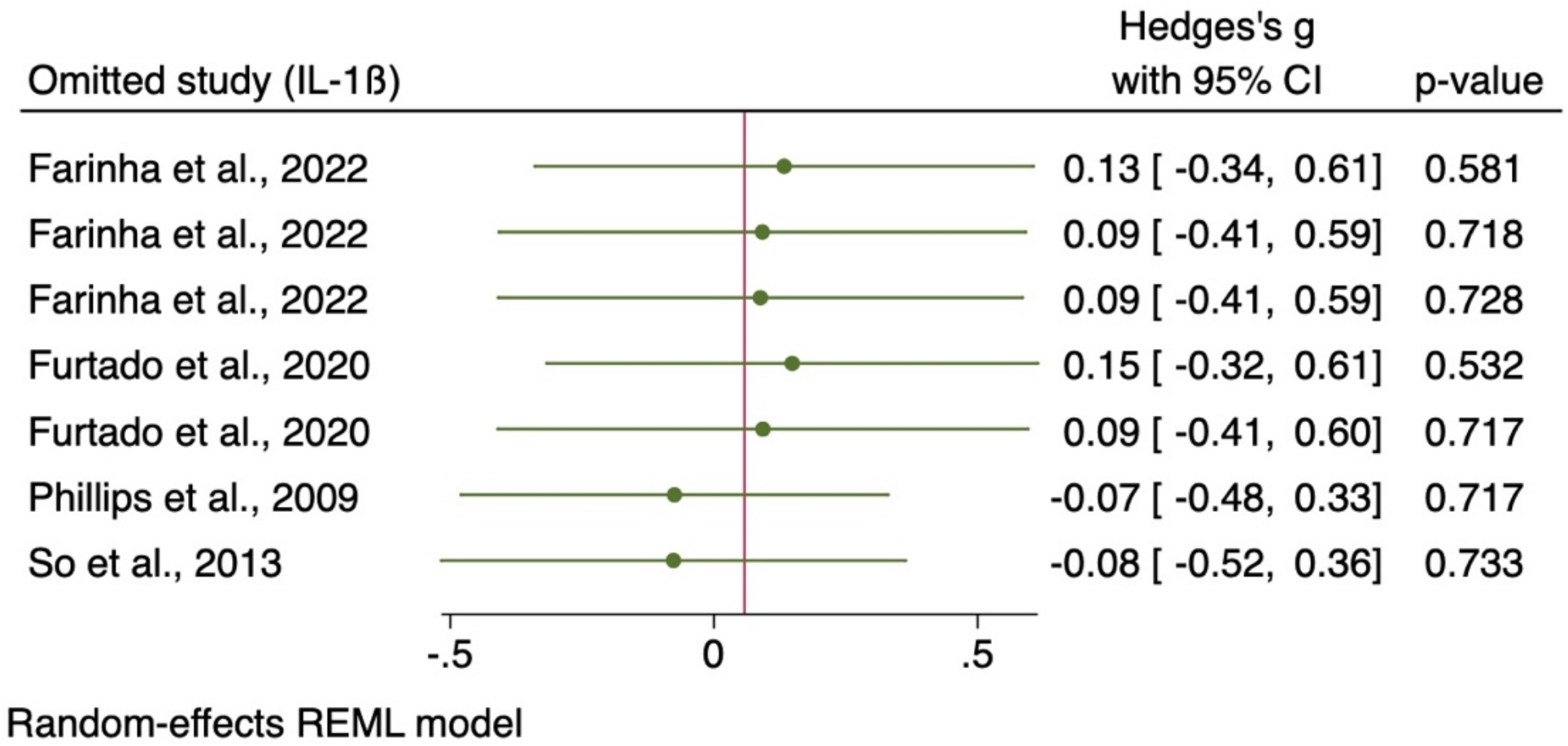

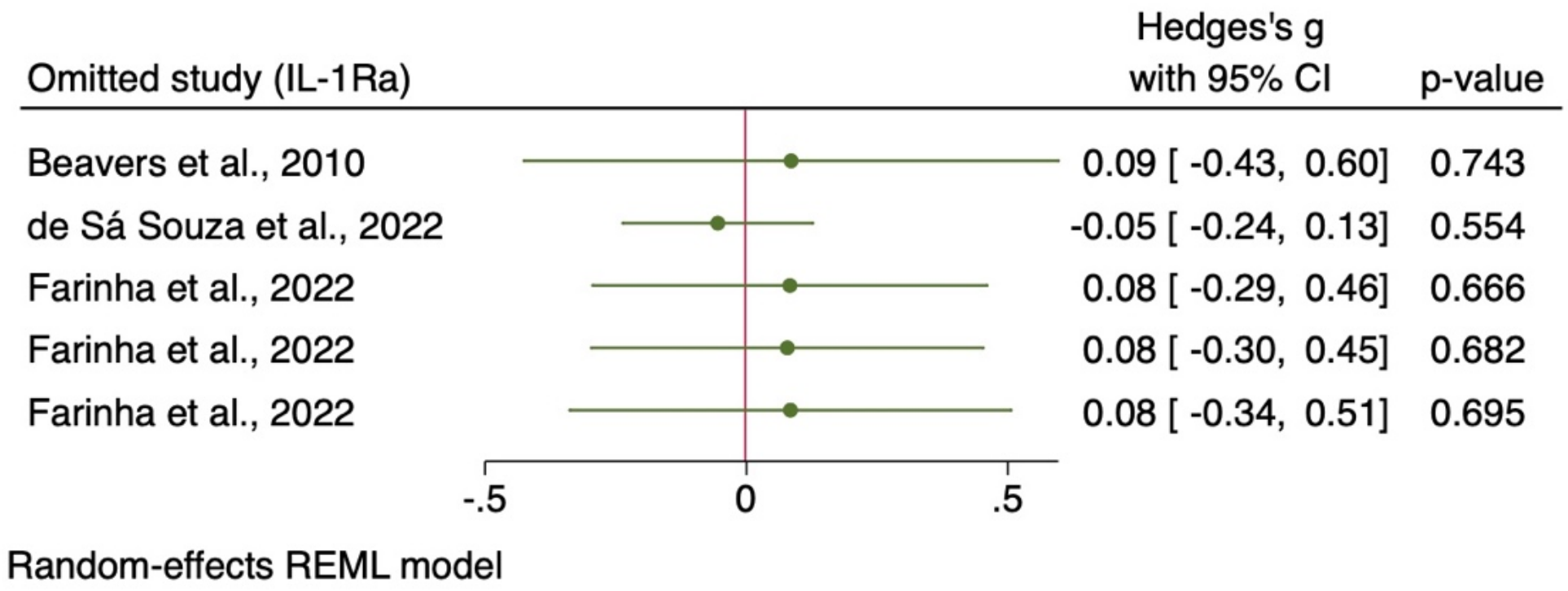
Omitted study (IL-1Ra)
Hedges's g with 95% CI
p-value
Beavers et al., 2010 0.09 [-0.43, 0.60] 0.743
de Sá Souza et al., 2022 -0.05 [-0.24, 0.13] 0.554
Farinha et al., 2022 0.08 [-0.29, 0.46] 0.666
Farinha et al., 2022 0.08 [-0.30, 0.45] 0.682
Farinha et al., 2022 0.08 [-0.34, 0.51] 0.695
-.5
0
.5
Random-effects REML model

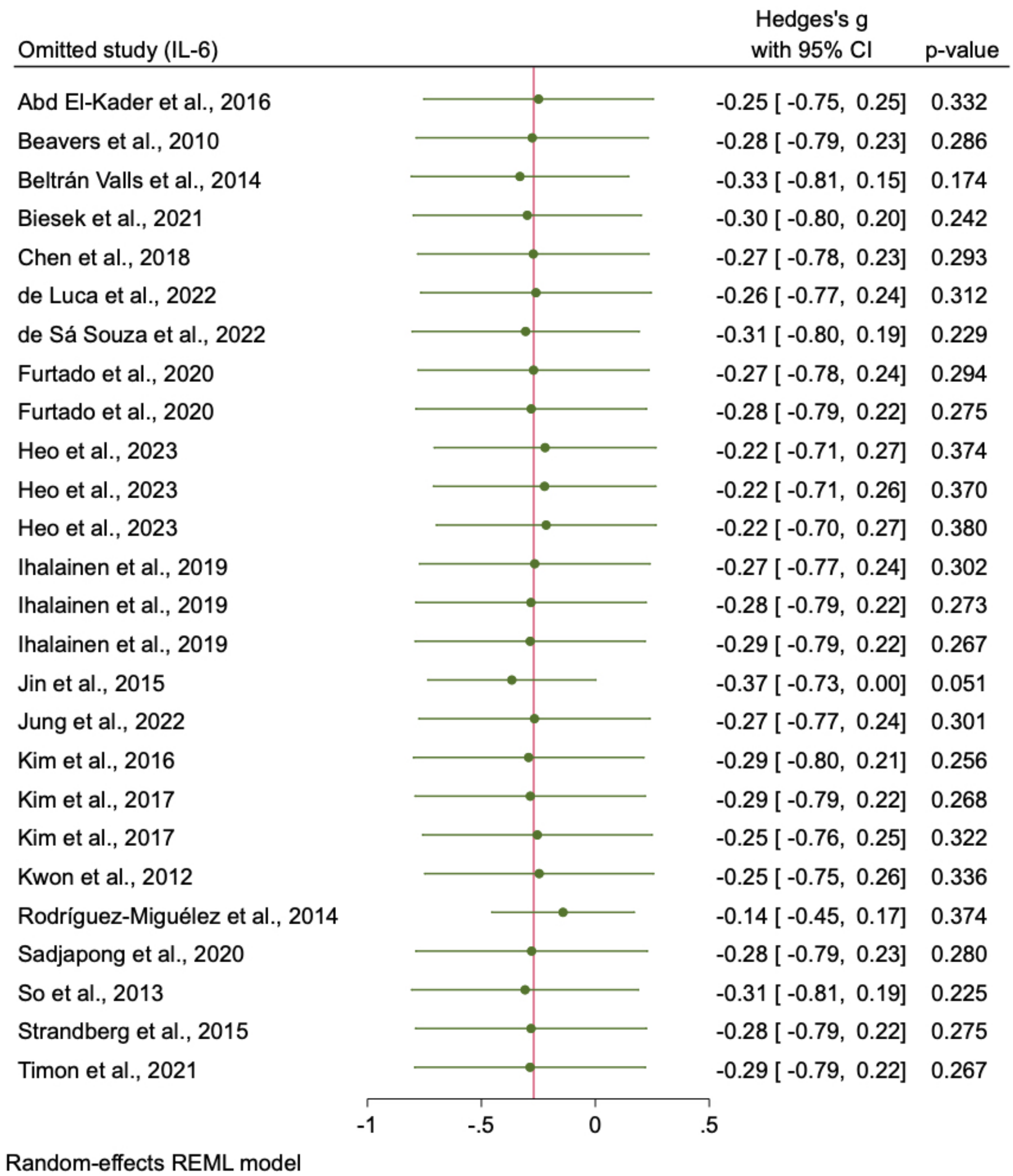
Omitted study (IL-6)
Hedges's g with 95% CI
p-value
Abd El-Kader et al., 2016 -0.25 [-0.75, 0.25] 0.332
Beavers et al., 2010 -0.28 [-0.79, 0.23] 0.286
Beltrán Valls et al., 2014 -0.33 [-0.81, 0.15] 0.174
Biesek et al., 2021 -0.30 [-0.80, 0.20] 0.242
Chen et al., 2018 -0.27 [-0.78, 0.23] 0.293
de Luca et al., 2022 -0.26 [-0.77, 0.24] 0.312
de Sá Souza et al., 2022 -0.31 [-0.80, 0.19] 0.229
Furtado et al., 2020 -0.27 [-0.78, 0.24] 0.294
Furtado et al., 2020 -0.28 [-0.79, 0.22] 0.275
Heo et al., 2023 -0.22 [-0.71, 0.27] 0.374
Heo et al., 2023 -0.22 [-0.71, 0.26] 0.370
Heo et al., 2023 -0.22 [-0.70, 0.27] 0.380
Ihalainen et al., 2019 -0.27 [-0.77, 0.24] 0.302
Ihalainen et al., 2019 -0.28 [-0.79, 0.22] 0.273
Ihalainen et al., 2019 -0.29 [-0.79, 0.22] 0.267
Jin et al., 2015 -0.37 [-0.73, 0.00] 0.051
Jung et al., 2022 -0.27 [-0.77, 0.24] 0.301
Kim et al., 2016 -0.29 [-0.80, 0.21] 0.256
Kim et al., 2017 -0.29 [-0.79, 0.22] 0.268
Kim et al., 2017 -0.25 [-0.76, 0.25] 0.322
Kwon et al., 2012 -0.25 [-0.75, 0.26] 0.336
Rodríguez-Miguélez et al., 2014 -0.14 [-0.45, 0.17] 0.374
Sadjapong et al., 2020 -0.28 [-0.79, 0.23] 0.280
So et al., 2013 -0.31 [-0.81, 0.19] 0.225
Strandberg et al., 2015 -0.28 [-0.79, 0.22] 0.275
Timon et al., 2021 -0.29 [-0.79, 0.22] 0.267
-1
-.5
0
.5
Random-effects REML model

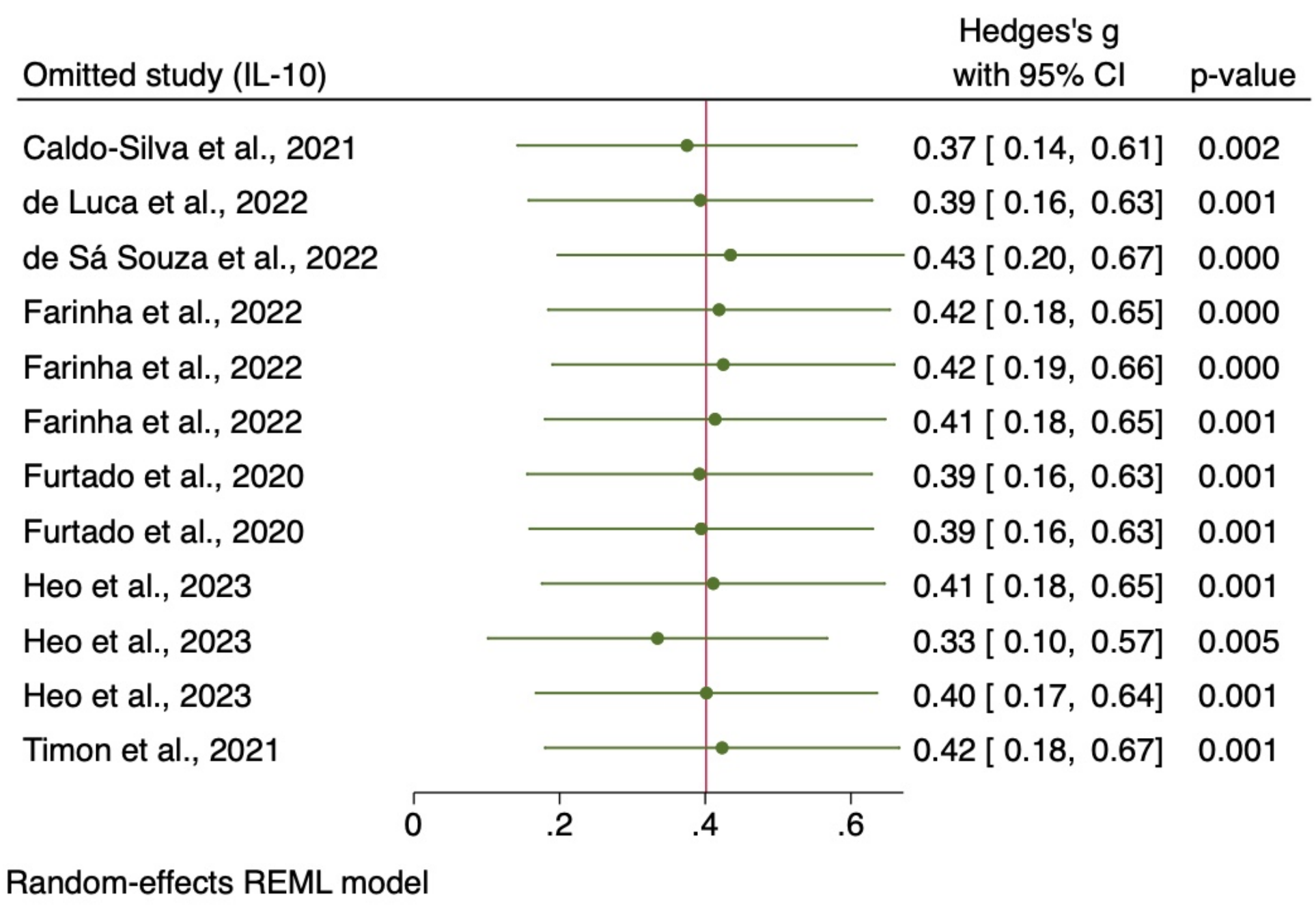
Omitted study (IL-10)
Hedges's g with 95% CI
p-value
Caldo-Silva et al., 2021 0.37 [0.14, 0.61] 0.002
de Luca et al., 2022 0.39 [0.16, 0.63] 0.001
de Sá Souza et al., 2022 0.43 [0.20, 0.67] 0.000
Farinha et al., 2022 0.42 [0.18, 0.65] 0.000
Farinha et al., 2022 0.42 [0.19, 0.66] 0.000
Farinha et al., 2022 0.41 [0.18, 0.65] 0.001
Furtado et al., 2020 0.39 [0.16, 0.63] 0.001
Furtado et al., 2020 0.39 [0.16, 0.63] 0.001
Heo et al., 2023 0.41 [0.18, 0.65] 0.001
Heo et al., 2023 0.33 [0.10, 0.57] 0.005
Heo et al., 2023 0.40 [0.17, 0.64] 0.001
Timon et al., 2021 0.42 [0.18, 0.67] 0.001
0
.2
.4
.6
Random-effects REML model

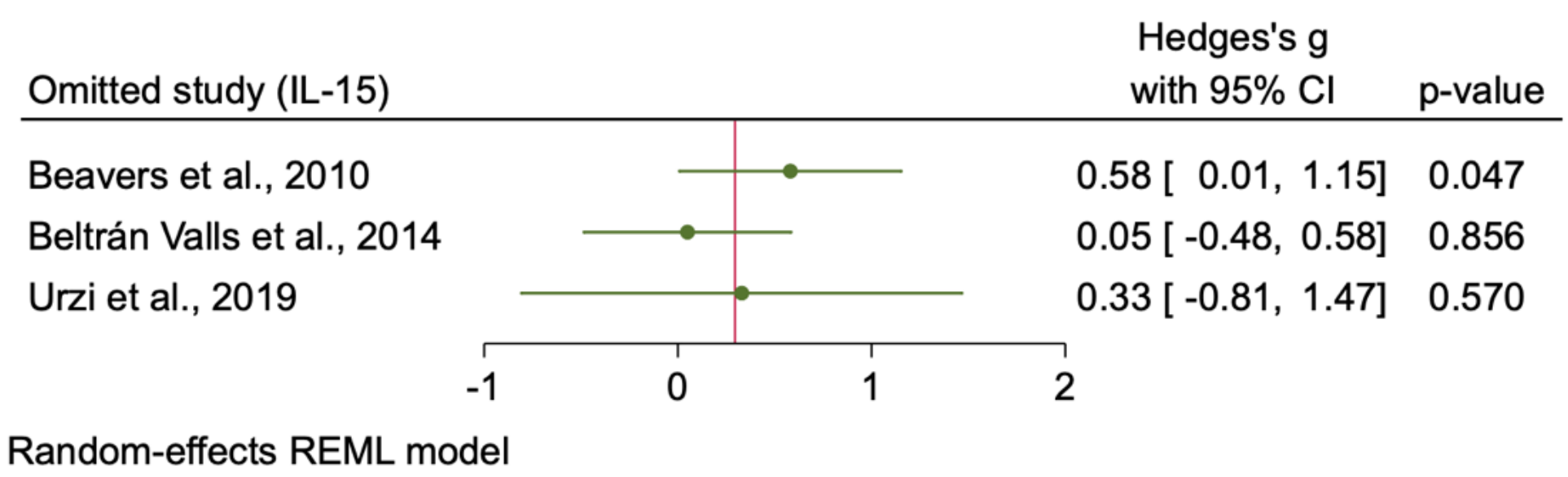
Omitted study (IL-15)
Hedges's g with 95% CI
p-value
Beavers et al., 2010 0.58 [0.01, 1.15] 0.047
Beltrán Valls et al., 2014 0.05 [-0.48, 0.58] 0.856
Urzi et al., 2019 0.33 [-0.81, 1.47] 0.570
-1
0
1
2
Random-effects REML model

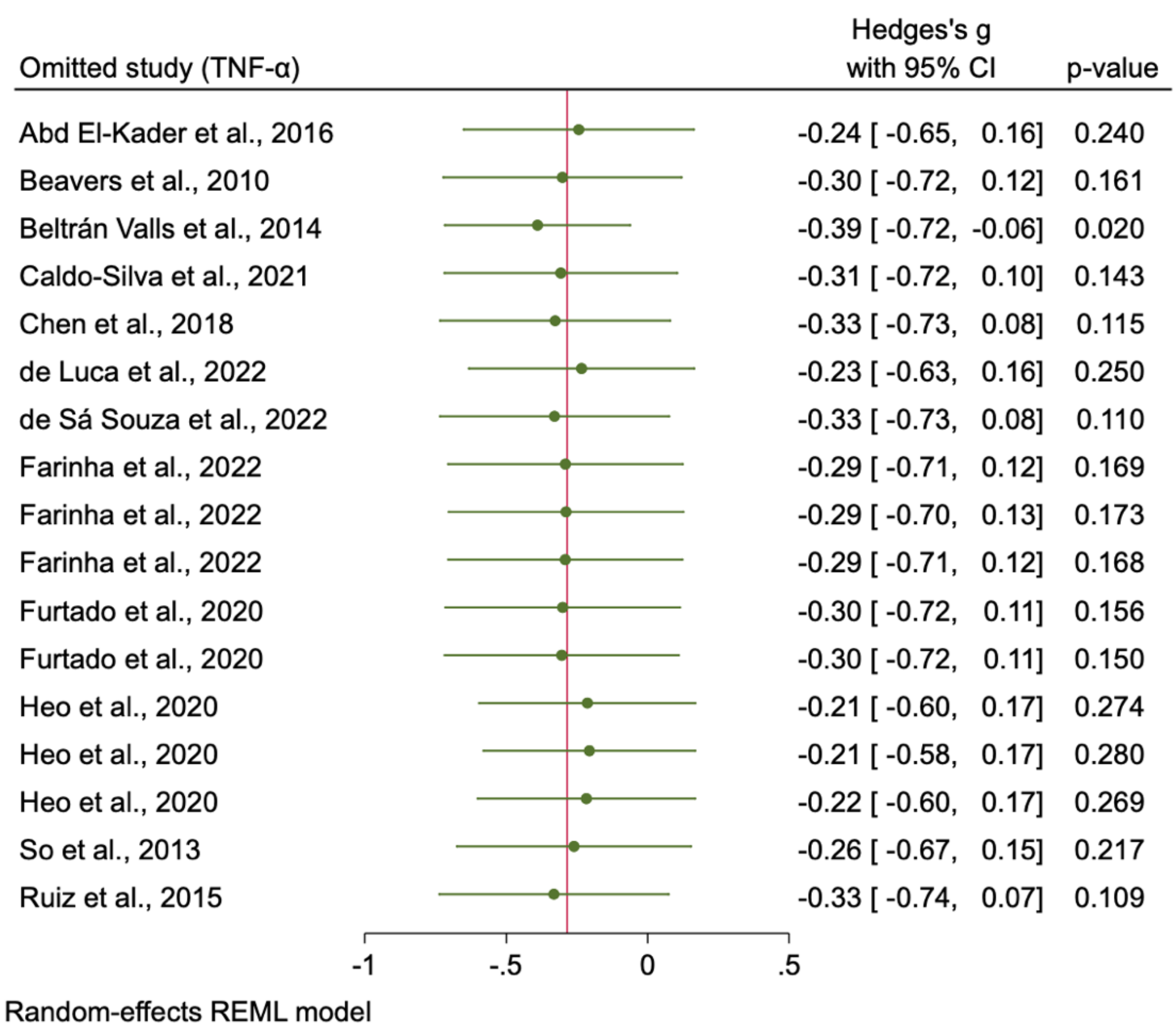
Omitted study (TNF-α)
Hedges's g with 95% CI
p-value
Abd El-Kader et al., 2016 -0.24 [-0.65, 0.16] 0.240
Beavers et al., 2010 -0.30 [-0.72, 0.12] 0.161
Beltrán Valls et al., 2014 -0.39 [-0.72, -0.06] 0.020
Caldo-Silva et al., 2021 -0.31 [-0.72, 0.10] 0.143
Chen et al., 2018 -0.33 [-0.73, 0.08] 0.115
de Luca et al., 2022 -0.23 [-0.63, 0.16] 0.250
de Sá Souza et al., 2022 -0.33 [-0.73, 0.08] 0.110
Farinha et al., 2022 -0.29 [-0.71, 0.12] 0.169
Farinha et al., 2022 -0.29 [-0.70, 0.13] 0.173
Farinha et al., 2022 -0.29 [-0.71, 0.12] 0.168
Furtado et al., 2020 -0.30 [-0.72, 0.11] 0.156
Furtado et al., 2020 -0.30 [-0.72, 0.11] 0.150
Heo et al., 2020 -0.21 [-0.60, 0.17] 0.274
Heo et al., 2020 -0.21 [-0.58, 0.17] 0.280
Heo et al., 2020 -0.22 [-0.60, 0.17] 0.269
So et al., 2013 -0.26 [-0.67, 0.15] 0.217
Ruiz et al., 2015 -0.33 [-0.74, 0.07] 0.109
-1
-.5
0
.5
Random-effects REML model

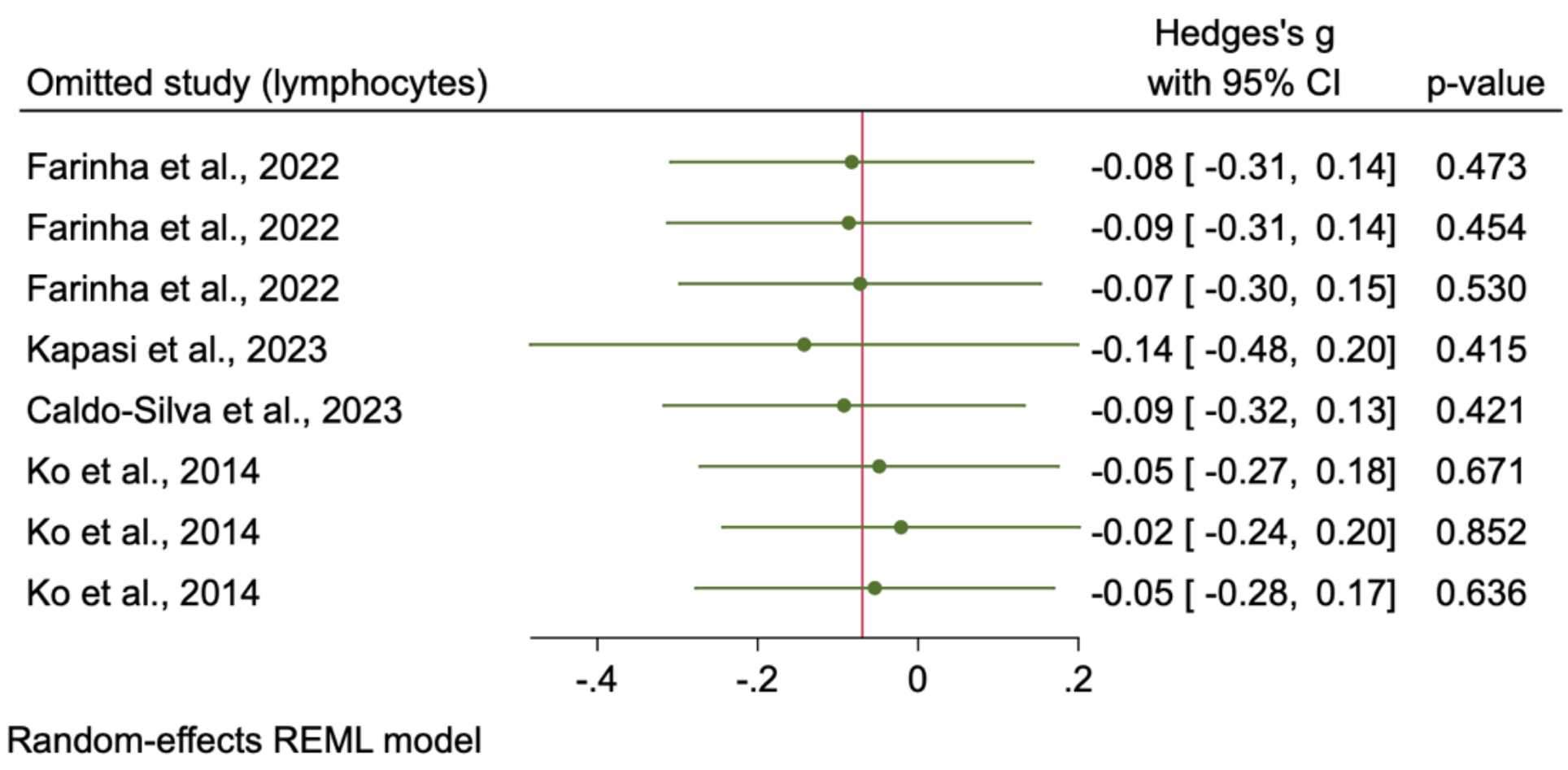
Omitted study (lymphocytes)
Hedges's g with 95% CI
p-value
Farinha et al., 2022 -0.08 [-0.31, 0.14] 0.473
Farinha et al., 2022 -0.09 [-0.31, 0.14] 0.454
Farinha et al., 2022 -0.07 [-0.30, 0.15] 0.530
Kapasi et al., 2023 -0.14 [-0.48, 0.20] 0.415
Caldo-Silva et al., 2023 -0.09 [-0.32, 0.13] 0.421
Ko et al., 2014 -0.05 [-0.27, 0.18] 0.671
Ko et al., 2014 -0.02 [-0.24, 0.20] 0.852
Ko et al., 2014 -0.05 [-0.28, 0.17] 0.636
-.4
-.2
0
.2
Random-effects REML model

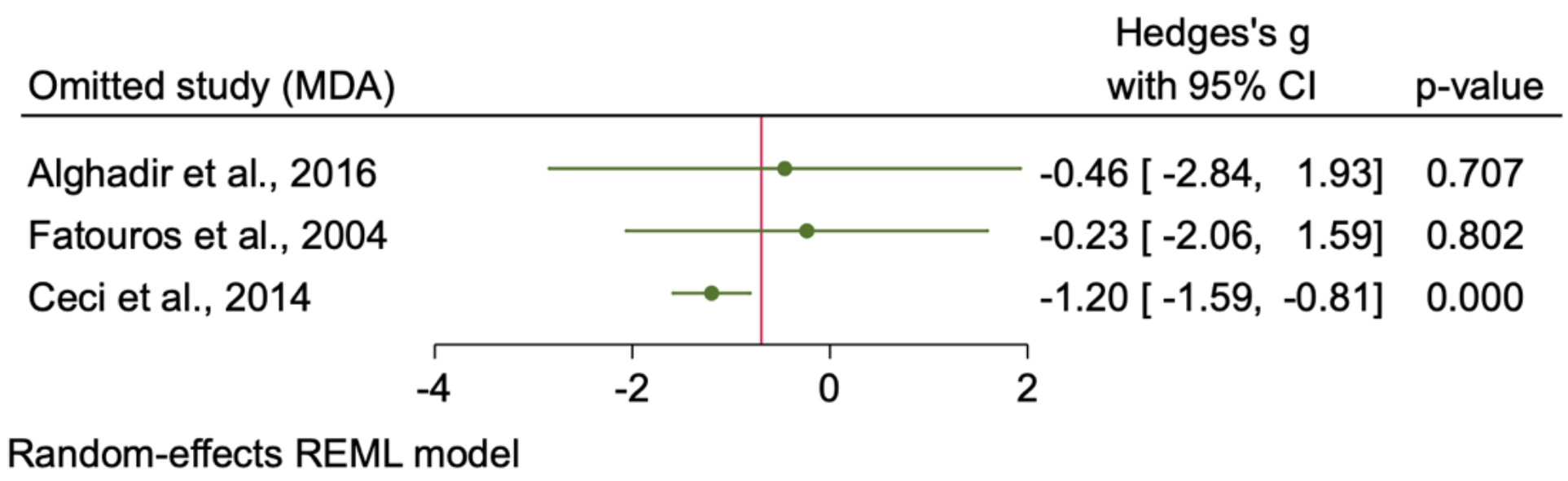
Omitted study (MDA)
Hedges's g with 95% CI
p-value
Alghadir et al., 2016 -0.46 [-2.84, 1.93] 0.707
Fatouros et al., 2004 -0.23 [-2.06, 1.59] 0.802
Ceci et al., 2014 -1.20 [-1.59, -0.81] 0.000
-4
-2
0
2
Random-effects REML model
